# Anti-inflammatory diets and mental health: a scoping review of randomized controlled trials and systematic evidence syntheses

**DOI:** 10.3389/fnut.2026.1795350

**Published:** 2026-04-13

**Authors:** Meredith L. Sprengel, Ryesa Mansoor, Nikoleta Latsou, Samantha Allen, Olivia Lubarsky, Kylie Mark, Leah Gizzi, Cassandra Vieten

**Affiliations:** 1 John W. Brick Mental Health Foundation, Timonium, MD, United States; 2 Cognitive Psychology Unit, Institute of Psychology, Leiden University, Leiden, Netherlands; 3 George Washington University School of Medicine and Health Sciences, Counseling Psychology Program, Washington, DC, United States; 4 Department of Psychology, Frostburg State University, Frostburg, MD, United States; 5 Centers for Integrative Health, Department of Family Medicine, University of California, San Diego, San Diego, CA, United States

**Keywords:** anti-inflammatory diets, anxiety, DASH, depression, dietary patterns, Mediterranean diet, mental health, scoping review

## Abstract

**Background:**

Inflammation has been proposed as a biological pathway linking diet to mental health. Anti-inflammatory dietary patterns, including the Mediterranean and DASH diets, may reduce systemic inflammation and thereby influence mental health outcomes. However, evidence across study designs has not been comprehensively synthesized.

**Objective:**

This scoping review aimed to identify randomized controlled trials (RCTs) and systematic evidence syntheses to summarize the effects of anti-inflammatory dietary interventions or patterns on mental health outcomes in adults.

**Methods:**

Searches were conducted in scientific databases through February 2025. Eligible RCTs included adult participants receiving an anti-inflammatory dietary intervention with reported mental health outcomes. Eligible systematic evidence syntheses included systematic reviews with or without meta-analysis, scoping reviews, and umbrella reviews examining associations between anti-inflammatory diets and mental health. Article screening and review were conducted in duplicate, and data extraction followed standardized procedures appropriate for each study type.

**Results:**

A total of 21,923 records were identified, of which 42 RCTs and 23 systematic evidence syntheses met the inclusion criteria. Of the 42 RCTs, 28 reported significantly greater improvements in at least one mental health outcome compared with controls following an anti-inflammatory dietary intervention. Most trials evaluated Mediterranean diet-based interventions. Across RCTs, improvements were most consistently observed for depressive symptoms, while findings for anxiety, mood, stress, and quality of life were mixed. Across the 23 systematic evidence syntheses, findings for depression outcomes were generally positive, with multiple reviews reporting inverse associations between adherence to anti-inflammatory dietary patterns and depression risk or symptom severity. Evidence for anxiety outcomes across reviews was more heterogeneous and less consistent.

**Conclusion:**

Anti-inflammatory dietary patterns, particularly the Mediterranean diet, are associated with improvements in depressive symptoms across randomized controlled trials and systematic evidence syntheses, with less consistent findings for anxiety and other mental health outcomes. Benefits were most consistently observed among individuals with existing mental health symptoms. Interpretation is limited by heterogeneity in dietary interventions, multimodal study designs, populations, and outcome measures. Future research using standardized dietary protocols, longer follow-up periods, and biomarker assessment is needed to strengthen causal inference and clarify underlying mechanisms.

## 1 Introduction

Inflammation is an essential aspect of the body’s biological healing process. Acute inflammation (a rapid and localized reaction characterized by increased blood flow, swelling, and the mobilization of immune cells) is our natural immune system’s response to injury or harmful stimuli (1). However, uncontrolled, acute inflammatory responses can develop into systemic low-grade chronic inflammation in the body (2). Low-grade chronic inflammation is involved in the pathogenesis of many illnesses and conditions such as cancer, cardiovascular disease, autoimmune disease, and cognitive decline (3–5).

Studies have also linked low grade chronic inflammation to the pathogenesis of mental health disorders, such as depression, bipolar disorder, and schizophrenia (4, 6–10). Notably, clinical studies have confirmed associations of higher levels of circulating proinflammatory biomarkers, such as tumor necrosis factor, C-reactive protein, and inflammatory cytokine interleukin-6 (IL-6) with symptoms of depression, major depressive disorder, and anxiety disorders (11–15). Additionally, during a normal immune response, the production of pro-inflammatory cytokines directly results in behavioral symptoms known as “sickness behavior” (3, 9). These symptoms take the form of depressive-like behaviors, including fatigue, decreased appetite, concentration problems, and anhedonia (16). Similarly, there is evidence demonstrating associations between antidepressant efficacy and inflammatory cytokine levels, where lower cytokine levels predict better outcomes to antidepressant treatment therapies (9, 17–19).

Chronic stress is also linked to chronic inflammation. Environmental, physiological, or psychological stressors result in various hormonal and neurochemical changes throughout the body, mainly by activating the sympathetic nervous system and the hypothalamic–pituitary–adrenal (HPA) axis (20, 21). Chronic stress is associated with increased levels of proinflammatory cytokines, (activated by the body’s inflammatory response by way of the HPA axis) and is also a risk factor for various physical diseases and morbidities including but not limited to: cardiovascular diseases, metabolic diseases, and neurodegenerative disorders (22–25). The role of inflammation in mental illness may be associated with immune activation in the central and peripheral nervous system (9, 26).

Several animal studies suggest that inflammatory cytokines may directly affect the brain, and therefore impact psychological function, through several possible pathways including altering neurotransmitter metabolism, dysregulating the HPA, increasing oxidative stress, or reducing synaptic plasticity (11, 27–30). Additionally, this link between stress and inflammation and mental health outcomes is thought to be bidirectional (24, 31). The HPA axis, when activated in response to stress, is associated with heightened immune response and cytokine production, while inflammatory cytokines during periods of stress in turn activate the HPA axis and increase inflammation in depression (32, 33).

Considering empirical support for the role of stress and inflammation in contributing to mental health issues, the shift toward examining behavioral lifestyle approaches that target inflammation has become an important development in mental health research. One such behavioral approach includes dietary intervention through consumption of anti-inflammatory foods and reduced consumption of pro-inflammatory foods. Anti-inflammatory diets, such as the Mediterranean Diet (MedDiet) and Dietary Approaches to Stop Hypertension (DASH) Diet, adhere to nutrients and foods that contain anti-inflammatory properties (34–36). Examples of these foods include whole grains, fish high in omega-3 polyunsaturated fats, legumes, nuts, fruits, vegetables, and olive oil (37, 38). These diets differ from typical Western diets which are higher in consumption of refined grains, red meat, and lower consumption of fruits and vegetables (39). Consumption of anti-inflammatory foods could potentially improve mental health outcomes.

Recent controlled studies have started to demonstrate the potential role anti-inflammatory diets may play a role in addressing mental health conditions and outcomes. The purpose of this review is to summarize and evaluate the existing peer-reviewed literature on anti-inflammatory dietary treatments and mental health outcomes. Here, we identified randomized controlled trials (RCTs) and systematic evidence synthesis, including systematic reviews (SRs), systematic reviews including a meta-analysis (SR + MA), umbrella reviews (UMRs), and scoping reviews (SCRs), evaluating the efficacy of anti-inflammatory diets and specific components of these diets on mental health and well-being outcomes including depression, mood, anxiety, and stress.

## 2 Methods

### 2.1 Identifying anti-inflammatory diets

There is no official “anti-inflammatory” dietary designation. More research is needed to define what key components are needed in a diet to most effectively address inflammation. However, research has indicated that certain foods and dietary patterns are associated with decreased inflammation (40).

Most anti-inflammatory diets do not focus on calorie count, portion size, or consumption of a few particular nutrients or foods, but rather emphasize the need to consume a variety of anti-inflammatory foods daily (41). Anti-inflammatory diets tend to include (42):

- Fruits
- Vegetables
- Legumes
- High fiber whole grains
- Spices (e.g.,turmeric, cumin)
- Low amounts of alcohol (wine, beer)
- Polyunsaturated omega-3 fats (salmon, flax seeds)
- Monounsaturated fats (olive oil, nuts)
- Moderate amounts of lean animal protein (poultry)

Regions where people live the longest, sometimes referred to as “blue zones,” are known for traditionally consuming diets high in these anti-inflammatory foods (43–45). These diets are often referred to as Mediterranean style diets (42).

When identifying the diet types to be included in this review, we consulted with nutrition and dietary experts and performed an initial review of the published literature on anti-inflammatory dietary patterns prior to executing the formal search strategy. Classification of anti-inflammatory dietary patterns relies on composite adherence indices and their associations with inflammatory biomarkers, rather than fixed nutritional thresholds, which may introduce some heterogeneity across included studies. For the purposes of this scoping review, dietary patterns were included if they were explicitly described in the peer reviewed literature as anti-inflammatory or consistently evaluated in relation to inflammatory outcomes.

To identify the diets included in this review, we consulted with nutrition and dietary experts and performed an initial review of the published literature on anti-inflammatory diets before executing the search. For the purpose of the review, we included the following diets:

- DASH Diet
- Mediterranean Diet (MedDiet)
- Mediterranean-DASH Intervention for Neurodegenerative Delay (MIND) Diet
- Crete Diet
- Nordic Dietary Pattern
- Bespoke/unnamed anti-inflammatory diets- includes diets identified as targeting inflammation

### 2.2 Scoping review protocol

Our protocol was devised using the Preferred Reporting Items for Systematic Reviews and Meta-analysis extension for ScRs (PRISMA-ScR) (46). The PRISMA-ScR reporting checklist is available in Supplementary Table S2.

### 2.3 Inclusion and exclusion criteria

This scoping review was designed to address the following questions:

“What RCTs have been conducted to evaluate the efficacy of anti-inflammatory dietary interventions on mental health outcomes, and what evidence supports their effectiveness?” (Question 1).

“What do the systematic evidence syntheses available (including, SRs, SRs+MAs, UMRs, and SCR) conclude regarding the relationship between anti-inflammatory diets/dietary patterns and mental health outcomes?” (Question 2).

The citations for this scoping review were obtained from a broader evidence review focused on brain and mental health outcomes, which generated the initial search results. Screening and data extraction for the current review were performed according to the aims and eligibility criteria specific to this study.

#### 2.3.1 Addressing question 1: Identifying randomized controlled trials

All studies were evaluated according to the following inclusion criteria: (1) the publication was in English; (2) participants were adults aged 18 years or older; (3) the study included an anti-inflammatory dietary intervention; (4) the study reported a primary (e.g., depression) or secondary (e.g., comorbid heart disease and depression) mental health outcome; (5) the study was published in a peer-reviewed journal; and (6) the study used a RCT or randomized crossover design. Studies were required to meet all criteria for inclusion in this review.

#### 2.3.2 Addressing question 2: Systematic evidence synthesis

Studies were included based on the following criteria: (1) the publication was in English; (2) participants were adults aged 18 years or older; (3) the study examined the relationship between anti-inflammatory diets and mental health outcomes; (4) the study reported a primary or secondary mental health outcome^1^; (5) the study was published in a peer-reviewed journal; and (6) the study was a systematic review (SR) with or without a meta-analysis (MA), scoping review (ScR), or umbrella review (UMR). All criteria had to be met for inclusion in this review.

### 2.4 Literature search strategy

Articles were retrieved using searches conducted by a registered librarian and search specialist at the University of California, San Diego, and were uploaded into Covidence (47). Five databases were searched from their date of inception through February 2025, including PubMed, Embase, PsycINFO, CINAHL, and Web of Science. Controlled vocabulary and keyword searches were used to identify publications for inclusion. Full search strategies for each database are provided in Supplementary File S1. Reference lists of included articles were also reviewed to identify additional relevant publications that may not have been captured in the initial search or may have been excluded during screening.

### 2.5 Filtering steps for both questions

#### 2.5.1 Initial screening

All search results were imported into Covidence (48), and duplicate references were automatically removed. A total of nine trained screeners participated in the initial screening, with each title and abstract independently reviewed by two screeners. Studies that clearly did not meet the inclusion criteria based on title and abstract review were excluded. Discrepancies between screeners were resolved by one of three project leads (MLS, OL, & RM). Articles lacking abstracts or for which eligibility was unclear were automatically advanced to the secondary screening.

#### 2.5.2 Secondary screening

After the initial screening, the full text of all articles meeting the inclusion criteria was uploaded for secondary screening. Each article was reviewed by one of the three project leads and a screener. In cases of disagreement between the two reviewers, the third project lead, who had not participated in the initial review of that article, resolved the conflict. Following completion of the full-text review, the citation lists of all included studies were examined to identify any additional relevant articles.

### 2.6 Data extraction

Data were extracted independently by two screeners using standardized extraction tables in Covidence (115). Discrepancies were reviewed and resolved by a project lead. Extracted data included study design, intervention type and duration, population characteristics, outcome measures, and reported changes in outcomes along with timepoints, statistical significance for all timepoints collected (both within and between group), effect sizes, and authors conclusions specifically regarding the mental health outcome(s). For the RCTs, after data extraction, all outcomes were categorized by significance into four groups: significant, mixed which included a combination of significant and null results, null which indicated no significant findings, and negative which indicated effects in the opposite direction.

As this was a scoping review intended to map the existing literature on the impact of anti-inflammatory diets and dietary patterns on mental health outcomes, and not to evaluate efficacy, no risk-of-bias or quality assessments were conducted.

#### 2.6.1 Synthesis of results

Data were synthesized into four summary tables: (1) Two table of all included studies with details on dietary interventions and study protocols for both randomized controlled trials (RCTs) (Table 1) and systematic evidence syntheses (SRs, SRs + MAs, and UMRs) (Table 2); (2) a summary of study characteristics and results for the RCTs (Table 3); (3) a detailed table outlining specific populations, dietary interventions, and outcomes by measure for the RCTs (Supplementary Table 1); (4) a summary of study characteristics and results for the systematic evidence syntheses (Table 4).

**Table 1:** Characteristics of included randomized controlled trials.

Randomized controlled trials (*n* = 42)				
Reference	Dietary intervention	Diet intervention description	DOI #	Citation
Allison and Ditor (49)	Anti-inflammatory diet	A 12-week “anti-inflammatory diet” involving elimination of foods that provoke inflammation or intolerance and inclusion of foods and supplements with known anti-inflammatory effects.	https://doi.org/10.1186/s12974-015-0425-2	Allison DJ, Ditor DS. Targeting inflammation to influence mood following spinal cord injury: a randomized clinical trial. J Neuroinflammation. 2015 Dec;12(1):204.
Arcan et al. (50)	MedDiet plus nutrition education, text messages, and group experiential cooking	A 10-week Mediterranean diet intervention delivered via online nutrition education, text messages, group cooking sessions, and individual counseling.	https://doi.org/10.1002/osp4.725	Arcan C, Hou W, Hoffman K, Reichardt A, Yang X, Clouston SAP, et al. Mediterranean diet intervention among World Trade Center responders with post-traumatic stress disorder: Feasibility and outcomes of a pilot randomized controlled trial. Obesity Science & Practice. 2024 Feb;10(1):e725.
Aucoin et al. (51)	MedDiet	A 12-week Mediterranean-style diet (with minor modifications) delivered through biweekly dietary counseling plus omega-3 supplementation.	https://doi.org/10.1186/s40814-023-01414-y	Aucoin M, LaChance L, Van Der Wurff I, Miller S, Naidoo U, Jenkins A, et al. Dietary counseling plus omega-3 supplementation in the treatment of generalized anxiety disorder: protocol for a randomized wait-list controlled pilot trial (the “EASe-GAD Trial”). Pilot Feasibility Stud. 2023 Nov 10;9(1):186.
Bayes et al. (52)	MediDiet	A 12-week Mediterranean diet intervention emphasizing higher intake of plant-based foods, olive oil, fish, legumes, nuts, and whole grains while reducing processed foods and red meat.	https://doi.org/10.1093/ajcn/nqac106	Bayes J, Schloss J, Sibbritt D. The effect of a Mediterranean diet on the symptoms of depression in young males (the “AMMEND: A Mediterranean Diet in MEN with Depression” study): a randomized controlled trial. The American Journal of Clinical Nutrition. 2022 Aug;116(2):572–80.
Bourdel-Marchasson et al. (53)	RISTOMED diet alone	A 2-month weight-maintaining healthy dietary intervention rich in antioxidant vitamins, polyphenols, polyunsaturated fatty acids (with an n6:n3 ratio of approximately 3:1), and fiber tailored to older people and aimed at decreasing systemic inflammation, oxidative stress, and optimizing gut microbiota.	https://doi.org/10.3390/nu12030800	Bourdel-Marchasson I, Ostan R, Regueme SC, Pinto A, Pryen F, Charrouf Z, et al. Quality of Life: Psychological Symptoms—Effects of a 2-Month Healthy Diet and Nutraceutical Intervention; A Randomized, Open-Label Intervention Trial (RISTOMED). Nutrients. 2020 Mar 18;12(3):800.
	RISTOMED diet+ DeSimoneFormulation (DSF) probiotic blend	A 2-month weight-maintaining healthy dietary intervention rich in antioxidant vitamins, polyphenols, polyunsaturated fatty acids, and fiber combined with daily De Simone Formulation probiotic blend supplementation.		
	RISTOMED diet+AISA5203-L orange peel extractedmonoterpened-Limonene	A 2-month weight-maintaining healthy dietary intervention rich in antioxidant vitamins, polyphenols, polyunsaturated fatty acids, and fiber combined with daily AISA d-Limonene (orange peel extract) supplementation.		
	RISTOMED diet+Native Argan oil	A 2-month weight-maintaining healthy dietary intervention rich in antioxidant vitamins, polyphenols, polyunsaturated fatty acids, and fiber combined with a daily intake of extra-virgin Argan oil replacing an equivalent amount of dietary fat.		
Bozdoğan et al. (54)	MedDiet	A diet intervention spanning 12 weeks based on a Mediterranean dietary pattern emphasizing higher intake of plant-based foods including vegetables, fruits, whole grains, legumes, nuts, and olive oil, moderate consumption of fish and dairy, low intake of red and processed meats and refined sugars, tailored as medical nutrition therapy.	https://doi.org/10.31083/j.ceog5111237	Bozdoğan FBK, Kabaran S, Tazeoğlu A. Effect of Probiotic Supplementation on Maternal Depression, Anxiety and Attachment in Gestational Diabetes by Improving Mediterranean Diet Quality: A Randomized Controlled Trial. Clin Exp Obstet Gynecol. 2024 Nov 8;51(11):237.
	MedDiet + probiotic supplementation	A 12-week combined intervention of the same Mediterranean diet emphasizing higher intake of plant-based foods, olive oil, fish, legumes, nuts, and whole grains with reduced processed foods and red meat, plus daily probiotic supplementation containing selected live bacterial strains aimed at supporting gut microbiota.		
Cabrera-Suárez et al. (55)	MediDiet enriched w/ extra virgin olive oil	A two-year Mediterranean diet intervention enriched with extra virgin olive oil delivered remotely, emphasizing increased intake of vegetables, fruit, legumes, fish, whole grains, nuts, and generous use of extra virgin olive oil while reducing processed foods and refined cereals, combined with regular dietitian support via phone and web-based materials in addition to usual clinical care.	https://doi.org/10.1080/1028415X.2023.2283290	Cabrera-Suárez BM, Hernández-Fleta JL, Molero P, González-Pinto A, Lahortiga F, Cabrera C, et al. Mediterranean diet-based intervention to improve depressive symptoms: analysis of the PREDIDEP randomized trial. Nutritional Neuroscience. 2024 Sept;27(9):951–61.
Carcelén-Fraile et al. (56)	Mediterranean Diet	A 12-week Mediterranean diet intervention emphasizing higher intake of plant-based foods such as fruits, vegetables, legumes, nuts, olive oil, and whole grains, moderate consumption of fish and lean proteins, and lower intake of processed foods and red meat to promote overall nutrient density and support mental and physical health	https://doi.org/10.3390/nu16111741	Carcelén-Fraile MDC, Déniz-Ramírez NDP, Sabina-Campos J, Aibar-Almazán A, Rivas-Campo Y, González-Martín AM, et al. Exercise and Nutrition in the Mental Health of the Older Adult Population: A Randomized Controlled Clinical Trial. Nutrients. 2024 June 1;16(11):1741.
Casas et al. (33)	MedDiet w/ extra-virgin olive oil and walnuts	A 12-week Mediterranean diet intervention emphasizing increased intake of whole grains, vegetables, fresh fruit, legumes, nuts, fish, white meat, dairy products, and extra-virgin olive oil for cooking and dressing, with provision of extra-virgin olive oil and daily walnuts, aimed at improving dietary quality in pregnant women at high risk.	https://doi.org/10.3390/nu15102362	Casas I, Nakaki A, Pascal R, Castro-Barquero S, Youssef L, Genero M, et al. Effects of a Mediterranean Diet Intervention on Maternal Stress, Well-Being, and Sleep Quality throughout Gestation—The IMPACT-BCN Trial. Nutrients. 2023 May 18;15(10):2362.
Casini et al. (57)	MedDiet (personalized)	An 8-week personalized Mediterranean diet intervention tailored to each participant’s BMI and age emphasizing higher intake of fruits, vegetables, legumes, whole grains, fish, nuts, and extra virgin olive oil with high fiber intake, balanced macronutrient distribution, five daily meals, and exclusion of dairy products, eggs, processed foods, and red meat.	https://doi.org/10.1007/s40122-024-00598-2	Casini I, Ladisa V, Clemente L, Delussi M, Rostanzo E, Peparini S, et al. A Personalized Mediterranean Diet Improves Pain and Quality of Life in Patients with Fibromyalgia. Pain Ther. 2024 June;13(3):609–20.
Daneshzad et al. (58)	DASH Diet	A 12-week DASH diet intervention emphasizing high intake of fruits, vegetables, whole grains, low-fat dairy products, lean proteins and nuts while reducing sweets, refined grains, total fat, cholesterol, saturated fat, and sodium for women with type 2 diabetes.	https://doi.org/10.3389/fnut.2022.775543	Daneshzad E, Heshmati J, Basirat V, Keshavarz SA, Qorbani M, Larijani B, et al. The Effect of the Dietary Approaches to Stop Hypertension (DASH) Diet on Sleep, Mental Health, and Hormonal Changes: A Randomized Clinical Trial in Women With Type 2 Diabetes. Front Nutr. 2022 May 12;9:775543.
Diekmann et al. (29)	MedDiet	A single-day Mediterranean-type diet meal intervention consisting of one standardized test meal emphasizing higher intake of fruits, vegetables, whole grains, and unsaturated fats with higher fiber and micronutrient density and lower saturated fat, consumed once and followed by several hours of postprandial metabolic and inflammatory measurements.	https://doi.org/10.3390/nu11102294	Diekmann C, Wagner M, Huber H, Preuß M, Preuß P, Predel HG, et al. Acute Impact of Dietary Pattern and Walking on Postprandial Attention, Mood, and Satiety in Older Adults: A Randomized Crossover Trial. Nutrients. 2019 Sept 26;11(10):2294.
Dolatkhah et al. (59)	Anti-inflammatory diet	A 2-month anti inflammatory diet combined with a low calorie diet emphasizing high intake of fruits and vegetables, plant based proteins with added fish and low fat dairy, whole grains, olive oil as the main fat source, avoidance of trans fats, inclusion of anti inflammatory spices, and a 500 kcal per day energy deficit.	https://doi.org/10.1007/s00394-022-03017-4	Dolatkhah N, Toopchizadeh V, Barmaki S, Salekzamani Y, Najjari A, Farshbaf-Khalili A, et al. The effect of an anti-inflammatory in comparison with a low caloric diet on physical and mental health in overweight and obese women with knee osteoarthritis: a randomized clinical trial. Eur J Nutr [Internet]. 2022 Oct 2 [cited 2025 Dec 29]; Available from: https://link.springer.com/10.1007/s00394-022-03017-4
Felicetti et al., 2025 (60)	Low Fat Hypocaloric MedDiet	A 6- month hypocaloric Mediterranean diet intervention providing approximately 1,500 kcal per day for women and 1800 kcal per day for men, with a low fat macronutrient composition of about 65 percent carbohydrates, 15 percent protein, and 20 percent fat.	https://doi.org/10.1016/j.msard.2025.106271	Felicetti F, Ruggieri S, Ruotolo I, Livi C, Sellitto G, D’Ambrosi G, et al. Improvement of measured and perceived disability in overweight patients with Multiple Sclerosis trough different patterns of Mediterranean hypocaloric diet. Multiple Sclerosis and Related Disorders. 2025 Feb;94:106271.
	High Fat High Protein Hypocaloric MedDiet	A 6- month hypocaloric Mediterranean diet intervention providing approximately 1,500 kcal per day for women and 1800 kcal per day for men, with a high fat and high protein macronutrient composition of about 35 percent carbohydrates, 25 percent protein, and 40 percent fat.		
	Balanced Hypocaloric MedDiet (Guideline Based Control)	A 6 month hypocaloric Mediterranean diet intervention providing approximately 1,500 kcal per day for women and 1800 kcal per day for men, with a balanced macronutrient composition of roughly 50 to 55 percent carbohydrates, 15 to 20 percent protein, and 30 percent fat, aligned with standard Italian obesity guidelines and used as the control Mediterranean diet regimen.		
Francis et al. (61)	Australian Guide to Healthy Eating + Mediterranean-style diet recommendations	A 3-week “diet improvement” intervention based on the Australian Guide to Healthy Eating and modified toward Mediterranean-style principles (emphasizing increased intake of whole, minimally processed foods and reduced saturated fat/refined sugar).	https://doi.org/10.1371/journal.pone.0222768	Francis HM, Stevenson RJ, Chambers JR, Gupta D, Newey B, Lim CK. A brief diet intervention can reduce symptoms of depression in young adults – A randomised controlled trial. Matsuoka YJ, editor. PLoS ONE. 2019 Oct 9;14(10):e0222768.
Gazzellone et al. (20)	Mad dog diet (anti-inflammatory)	A 1 month anti-inflammatory diet intervention with personalized dietary counseling and cooking support emphasizing whole plant foods, omega 3 rich fish, healthy fats, and reduced processed foods and added sugars.	https://doi.org/10.1177/15598276231215271	Gazzellone G, Lanteigne S, Gammage K, Fajardo VA, Ditor DS. The Effects of an Anti-inflammatory Dietary Consultation on Self-efficacy, Adherence and Selected Health Outcomes: A Randomized Control Trial. American Journal of Lifestyle Medicine. 2023 Nov 15;15,598,276,231,215,271.
	Anti Inflammatory Diet Recipes	A 1 month anti inflammatory diet recipe only intervention.		
Golmohammadi et al. (63)	MIND (Mediterranean-DASH Intervention for Neurodegenerative Delay) diet	A 12-week low-calorie MIND diet (a hybrid of Mediterranean and DASH emphasizing vegetables, berries, nuts, whole grains, olive oil, fish, poultry, and minimal red meat, butter, cheese, sweets, and fried foods) with wine omitted for religious reasons.	https://doi.org/10.1038/s41598-025-91389-y	Golmohammadi M, Attari VE, Salimi Y, Saed L, Nachvak SM, Samadi M. The effect of MIND diet on sleep status, mental health, and serum level of BDNF in overweight/obese diabetic women with insomnia: a randomized controlled trial. Sci Rep. 2025 Mar 10;15(1):8237.
Hardman et al. (64)	MedDiet (plus exercise)	A 6-month Mediterranean diet intervention emphasizing higher intake of fruits, vegetables, whole grains, legumes, nuts, and extra-virgin olive oil while reducing processed foods and saturated fats.	https://doi.org/10.1093/cdn/nzaa077	Hardman RJ, Meyer D, Kennedy G, Macpherson H, Scholey AB, Pipingas A. Findings of a Pilot Study Investigating the Effects of Mediterranean Diet and Aerobic Exercise on Cognition in Cognitively Healthy Older People Living Independently within Aged-Care Facilities: The Lifestyle Intervention in Independent Living Aged Care (LIILAC) Study. Current Developments in Nutrition. 2020 May;4(5):nzaa077.
Ibarra (62)	MedDiet	A 6-month Mediterranean diet recommendation intervention encouraging regular meals with daily intake of fruits, vegetables, cereals, and nuts, fish consumption at least three times per week, and avoidance of sugary drinks.	N/A	García-Toro M. Dieta mediterránea y niveles de micronutrientes en pacientes depresivos. Nutricion Hospitalaria. 2015 Mar 1;(3):1171–5.
Jacka et al. (65)	ModiMedDiet plus nutritional consulting	A 12-week modified Mediterranean diet intervention emphasizing increased intake of whole grains, vegetables, fruits, legumes, nuts, fish, and olive oil, moderate consumption of lean meats, eggs, and dairy, and reduced intake of sweets, refined grains, fried foods, processed meats, and sugary drinks.	https://doi.org/10.1186/s12916-017-0791-y	Jacka FN, O’Neil A, Opie R, Itsiopoulos C, Cotton S, Mohebbi M, et al. A randomised controlled trial of dietary improvement for adults with major depression (the ‘SMILES’ trial). BMC Med. 2017 Dec;15(1):23.
Jeitler et al. (66)	DASH Diet With Initial Fasting and Lifestyle Modification Intervention	A 5 day water based fasting intervention followed by a 10-week DASH diet based lifestyle and dietary intervention emphasizing fruits, vegetables, whole grains, legumes, nuts, lean proteins, reduced sodium, reduced processed foods, and cardioprotective fats with Mediterranean and plant forward elements.	https://doi.org/10.3390/nu14173559	Jeitler M, Lauche R, Hohmann C, Choi KE (Anna), Schneider N, Steckhan N, et al. A Randomized Controlled Trial of Fasting and Lifestyle Modification in Patients with Metabolic Syndrome: Effects on Patient-Reported Outcomes. Nutrients. 2022 Aug 29;14(17):3559.
	DASH Diet Lifestyle Modification Only Intervention	A 10-week DASH diet based lifestyle and dietary intervention emphasizing fruits, vegetables, whole grains, legumes, nuts, lean proteins, reduced sodium, reduced processed foods, and cardioprotective fats with Mediterranean and plant elements.		
Kabiri et al. (67)	Mediterranean DASH Intervention for Neurodegenerative Delay (MIND)	A 12-week low calorie Mediterranean DASH Intervention for Neurodegenerative Delay diet emphasizing higher intake of green leafy vegetables, other vegetables, berries, nuts, whole grains, legumes, fish, olive oil and poultry while limiting butter, cheese, red meat, pastries, sweets and fried or fast foods with an overall calorie restriction.	https://doi.org/10.1017/S0007114524001168	Kabiri SS, Javanbakht Z, Zangeneh M, Moludi J, Saber A, Salimi Y, et al. The effects of MIND diet on depression, anxiety, quality of life and metabolic and hormonal status in obese or overweight women with polycystic ovary syndrome: a randomised clinical trial. Br J Nutr. 2024 Oct 28;1–14.
	Low Calorie Diet	A 12-week low calorie control diet emphasizing general calorie restriction based on standard healthy eating recommendations without specific emphasis on Mediterranean DASH or MIND diet food groups, designed to achieve a comparable energy deficit.		
Lee et al. (68)	MedDiet	A 10-day Mediterranean-style diet emphasizing vegetables, fruits, whole grains, legumes, nuts, fish, lean meats, olive oil, and minimal intake of red meat, butter, cheese, sweets, and fried foods.	https://doi.org/10.1016/j.nut.2014.10.008	Lee J, Pase M, Pipingas A, Raubenheimer J, Thurgood M, Villalon L, et al. Switching to a 10-day Mediterranean-style diet improves mood and cardiovascular function in a controlled crossover study. Nutrition. 2015 May;31(5):647–52.
Long Parma et al. (69)	Anti-inflammatory diet	A 6-month anti-inflammatory dietary intervention emphasizing higher intake of fruits, vegetables, whole grains, legumes, nuts, olive oil, herbs and spices, and lean proteins such as fish while reducing processed foods, red meat, and pro-inflammatory foods.	https://doi.org/10.1007/s00520-022-07023-4	Long Parma DA, Reynolds GL, Muñoz E, Ramirez AG. Effect of an anti-inflammatory dietary intervention on quality of life among breast cancer survivors. Support Care Cancer. 2022 July;30(7):5903–10.
McMillan et al. (70)	MediDiet	A 10-day Mediterranean diet intervention emphasizing higher intake of fish, a variety of fruits and vegetables, olive oil, whole grains, and legumes while reducing refined carbohydrates and saturated fats for the diet change group to assess effects on mood and cognitive performance.	https://doi.org/10.1016/j.appet.2010.11.149	McMillan L, Owen L, Kras M, Scholey A. Behavioral effects of a 10-day Mediterranean diet. Results from a pilot study evaluating mood and cognitive performance. Appetite. 2011 Feb;56(1):143–7.
Miralles-Amorós et al. (71)	MediDiet	A 12-week Mediterranean diet intervention emphasizing higher intake of whole grains, vegetables, fruits, legumes, fish, white meat, and olive oil while meeting recommended micronutrient and antioxidant intakes	https://doi.org/10.3390/children10020259	Miralles-Amorós L, Vicente-Martínez M, Martínez-Olcina M, Asencio-Mas N, Gonzálvez-Alvarado L, Peñaranda-Moraga M, et al. Study of Different Personalised Dietary Plans on Eating Behavior, Body Image and Mood in Young Female Professional Handball Players: A Randomised Controlled Trial. Children. 2023 Jan 31;10(2):259.
	High Antioxidant Diet	A 12-week high antioxidant diet intervention emphasizing increased consumption of antioxidant rich fruits and vegetables such as blueberries, beetroot, and pomegranate to exceed standard recommendations for antioxidant vitamins		
O’Connor et al. (72)	MediDiet High Red Meat Diet	A 5 week Mediterranean diet intervention emphasizing plant based foods, olive oil, fish, legumes, nuts, and whole grains while including approximately 500 g per week of lean unprocessed red meat with remaining protein from poultry and dairy.	https://doi.org/10.1093/jn/nxy235	O’Connor LE, Biberstine SL, Paddon-Jones D, Schwichtenberg AJ, Campbell WW. Adopting a Mediterranean-Style Eating Pattern with Different Amounts of Lean Unprocessed Red Meat Does Not Influence Short-Term Subjective Personal Well-Being in Adults with Overweight or Obesity. The Journal of Nutrition. 2018 Dec;148(12):1917–23.
	MediDiet Low Red Meat Diet	A 5 week Mediterranean diet intervention emphasizing plant based foods, olive oil, fish, legumes, nuts, and whole grains while including approximately 200 g per week of lean unprocessed red meat with remaining protein from poultry and dairy.		
Papandreou et al. (73)	MediDiet	A 12-week personalized Mediterranean diet intervention delivered through a clinical decision support system emphasizing higher intake of fruits, vegetables, whole grains, legumes, fish, nuts, and olive oil as the main fat source with tailored nutritional guidance based on individual needs and preferences and standard “National Dietary Guidelines for Pregnancy” recommendations integrated into the plan.	https://doi.org/10.3390/nu15020432	Papandreou P, Amerikanou C, Vezou C, Gioxari A, Kaliora AC, Skouroliakou M. Improving Adherence to the Mediterranean Diet in Early Pregnancy Using a Clinical Decision Support System; A Randomised Controlled Clinical Trial. Nutrients. 2023 Jan 14;15(2):432.
Parletta et al. (74)	MediDiet	A 12-week Mediterranean-style diet intervention emphasizing higher intake of vegetables, fruit, nuts, legumes and whole grains, practical support with food hampers and cooking workshops to increase adherence to Mediterranean dietary patterns, combined with daily fish oil supplementation for a total of 6 months to increase omega-3 intake and reduce unhealthy snacks and red meat consumption.	https://doi.org/10.1080/1028415X.2017.1411320	Parletta N, Zarnowiecki D, Cho J, Wilson A, Bogomolova S, Villani A, et al. A Mediterranean-style dietary intervention supplemented with fish oil improves diet quality and mental health in people with depression: A randomized controlled trial (HELFIMED). Nutritional Neuroscience. 2019 July 3;22(7):474–87.
Radkhah et al. (75)	MediDiet	A 12-week Mediterranean diet intervention emphasizing higher intake of plant-based foods such as vegetables, fruits, seeds, legumes, nuts, and whole grains; use of extra virgin olive oil as the primary fat source; moderate consumption of fish, dairy, and poultry; limited intake of red and processed meats, eggs, sweeteners, and highly processed foods.	https://doi.org/10.1016/j.pmedr.2023.102469	Radkhah N, Rasouli A, Majnouni A, Eskandari E, Parastouei K. The effect of Mediterranean diet instructions on depression, anxiety, stress, and anthropometric indices: A randomized, double-blind, controlled clinical trial. Preventive Medicine Reports. 2023 Dec;36:102469.
Rumbo-Rodríguezet al (76).	MediDiet	A two-year Mediterranean diet intervention emphasizing higher intake of fruits, vegetables, whole grains, legumes, fish, and healthy fats (such as olive oil), with personalized menus and education to improve balanced eating habits while reducing intake of refined and processed foods, as part of a comprehensive weight management and lifestyle program.	https://doi.org/10.3390/nu14224762	Rumbo-Rodríguez L, Zaragoza-Martí A, Sánchez-SanSegundo M, Ferrer-Cascales R, Laguna-Pérez A, Hurtado-Sánchez JA. Effectiveness of a Two-Year Multicomponent Intervention for the Treatment of Overweight and Obesity in Older People. Nutrients. 2022 Nov 11;14(22):4762.
Sabet et al. (26)	Healthy Nordic Diet (ND)	An 8-day Healthy Nordic Diet intervention emphasizing high intake of vegetables, legumes, fruits, fish, and whole grains (mostly non-wheat such as barley, oats, rye), with no refined grains, added sugars, red meat, or processed meat, using low-fat dairy and plant-based oils (primarily rapeseed oil), designed to meet or exceed Nordic Nutrition Recommendations, provided as all meals and snacks for participants.	https://doi.org/10.3390/nu13030902	Sabet JA, Ekman MS, Lundvall AS, Risérus U, Johansson U, Öström Å, et al. Feasibility and Acceptability of a Healthy Nordic Diet Intervention for the Treatment of Depression: A Randomized Controlled Pilot Trial. Nutrients. 2021 Mar 10;13(3):902.
Sánchez-Villegas et al. (77)	MediDiet supplemented with extra virgin olive oil (MD-EVOO)	A ≥ 3-year Mediterranean diet intervention supplemented with extra-virgin olive oil, emphasizing higher intake of plant-based foods, fruits, vegetables, legumes, fish, nuts, and whole grains; abundant use of olive oil for cooking and dressing; moderate wine consumption if appropriate; and reduced intake of red and processed meats, butter, sweets, pastries, fast food, and sugar-sweetened beverages, with participants receiving approximately 1 liter of extra-virgin olive oil per week.	https://doi.org/10.1186/1741-7015-11-208	Sánchez-Villegas A, Martínez-González MA, Estruch R, Salas-Salvadó J, Corella D, Covas MI, et al. Mediterranean dietary pattern and depression: the PREDIMED randomized trial. BMC Med. 2013 Dec;11(1):208.
	MediDiet supplemented with mixed nuts (MD-nuts)	A ≥ 3-year Mediterranean diet intervention supplemented with mixed nuts, emphasizing higher intake of plant-based foods, fruits, vegetables, legumes, fish, nuts, and whole grains; moderate wine consumption if appropriate; and reduced intake of red and processed meats, butter, sweets, pastries, fast food, and sugar-sweetened beverages, with participants receiving 30 g/day of mixed nuts (15 g walnuts, 7.5 g hazelnuts, and 7.5 g almonds).		
Staudacher et al. (31)	MediDiet	A 6-week personalized Mediterranean diet intervention that emphasized a traditional Mediterranean dietary pattern with higher intake of plant-based foods (vegetables, fruits, legumes, whole grains, nuts), olive oil as the primary source of fat, moderate fish consumption, and reduced intake of processed foods and red meat. Participants received personalized dietary counseling from a dietitian and ongoing support to increase adherence to Mediterranean diet principles.	https://doi.org/10.1111/apt.17791	Staudacher HM, Mahoney S, Canale K, Opie RS, Loughman A, So D, et al. Clinical trial: A Mediterranean diet is feasible and improves gastrointestinal and psychological symptoms in irritable bowel syndrome. Aliment Pharmacol Ther. 2024 Feb;59(4):492–503.
Toobert et al. (78)	MediDiet	A 2-year Mediterranean diet intervention emphasizing higher intake of bread, root and green vegetables, legumes, daily fruit, fish, and monounsaturated fat sources such as olive or canola oil, while reducing saturated fat, butter, cream, and red meat, and replacing red meat with poultry; individualized carbohydrate and fat recommendations were provided to optimize blood glucose and lipid levels, with ongoing self-monitoring and group-based dietary support.	https://doi.org/10.1186/1479-5868-4-1	Toobert DJ, Glasgow RE, Strycker LA, Barrera M, Ritzwoller DP, Weidner G. Long-term effects of the Mediterranean lifestyle program: a randomized clinical trial for postmenopausal women with type 2 diabetes. Int J Behav Nutr Phys Act. 2007 Dec;4(1):1.
Torres et al. (79)	LNAHK Diet	A 4-week low-sodium, high-potassium (LNAHK) diet intervention emphasizing substantial reduction in sodium intake while increasing potassium intake through higher consumption of fruits, vegetables, and other potassium-rich foods, with lower calcium intake compared to DASH-type patterns.	https://doi.org/10.1017/S0007114508959201	Torres SJ, Nowson CA, Worsley A. Dietary electrolytes are related to mood. Br J Nutr. 2008 Nov;100(5):1038–45.
	High-Calcium (HC) Diet	A 4-week high-calcium (HC) diet intervention emphasizing increased calcium intake, primarily through calcium-rich foods such as dairy products, while maintaining moderate sodium and potassium intake.		
	Dietary Approaches to Stop Hypertension (DASH)-type diet	A 4-week DASH-type (OD) diet intervention emphasizing a moderate-sodium, high-potassium, high-calcium dietary pattern rich in fruits, vegetables, whole grains, low-fat dairy products, and fiber, while reducing saturated fat intake.		
Torres and Nowson (18)	Moderate sodium DASH-type diet	A 14-week moderate-sodium DASH-type diet intervention emphasizing higher intake of fruits and vegetables (at least four servings each per day), increased dietary potassium and magnesium, moderate sodium intake (~70 mmol/day), inclusion of lean red meat (six servings per week), limited breads and cereals (no more than four servings per day), and low saturated fat consumption.	https://doi.org/10.1016/j.nut.2011.11.029	Torres SJ, Nowson CA. A moderate-sodium DASH-type diet improves mood in postmenopausal women. Nutrition. 2012 Sept;28(9):896–900.
Wade et al. (80)	MediDiet with additional red meat (MedPork)	An 8-week Mediterranean diet intervention inclusive of 2–3 weekly servings of fresh, lean pork, emphasizing higher intake of extra virgin olive oil, vegetables, fruits, legumes, nuts, seafood, and whole grains alongside lean pork while reducing refined grains and traditional high-fat foods compared with a low-fat diet.	https://doi.org/10.3390/nu11071521	Wade AT, Davis CR, Dyer KA, Hodgson JM, Woodman RJ, Keage HAD, et al. A Mediterranean Diet with Fresh, Lean Pork Improves Processing Speed and Mood: Cognitive Findings from the MedPork Randomised Controlled Trial. Nutrients. 2019 July 4;11(7):1521.
Wade et al. (81)	MediDiet supplemented with diary foods (MedDairy)	An 8-week Mediterranean diet supplemented with dairy (MedDairy) intervention emphasizing higher intake of vegetables, fruits, legumes, nuts, whole grains, fish, and extra virgin olive oil, with moderate consumption of poultry, eggs, and red wine, while limiting red and processed meats, sweets, and refined foods, and specifically including 3–4 daily servings of dairy foods to achieve adequate calcium intake.	https://doi.org/10.1080/1028415X.2018.1543148	Wade AT, Davis CR, Dyer KA, Hodgson JM, Woodman RJ, Keage HAD, et al. A Mediterranean diet supplemented with dairy foods improves mood and processing speed in an Australian sample: results from the MedDairy randomized controlled trial. Nutritional Neuroscience. 2020 Aug 2;23(8):646–58.
Wardle et al. (82)	MedDiet	A 12-week Mediterranean dietary intervention adapted for cholesterol lowering; participants were instructed to follow a Mediterranean-style diet pattern that included higher consumption of plant-based foods (vegetables, legumes, fruits, whole grains), olive oil and other sources of unsaturated fats (e.g., nuts, seeds), moderate fish intake, and reduced intake of processed foods and red meats.	https://doi.org/10.1016/S0002-9343(00)00330-2	Wardle J, Rogers P, Judd P, Taylor MA, Rapoport L, Green M, et al. Randomized trial of the effects of cholesterol-lowering dietary treatment on psychological function. The American Journal of Medicine. 2000 May;108(7):547–53.
Wright et al. (83)	DASH diet with mindfulness	An 8-week DASH diet intervention emphasizing reduced sodium intake and limiting sweets, sugary beverages, and red meats, while increasing consumption of foods rich in potassium, calcium, magnesium, fiber, and protein (e.g., fruits, vegetables, whole grains, and low-fat foods), including culturally adapted “Soul food” recipes aligned with DASH principles and practical skill-building through goal setting, problem solving, and food sampling.	https://doi.org/10.1111/jgs.16947	Wright KD, Klatt MD, Adams IR, Nguyen CM, Mion LC, Tan A, et al. Mindfulness in Motion and Dietary Approaches to Stop Hypertension (DASH) in Hypertensive African Americans. J American Geriatrics Society. 2021 Mar;69(3):773–8.
Yau et al. (84)	Mediterranean-DASH intervention for neurodegenerative delay (MIND) diet with nutritional counseling	A 4-week MIND diet intervention emphasizing increased intake of brain-healthy foods such as green leafy vegetables, other vegetables, berries, nuts, beans, whole grains, seafood, poultry, olive oil, and moderate wine while limiting intake of red meat, butter and margarine, cheese, pastries and sweets, and fried/fast foods, alongside four nutrition education sessions.	https://doi.org/10.3390/ijerph192214665	Yau KY, Law PS, Wong CN. Cardiac and Mental Benefits of Mediterranean-DASH Intervention for Neurodegenerative Delay (MIND) Diet plus Forest Bathing (FB) versus MIND Diet among Older Chinese Adults: A Randomized Controlled Pilot Study. IJERPH. 2022 Nov 8;19(22):14665.
	Mediterranean-DASH intervention for neurodegenerative delay (MIND) diet with nutritional counseling and forest bathing	A 4-week MIND diet plus Forest Bathing (FB) intervention combining the same dietary pattern as above with structured low-intensity forest bathing sessions on four consecutive weekends for additional relaxation and mental health benefit.		

**Table 2:** Characteristics of systematic evidence syntheses.

Systematic evidence syntheses (*n* = 23)						
Reference	Study design	Review aim	Eligibility criteria	Anti-inflammatory diets/dietary patterns assessed	DOI #	Citation
Altun et al. (104)	SR	Investigate the relationship between depression and the Mediterranean diet, and to identify future interventions that may facilitate the treatment and management of depressive symptoms.	A pattern high in plant based foods such as vegetables and fruits, along with foods like legumes, nuts, cereals, olive oil, and fish, and low in meat and dairy. For inclusion, studies had to include high plant food intake plus at least one core Mediterranean component such as olive oil, fish, nuts, legumes, or cereals	MedDiet (*n* = 26)	https://doi.org/10.1016/j.npbr.2019.05.007	Altun A, Brown H, Szoeke C, Goodwill AM. The Mediterranean dietary pattern and depression risk: A systematic review. Neurology, Psychiatry and Brain Research. 2019 Sept;33:1–10.
Bizzozero-Peroni et al. (91)	SR + MA	Synthesize findings on the effects of Mediterranean Diet interventions on the severity of depressive symptoms in adults with depression.	Mediterranean diet (MD)includes consumption of antioxidant-rich food groups, such as fish, fruits, nuts, pulses, seeds, and vegetables.	MedDiet (*n* = 5)	https://doi.org/10.1093/nutrit/nuad176	Bizzozero-Peroni B, Martínez-Vizcaíno V, Fernández-Rodríguez R, Jiménez-López E, Núñez De Arenas-Arroyo S, Saz-Lara A, et al. The impact of the Mediterranean diet on alleviating depressive symptoms in adults: a systematic review and meta-analysis of randomized controlled trials. Nutrition Reviews. 2025 Jan 1;83(1):29–39.
DeSmet et al., 2024 (92)	SR/MA	Summarize experimental evidence of (group-) randomized controlled trials on the effectiveness of dietary interventions on mental well-being in a general population of emerging adults (18–29y).	Dietary interventions were included if they directly targeted food consumption that participants could realistically adopt in daily life. Interventions involved specific foods, food groups, or whole dietary patterns and were delivered in either laboratory or natural settings.	MedDiet (*n* = 2)	https://doi.org/10.1007/s10902-024-00779-y	DeSmet A, Culot C, Virani D, Pabian S, Ariño Mateo E, Van Royen K, et al. A Systematic Review and Meta-analysis of the Effectiveness of Dietary Interventions to Enhance Mental Wellbeing in Emerging Adulthood. J Happiness Stud. 2024 Aug;25(6):68.
Dinu et al. (101)	UMR	Summarize the available evidence on the existing meta-analyses on the Mediterranean diet and different health outcomes, and provide an overview of the validity of the studied associations, by evaluating also possible hints of biases.	A pattern based on the traditional foods and drinks of countries surrounding the Mediterranean Sea. It emphasizes a high intake of plant-based foods, with particular importance placed on foods such as fruits, vegetables, legumes, cereals, and olive oil, alongside fish consumption, and lower intakes of meat and dairy.	MedDiet (*n* = 25)	https://doi.org/10.1038/ejcn.2017.58	Dinu M, Pagliai G, Casini A, Sofi F. Mediterranean diet and multiple health outcomes: an umbrella review of meta-analyses of observational studies and randomised trials. Eur J Clin Nutr. 2018 Jan;72(1):30–43.
Eliby et al. (105)	SR	Investigate differences in dietary quality between healthy individuals and those with current anxiety and/or depressive disorders (including symptom severity). Investigate the prospective associations between diet quality and the risk for anxiety and/or depressive disorders (including symptom severity).	The review analyzed studies that assessed the relationship between overall diet quality and mental health outcomes, focusing on whole-of-diet patterns rather than individual nutrients or foods.	MedDiet Score (MDS) or its variants (*n* = 14); Dietary Inflammatory Index (DII) (*n* = 3); Dietary Approaches to Stop Hypertension (DASH) index (*n* = 2); Mediterranean-DASH diet Intervention for Neurodegenerative Delay (*n* = 1); Inflammatory Dietary Pattern (IDP) (*n* = 1)	https://doi.org/10.1016/j.jadr.2023.100629	Eliby D, Simpson CA, Lawrence AS, Schwartz OS, Haslam N, Simmons JG. Associations between diet quality and anxiety and depressive disorders: A systematic review. Journal of Affective Disorders Reports. 2023 Dec;14:100629.
Gianfredi et al. (102)	UMR	Assess the strength and validity of the available observational and trial evidence for the association between a variety of dietary patterns and depression.	Adherence to any dietary patterns/dietary interventions were eligible. The focus of this review was to assess the available evidence for an association between depression and whole-of-diet, rather than individual foods or nutrients.	MedDiet (*n* = 6); Dietary Inflammatory Index (DII) (*n* = 5); Dietary Approaches to Stop Hypertension (DASH) (*n* = 2)	https://doi.org/10.1093/nutrit/nuac058	Gianfredi V, Dinu M, Nucci D, Eussen SJPM, Amerio A, Schram MT, et al. Association between dietary patterns and depression: an umbrella review of meta-analyses of observational studies and intervention trials. Nutrition Reviews. 2023 Feb 10;81(3):346–59.
Jafari and Behrouz (106)	SR	Determine how ND affects neurological function. Provide a summary of the primary molecular mechanisms that are proposed to explain the relationship between the ND, its components, neurological function, and brain health.	The Nordic Diet (DT) is a dietary pattern focusing on the consumption of traditional, organic, plant-based, and local foods originating from the Scandinavian countries including Iceland, Finland, Norway, Sweden, and Denmark. Generally, this dietary pattern includes less fat, less sugar, less alcohol, high fiber, and high in fish and seafood.	Nordic Diet (*n* = 8)	https://doi.org/10.3389/fnut.2023.1215358	Jafari RS, Behrouz V. Nordic diet and its benefits in neurological function: a systematic review of observational and intervention studies. Front Nutr. 2023 Aug 14;10:1215358.
Lassale et al. (93)	SR/MA	Synthesize the link between diet quality, measured using a range of predefined indices, and depressive outcomes.	The review analyzed various dietary indices, including the Mediterranean diet, Healthy Eating Index (HEI), Alternative Healthy Eating Index (AHEI), Dietary Inflammatory Index (DII), and adherence to the Dietary Approaches to Stop Hypertension (DASH) diet, to assess their association with depressive outcomes.	MedDiet (*n* = 10); Dietary Inflammatory Index (DII) (*n* = 9); DASH Diet (*n* = 4)	https://doi.org/10.1038/s41380-018-0237-8	Lassale C, Batty GD, Baghdadli A, Jacka F, Sánchez-Villegas A, Kivimäki M, et al. Healthy dietary indices and risk of depressive outcomes: a systematic review and meta-analysis of observational studies. Mol Psychiatry. 2019 July;24(7):965–86.
Madani et al. (107)	SR	Evaluate the relationship between the Mediterranean diet and Axis I disorders.	The review focuses on the Mediterranean diet, which emphasizes the consumption of fruits, vegetables, whole grains, legumes, nuts, olive oil, and fish, while limiting the intake of red meat and sweets.	MedDiet (*n* = 36)	https://doi.org/10.1002/fsn3.2950	Madani S, Ahmadi A, Shoaei-Jouneghani F, Moazen M, Sasani N. The relationship between the Mediterranean diet and Axis I disorders: A systematic review of observational studies. Food Science & Nutrition. 2022 Oct;10(10):3241–58.
Matison et al. (50)	SR/MA	Systematically examine the longitudinal observational evidence between diet and the incidence of depression in adults aged 45 years and older.	Study included “Any form of nutrition intake (e.g., food patterns, food groups, foods, micronutrients, or dietary supplements)”; For our purposes, relevant interventions are: Mediterranean Diet, the original Dietary Inflammatory Index (DII), or the Alternate DII, to assess the relationship between the level of inflammation in the diet and risk of incident depression.	MedDiet (*n* = 3); Dietary Inflammatory Index DII (*n* = 2); Alternate Dietary Inflammatory Index ADII (*n* = 1)	https://doi.org/10.1016/j.arr.2021.101403	Matison AP, Mather KA, Flood VM, Reppermund S. Associations between nutrition and the incidence of depression in middle-aged and older adults: A systematic review and meta-analysis of prospective observational population-based studies. Aging Research Reviews. 2021 Sept;70:101403.
Molendijk et al. (95)	SR/MA	Investigate the evidence for the claim that the quality of a diet is associated with the incidence of depressive disorders.	Included studies reported on the association between dietary patterns or the consumption of food groups and the incidence of depression (high-quality/healthy unhealthy, or neutral dietary patterns).	MedDiet (*n* = 5)	https://doi.org/10.1016/j.jad.2017.09.022	Molendijk M, Molero P, Ortuño Sánchez-Pedreño F, Van Der Does W, Angel Martínez-González M. Diet quality and depression risk: A systematic review and dose–response meta-analysis of prospective studies. Journal of Affective Disorders. 2018 Jan;226:346–54.
O’Neill et al. (86)	SR	Understand the effectiveness of whole food or whole diet dietary interventions to support the treatment of depression in healthy adults.	The review included whole food or whole diet interventions. A whole food intervention involved the consumption of a minimally processed, unfortified food item that is easily accessible in the Western food chain, for example, orange juice. A whole diet intervention adjusted or monitored the entire dietary intake.	MedDiet (*n* = 2)	https://doi.org/10.3390/nu14071398	O’Neill S, Minehan M, Knight-Agarwal CR, Turner M. Depression, Is It Treatable in Adults Utilizing Dietary Interventions? A Systematic Review of Randomised Controlled Trials. Nutrients. 2022 Mar 27;14(7):1398.
Psaltopoulou et al. (96)	SR/MA	Quantitatively synthesize all studies that examine the association between adherence to a Mediterranean diet and risk of stroke, depression, cognitive impairment, and Parkinson disease	MedDiet is characterized by high intake of vegetables, fruits, cereals, pulses, nuts, and seeds; moderate consumption of dairy products, fish, poultry, eggs, and unsaturated fats, such as olive oil; low to moderate intake of wine during meals; and low intake of red, processed meats and saturated fats.	MedDiet (*n* = 22)	https://doi.org/10.1002/ana.23944	Psaltopoulou T, Sergentanis TN, Panagiotakos DB, Sergentanis IN, Kosti R, Scarmeas N. Mediterranean diet, stroke, cognitive impairment, and depression: A meta-analysis. Annals of Neurology. 2013 Oct;74(4):580–91.
Rudzińska et al. (87)	SR	Assess whether the degree of adherence to the Mediterranean diet is related to the occurrence and severity of depressive symptoms in people over 65 years of age.	The Mediterranean diet is characterized by a high supply of low-processed foods, such as fresh vegetables and fruits, grains, nuts, seeds and legumes, and a moderate supply of animal products: meat, eggs, and dairy. In this model of nutrition, fish is eaten twice a week, and olive oil is the main source of fat in the diet.	MedDiet (*n* = 9)	https://doi.org/10.12740/PP/OnlineFirst/140465	Rudzińska A, Perera I, Gryglewska B, Gąsowski J, Piotrowicz K. Can the Mediterranean diet decrease the risk of depression in older persons – a systematic review. Psychiatr Pol. 2023 Apr 30;57(2):339–54.
Selvaraj et al. (85)	SR	Analyze the association between dietary habits and depression in adolescents, adults, and older people.	Included studies talk about regular eating patterns/intake, as well as the type and quality of food.	MedDiet (*n* = 3); Nordic Diet (*n* = 1); DASH Diet (*n* = 1)	https://doi.org/10.7759/cureus.32359	Selvaraj R, Selvamani TY, Zahra A, Malla J, Dhanoa RK, Venugopal S, et al. Association Between Dietary Habits and Depression: A Systematic Review. Cureus [Internet]. 2022 Dec 9 [cited 2026 Jan 3]; Available from: https://www.cureus.com/articles/91449-association-between-dietary-habits-and-depression-a-systematic-review
Shafiei et al. (97)	SR/MA	Summarize earlier evidence on the link between adherence to the Mediterranean diet and the risk of depression.	The Mediterranean diet is a healthy diet in which consumption of vegetables and fruits, nuts and seeds, whole grains, dairy products, olive oil, fish, and fresh seafood is encouraged, while consumption of red and processed meats is restricted.	MedDiet (*n* = 19)	https://doi.org/10.1093/nutrit/nuy070	Shafiei F, Salari-Moghaddam A, Larijani B, Esmaillzadeh A. Adherence to the Mediterranean diet and risk of depression: a systematic review and updated meta-analysis of observational studies. Nutrition Reviews. 2019 Apr 1;77(4):230–9.
Solomou et al. (88)	SR	Examine bidirectional associations from observational studies regarding the influence of diet quality on the mental health of university students, and vice versa.	The review included whole of diet interventions rather than individual foods/nutrients.	DASH (*n* = 2); Energy-adjusted Dietary Inflammatory Index (*n* = 2); Dietary Inflammatory Index (*n* = 1); MedDiet (*n* = 5)	https://doi.org/10.1093/her/cyac035	Solomou S, Logue J, Reilly S, Perez-Algorta G. A systematic review of the association of diet quality with the mental health of university students: implications in health education practice. Health Education Research. 2023 Jan 20;38(1):28–68.
Suschana et al. (89)	SR	Investigate the role of anti-inflammatory diets and vitamin supplementation in the management of metabolic syndrome and in symptom remission in people with schizophrenia.	The review included interventions focused on anti-inflammatory diets or vitamins.	DASH diet or structured anti inflammatory dietary advice (*n* = 1); Fatty acid and omega 3 based supplementation (*n* = 6); Vitamin D supplementation alone or in combination (*n* = 4); B vitamin supplementation including folate and vitamin B6 (*n* = 3); Mineral supplementation such as magnesium (*n* = 1); Probiotic or gut focused nutritional intervention (*n* = 1); Other nutrition related adjunct interventions (*n* = 1).	https://doi.org/10.3389/fpsyt.2024.1506353	Suschana E, Anderson T, Hong C, Narikatte A, Silverberg J, Sharma MS. The role of anti-inflammatory diets and supplementation in metabolic syndrome and symptom remission in adults with schizophrenia: a systematic review. Front Psychiatry. 2025 Jan 7;15:1506353.
Tan et al. (90)	SR	Examine the current literature on the association between the DASH diet and mental health.	The Dietary Approaches to Stop Hypertension (DASH) diet is a dietary pattern promoted to prevent and control hypertension. It focuses on 8 dietary components: a high intake of fruits, vegetables, low-fat dairy, nuts and legumes, and whole grains, as well as a low intake of sodium, sweetened beverages, and red and processed meats.	DASH Diet (*n* = 16)	https://doi.org/10.1093/nutrit/nuad038	Tan J, Wang C, Tomiyama AJ. Dietary Approaches to Stop Hypertension (DASH) diet and mental well-being: a systematic review. Nutrition Reviews. 2023 Dec 11;82(1):60–75.
Tavakoly et al. (98)	SR/MA	Investigate the effects of different dietary patterns on depressive disorders.	Based on the Dietary Guidelines Advisory Committee 2020. The following dietary patterns were identified: South Beach, Atkins, ketogenic, gluten free, prudent, Nordic, paleolithic, plant based, Mediterranean, vegetarian, macrobiotic, vegan, DASH, immunonutrition, fermentable oligosaccharides disaccharides monosaccharides and polyols (FODMAP), low sodium, pescetarian, high fiber, whole grain, traditional, polyphenol, anti-oxidant, anti-inflammatory, fruit and vegetable, flexitarian, and fruitarian.	MedDiet (*n* = 5)	https://doi.org/10.3390/nu17030563	Tavakoly R, Moosburner A, Anheyer D, Cramer H. Effect of Different Dietary Patterns on Patients with Depressive Disorders: A Systematic Review and Meta-Analysis of Randomized Controlled Trials. Nutrients. 2025 Jan 31;17(3):563.
Tolkien et al. (99)	SR/MA	Examine current literature regarding depression and the inflammatory potential of the diet to determine if diet could be an effective treatment for depression.	Included studies measured the inflammatory potential of the diet [CRP, IL-6, TNF-α receptor 2, DII (Dietary Inflammatory Index) score]	Anti-inflammatory diet (*n* = 11)	https://doi.org/10.1016/j.clnu.2018.11.007	Tolkien K, Bradburn S, Murgatroyd C. An anti-inflammatory diet as a potential intervention for depressive disorders: A systematic review and meta-analysis. Clinical Nutrition. 2019 Oct;38(5):2045–52.
Wang et al. (100)	SR/MA	Evaluate the association between the inflammatory potential of diet, determined by the dietary inflammatory index (DII®) score, and depression.	Included studies measured the inflammatory potential of the diet estimated by DII score as the exposure.	Anti-inflammatory diet (*n* = 6)	https://doi.org/10.1017/S1368980018002628	Wang J, Zhou Y, Chen K, Jing Y, He J, Sun H, et al. Dietary inflammatory index and depression: a meta-analysis. Public Health Nutr. 2019 Mar;22(4):654–60.
Zupo et al. (103)	UMR	Assess and weigh the relationship between adherence to the MedDiet pattern and human health benefits.	The MedDiet model, described as mainly based on the consumption of plant-based foods, such as fruits and vegetables, and the supplementation with whole grains, beans, nuts, seafood, lean poultry, and unsaturated fats from extra virgin olive oil.	MedDiet (*n* = 84)	https://doi.org/10.1186/s12967-023-04618-1	Zupo R, Castellana F, Piscitelli P, Crupi P, Desantis A, Greco E, et al. Scientific evidence supporting the newly developed one-health labeling tool “Med-Index”: an umbrella systematic review on health benefits of mediterranean diet principles and adherence in a planeterranean perspective. J Transl Med. 2023 Oct 26;21(1):755.

SR- Systematic review; SR+MA- Systematic Review with a meta-analysis; UMR- Umbrella review.

**Table 3:** Overview of randomized controlled trials investigating dietary interventions for mental health.

Citation	Design	Intervention	Study duration	Control/Comparison	Population	Outcome	*p*-value	Effect size	Author’s conclusion
Allison and Ditor (49)	RCT	Anti-Inflamatory Diet	12 weeks (assessments at baseline, 1 mos, and 3 moths)	Inactive Control	Participants were recovering from spinal cord injury (*n* = 20)	Depression (CES-D)			Overall, the results demonstrated the effectiveness of targeting inflammation as a means of improving mood in SCI, with potential mechanisms relating to the reduction in IL-1βand improvements in levels of neuroactive compounds related to the kynurenine pathway. Due to the limited sample size, results should be interpreted with caution; however, they are worthy of further examination due to the potential impact of inflammation on depression.
						Group x time	*p* = 0.01	d = 1.07	
						Treatment Group:			
						Baseline - 1 month	*p* = 0.01	NR	
						Baseline - 3 months	*p* < 0.01	NR	
						Control Group:			
						Baseline - 1 month	*p* = 0.03	NR	
						Baseline - 3 months	NS	NR	
Arcan et al. (50)	RCT	MedDiet	10-week intervention with 3-month post-intervention follow-up (total duration ≈ 6 months).	Control	WTC Health Program members (*N* = 62), 45–65 years, males 87%, body mass index (BMI) 27–45 kg/m2	PTSD (PCL-5)	*p* < 0.0001	NR	When accompanied by regular contact and individual goal setting, as in this study, nutritional interventions can potentially improve weight and mental health in participants with comorbid overweight and obesity and mental health conditions. In summary, the study successfully met its aims and provided valuable lessons that can be used to guide the development of a full-scale trial, while components of the intervention can be implemented in clinics serving populations with comorbid physical and mental health issues.
Aucoin et al. (51)	RCT	MedDiet	12 weeks	Control	50 adult women with generalized anxiety disorder.	Anxiety (BAI)	*p* < 0.0001	NR	The EASe-GAD pilot study was found to be highly feasible and acceptable. Participation in the intervention was associated with an improvement in anxiety symptom severity. Further research is warranted to fully understand the anti-anxiety effects of a combination dietary counseling and omega-3 supplementation intervention.
						Quality of Life (PROMIS-29 v2.1)	NR	NR	
Bayes et al. (52)	RCT	MediDiet	12 weeks (Assessments at baseline, 6 wks, and 12 wks)	Befriending (Social Control)	Males (aged 18–25) diagnosed with major depressive disorder (MDD) by a general medical practitioner (*n* = 75)	Depression (BDI)	*p* < 0.001	NR	Our results demonstrate that compared with befriending, an MD intervention leads to significant increases in MEDAS, decreases in BDI-II score, and increases in QoL scores. These results highlight the important role of nutrition for the treatment of depression and should inform advice given by clinicians to this specific demographic population.
						Total (WHOQOL-BREF)	*p* < 0.001	NR	
						Psychological (WHOQOL-BREF)	*p* < 0.001	NR	
Bourdel-Marchasson et al. (53)	RCT	Evaluated Four Modified Versions of a Healthy Anti-Inflammatory Diet	8 wks (Assessments at baseline, 1 mo, and 2 mo)	Comparison (Randomization to 1 of 4 diet and supplement groups (see T1))	Healthy older adults (65–80 year) (*n* = 125)	QoL Mental Component (MOS SF36- MCS) (Arm A)	*p* = 0.020	NR	Depressive symptoms of healthy older subjects decreased after a two-month healthy diet adapted to three different European countries’ populations, independently of nutraceutical intake. The decrease appeared to be continuous with time, with an intermediate value at 1 month. There was no consistent change in any of the other clinical variables. This healthy diet and the nutraceutical products were given with the aim of decreasing systemic inflammation and oxidative stress and optimizing gut microbiota, as reported previously.
						QoL Mental Component (MOS SF36- MCS) (Arm B)	NS	NR	
						QoL Mental Component (MOS SF36- MCS) (Arm C)	*p* = 0.025	NR	
						QoL Mental Component (MOS SF36- MCS) (Arm D)	NS	NR	
						Depression (CES-D) (Arm A)	*p* = 0.001	NR	
						Depression (CES-D) (Arm B)	*p* = 0.023	NR	
						Depression (CES-D) (Arm C)	*p* = 0.004	NR	
						Depression (CES-D) (Arm D)	*p* = 0.021	NR	
						Anxiety (STAI) (Arm A)	NS	NR	
						Anxiety (STAI) (Arm B)	NS	NR	
						Anxiety (STAI) (Arm C)	NS	NR	
						Anxiety (STAI) (Arm D)	NS	NR	
Bozdoğan et al. (54)	RCT	MedDiet + Probiotic Supplementation	12 weeks [Assessments at baseline (24 wks gestation), 30 wks, and 36 wks]	Control Group	Pregnant women aged 20–40 years diagnosed with gestational diabetes mellitus (GDM)	Depression (EPDS)	*p* = 0.001	NR	In GDM patients, the Mediterranean diet quality had a significant negative relationship with depression and anxiety, and a positive relationship with maternal attachment. The group receiving probiotic supplementation had lower scores of depression and anxiety and lower gestational weight gain. However, the Mediterranean diet score was higher in the probiotic group at week 36. Further well designed randomized controlled trials with longer supplementation durations are needed for a stronger assessment of probiotic supplementation on maternal and fetal long-term mental health.
						Anxiety (PrAS)	*p* = 0.004		
Cabrera-Suárez et al. (55)	RCT	MediDiet enriched w/ extra virgin olive oil	24 mos (Assessments at baseline, 4 mos, 8 mos, 16 mos, 20 mos, and 24 mos)	Control group (routine care)	Adults w/1 depression episode w/in the last 5 years in total or partial clinical remission in the last 6 months (n = 208)	Depression (BDI)			Our results indicate that a Mediterranean diet–based nutritional intervention enriched with EVOO in addition to usual treatment, compared with usual care without nutritional intervention did not show efficacy regarding relapse prevention but might, to a greater extent, significantly reduce subsyndromal depressive symptoms in patients with remitted major depression.
						4-months (intention-to-treat)	*p* = 0.021	NR	
						4-months (completers)	*p* = 0.010	NR	
						8-months (intention-to-treat)	*p* = 0.102	NR	
						8-months (completers)	*p* = 0.030	NR	
						16-months (intention-to-treat)	*p* = 0.723	NR	
						16-months (completers)	*p* = 0.657	NR	
						20-months (intention-to-treat)	*p* = 0.017	NR	
						20-months (completers)	*p* = 0.024	NR	
						24-months (intention-to-treat)	*p* = 0.403	NR	
						24-months (completers)	*p* = 0.405	NR	
Carcelén-Fraile et al. (56)	RCT	12- week combined resistance and Mediterranean diet program	12 weeks (Assessments at baseline and at the end of the 12-week intervention)	Control group	Older adults aged 65 and older	Depression (HADS)	*p* = 0.000	d = 0.56	The study demonstrated that a 12-week resistance program is effective in improving various psychological and health parameters in older adults aged 65 or older. Significant improvements were observed in levels of anxiety, depression, and perceived stress, as well as in several aspects of sleep quality. in the group that participated in the resistance program compared to the control group. The significant improvements in these parameters suggest that a resistance program is a valuable intervention to promote mental health and well-being in this population, offering a viable non-pharmacological therapeutic option.
						Anxiety (HADS)	*p* = 0.003	d = 0.38	
						Stress (PSS)	*p* = 0.025	d = 0.15	
						Sleep Quality (PSQI)	*p* = 0.000	d = 0.68	
Casas et al. (33)	RCT	MedDiet w/ extra-virgin	2023 - ≈ 14–17 weeks (Assessments at baseline	Mindfulness-Based Stress Reduction Program	Pregnant women w/a high risk of developing a small for gestational	Stress (PSS) Within-group	*p* = 0.035	NR	In conclusion, a MedDiet intervention significantly reduces maternal anxiety and stress, as well as improving well-being and sleep quality during gestation. Considering the increasing importance of the role of mental health during pregnancy, these findings might imply the promotion of a pregnancy-adapted MedDiet among pregnant women as a powerful public health strategy.
						Stress (PSS) Frequency above 75th percentile	*p* = 0.96	NR	
						STAI (anxiety)			
						Within-group	p = 0.004	NR	
		Olive oil and walnuts	19–23 wks gestation and at 34–36 wks gestation)	Control group	age newborn (*n* = 1,221)	Frequency above 75th percentile	*p* = 0.082	NR	
Casini et al. (57)	RCT	MedDiet	8 wks (Assessments at baseline, 4 wks, and 8 wks)	Control Group	100 Outpatients aged 18–65 years old with fibromyalgia; 84 completed the study	Anxiety (SAS)	*p* < 0.05	NR	People with fibromyalgia have poor eating habits, which could be a cause of the disease. A personalized Mediterranean diet with fewer proinflammatory substances seems to reduce the disability and fatigue associated with FM. Although further studies are needed to support the present results in a long-term followup with a larger number of cases, our generally positive outcomes suggest that the dietary approach should be adopted for the treatment of fibromyalgia.
						Depression (SDS)	*p* < 0.05		
						Fatigue (MAF)	*p* < 0.05		
Daneshzad et al. (58)	RCT (Parallel)	DASH Diet	12 weeks (Assessments at baseline and at 12 wks)	Control group	Postmenopausal females w/ insulin-resistance condition (*n* = 60)	Depression (DASS21)	*p* = < 0.0001	d = 0.234	In conclusion, the DASH diet improved the biochemical markers in the intervention group and had beneficial effects on depression, anxiety, and stress scores, as well as sleep status.
						Anxiety (DASS21)	*p* = 0.026	d = 0.135	
						Stress (DASS21)	*p* = 0.005	d = 0.167	
Diekmann et al. (29)	Randomized Crossover Trial	Evaluated 4 Dietary and Exercise Conditions (Western diet high-fat or Mediterranean-type diet plus 30-min rest or walking)	6 weeks (Assessments at baseline, 1.5 h, 3.0 h, and 4.5 h post-meal)	Crossover Design	Adults w/ Metabolic syndrome traits (*n* = 26)	Subjective Mood (MDBF)			In conclusion, the present study shows no relevant effect of meal composition or postprandial activity behavior regarding subjective mood and none of the four treatment conditions can be rated superior in older adults with a risk phenotype for cardiovascular diseases. Compared to a Western diet high-fat meal, a meal composition reflecting the Mediterranean dietary pattern seems to be beneficial regarding postprandial attention. After the consumption of WD, postprandial resting seems to be more beneficial than postprandial walking for optimal cognitive performance. Due to its nutrient composition and food items (e.g., higher amount of low-energy/nutrient-dense foods and higher fiber content), MD leads to a stronger and longer lasting feeling of satiety. A selection of food items in accordance with the Mediterranean dietary pattern might therefore have a positive impact on weight regulation and should be of special importance in overweight-to-obese subjects.
Dolatkhah et al. (59)	RCT (Parallel)	Anti-inflammatory diet	8 wks (Assessments at baseline and end of the 2-month intervention)	Low-calorie diet	Overweight or obese women (≥ 40 years) with mild to moderate knee osteoarthritis (*n* = 60)	Depression (BDI)	p = 0.003	NR	Mental symptoms, depression, anxiety, QoL-physical function, and QoL-pain scores significantly improved in the anti-inflammatory compared to low-calorie diet after intervention
						Anxiety (BAI)	*p* = 0.011	NR	
						Mental Health (SF-36)	*p* = 0.191	NR	
						Emotional Well-being (SF-36)	*p* = 0.377	NR	
Felicetti et al. (60)	RCT	MedDiet	12 months (6 months dietetic intervention + 6 months observation) (assessments at baseline, 6 mos, and 12 mos)	Different patterns of Mediterranean hypocaloric diets	53 adults older than 18 with a diagnosis of MS in accordance with the 2017 McDonald’s criteria (Thompson et al., 2018) and body mass index (BMI) ≥ 25 kg/m2	Depression (The Multiple Sclerosis Performance Test)	NS	NR	Dietetic interventions in overweight pwMS, irrespective of their specific macronutrients composition, are able to improve the cardiovascular profile, the perception of subjective symptoms as well as objective disability score
						Anxiety (The Multiple Sclerosis Performance Test)	*p* = 0.012	NR	
						Quality of Life (Neuro-QoL)	NS	NR	
Francis et al. (61)	RCT	Australian Guide to Healthy Eating + Mediterranean-style diet recommendations	3 weeks (Assessments at baseline and 3 wks; with an additional follow-up at 3 mos)	Habitual Diet	Young adults w/ elevated levels of depression symptoms & a poor diet (*n* = 101)	Depression (CESD-R)	*p* = 0.007	d = 0.65	The Diet group had significantly lower self-reported depression symptoms than the Control Group. These results are the first to show that young adults with elevated depression symptoms can engage in and adhere to a diet intervention, and that this can reduce symptoms of depression. The findings provide justification for future research into the duration of these benefits, the impacts of varying diet composition, and their biological basis.
						Depression (DASS)	*p* = 0.002	d = 0.75	
						DASS-Anxiety (DASS)	*p* = 0.032	d = 0.54	
						Stress (DASS)	p = 0.004	d = 0.70	
						Anger (POMS Subscale)	NS (trend)	NR	
						Depression (POMS Subscale)	NS	NR	
						Confusion (POMS Subscale)	NS	NR	
						Tension (POMS Subscale)	NS	NR	
						Vigor (POMS Subscale)	NS	NR	
						Fatigue (POMS Subscale)	NS	NR	
Gazzellone et al. (20)	RCT	Mad dog diet (anti-inflammatory)	1 month (Assessments at baseline, immediately post-consultation, and 1-month follow-up)	Control group	1 year removed or more from either spinal cord injury (SCI) or neurological diagnosis. All participants but one (muscular dystrophy) had either SCI or multiple sclerosis (*n* = 11)	Depression (CES-D)			In addition, the consultation resulted in improved dietary adherence and mood measured at one-month postintervention. Last, improvements in dietary adherence and barrier self-efficacy were both significantly associated with improved mood.
						group x time	*p* = 0.038	NR	
						between groups at baseline	*p* = 0.604	NR	
						between groups post-intervention	*p* = 0.604	NR	
						control group post-intervention	NS	NR	
						intervention group post-intervention	*p* = 0.02	NR	
Golmohammadi et al. (63)	RCT	MIND low-calorie diet (MLCD)	12 weeks (Assessments at baseline and at end of intervention [12 wks])	Control Group	44 patients with type 2 diabetes and insomnia in Sanandaj, Iran.	Depression (DASS)	*p* = 0.001	NR	In conclusion, adherence to the MIND diet resulted in significant improvement in sleep quality and mental health in type 2 diabetic women with insomnia. Moreover, the MIND diet group exhibited a significant increase in serum BDNF levels and a significant decrease in cortisol levels and measurements of waist circumference, WHR and WHR after a 12-week intervention compared to the control group. These results suggest a promising therapeutic potential of the MIND diet in T2DM. However, future well-designed studies with larger sample sizes and objective measurement tools such as PSG and actigraphy are needed to fully clarify the effects of the MIND diet on these variables. Furthermore, the assessment of serum levels of melatonin, serotonin, tryptophan and kynurenine is recommended to better understand the underlying mechanisms of action.
						Anxiety (DASS)	*p* = 0.001		
						Stress (DASS)	*p* = 0.001		
Hardman et al. (64)	RCT	MedDiet and Exercise	6 months (Assessments at baseline and 6 mos)	Regular Lifestyle (no intervention)	Older adults (age between 60 and 90) living independently in retirement and independent-living facilities (*n* = 148)	Depression (DASS)	NS	0.03	In conclusion, the primary outcome, the CompM, did not show a significant change in any of the 3 intervention groups relative to the control group; however, there was a significant improvement in SWMin the combined diet and exercise group relative to the control group. In addition, this combined exercise and diet group also demonstrated a reduction in negative mood symptoms as assessed by the DASS score over the trial, as did the exercise-only group. This suggests that the interventions, or at least the exercise component, may also improve mood. There were no significant cardiovascular effects and few blood biomarker changes. Given the number of measures assessed and the small sample size, any interpretation of potential mechanisms from this study would be speculative. We therefore suggest that a study with more participants over a longer time frame is required to further investigate our finding that a combined MedDiet and walking intervention may improve SWM and mood in older people. With the proliferation of aged-care and retirement facilities withinAustralia, strategies to keep residents both physically and cognitively healthy are of paramount importance. It is critical to have more evidence-based research to substantiate efficacy, thereby supporting such intervention programs
						Anxiety (DASS)	NS	0.04	
						Stress (DASS)	NS	0.04	
						Negative Emotion (DASS)	*p* < 0.05	0.13	
						Mood (POMS)	NS	0.08	
						Perceived Wellness	*p* < 0.05	0.07	
Ibarra et al. (62)	RCT	MedDiet	6 months, with outcome measures assessed at baseline and again at 6 months.	No Med Diet	Adults meeting DSM-IV criteria for MDD, dysthymic disorder, or bipolar disorder, currently on antidepressant treatment, & recent plasma micronutrient level assessment (*n* = 77)	Depression (HADS)	*p* = 0.08	NR	Selenium levels in this sample at baseline were moderately low and slightly correlated to depression severity. However, these levels were still low 6 months after in both control and active group despite the fact that the active group received a prescription of the MedDietpattern. The rest of micronutrients analyzed (zinc, iron, magnesium, folic acid and vitamin B12) neither changed. Low serum levels of all these micronutrients can be related to depression, so the low serum levels of zinc found in the present study may suggest that there could be an etiopathogenic role of this element in the patients depression symptoms as well
						Depression (BDI)	*p* = 0.05	NR	
						Illness severity (CGI)	*p* = 0.03	NR	
Jacka et al. (65)	RCT	ModiMedDiet	12 weeks (Assessments at baseline and at 12-week endpoint)	Social Support	Adults meeting DSM-IV criteria for MDE and scored ≥ 18 on the Montgomery–Åsberg Depression Rating Scale (MADRS); ≤ 75d on a Dietary Screening Tool (DST) (*n* = 67)	Depression (MADRS)	*p* < 0.001	d = 1.16	These results indicate that dietary improvement may provide an efficacious and accessible treatment strategy for the management of this highly prevalent mental disorder, the benefits of which could extend to the management of common co-morbidities.
						Depression (HADS)	*p* = 0.032	d = 0.632	
						Anxiety (HADS)	*p* = 0.033	d = 0.594	
						Symptom change (CGI-I)	*p* = 0.156	NR	
						Mood (POMS)	*p* = 0.127	NR	
Jeitler et al. (66)	RCT	=x Fasting + Lifestyle Modification	24 weeks (Assessments at baseline, 1 wk., 12 wks, and 24 wks)	Lifestyle Modification	Patients w/a metabolic syndrome (NCEP-ATP-III criteria), diagnosed with systolic hypertension and/or additional subclinical atherosclerosis (<50% coronary artery stenosis, <50% carotid artery stenosis, peripheral artery disease stage 1) (*n* = 145).	Mental health (SF-36 subscale)			After the multimodal lifestyle modification intervention, we found improvements for all outcome measures, namely quality of life, anxiety, depression, stress, mood as well as self-efficacy, mindfulness, and self-compassion, which persisted at the follow-up after 24 weeks
						Week 1 between groups	*p* = 0.444	d = 0.12	
						Week 12 between groups	*p* = 0.128	d = 0.23	
						Week 24 between groups	*p* = 0.887	d = 0.03	
						SF-36 mental component			
						Week 1 between groups	*p* = 0.129	d = 0.22	
						Week 12 between groups	*p* = 0.096	d = 0.24	
						Week 24 between groups	*p* = 0.594	d = 0.10	
						Emotional (SF-36 subscale)			
						Week 1 between groups	*p* = 0.495	d = 0.13	
						Week 12 between groups	*p* = 0.504	d = 0.12	
						Week 24 between groups	*p* = 0.957	d = 0.01	
						Anxiety (HADS)			
						Week 1 between groups	*p* = 0.303	d = 0.16	
						Week 12 between groups	*p* = 0.227	d = 0.18	
						Week 24 between groups	*p* = 0.206	d = 0.21	
						Depression (HADS)			
						Week 1 between groups	*p* = 0.386	d = 0.15	
						Week 12 between groups	*p* = 0.432	d = 0.13	
						Week 24 between groups	*p* = 0.740	d = 0.07	
						Depression (POMS Subscale)			
						Week 1 between groups	*p* = 0.049	d = 0.30	
						Week 12 between groups	*p* = 0.397	d = 0.11	
						Week 24 between groups	*p* = 0.504	d = 0.12	
						Fatigue (POMS Subscale)			
						Week 1 between groups	*p* = 0.014	d = 0.50	
						Week 12 between groups	*p* = 0.206	d = 0.23	
						Week 24 between groups	*p* = 0.549	d = 0.12	
						Vigor (POMS Subscale)			
						Week 1 between groups	*p* = 0.629	d = 0.10	
						Week 12 between groups	*p* = 0.033	d = 0.45	
						Week 24 between groups	*p* = 0.147	d = 0.30	
						Anger (POMS Subscale)			
						Week 1 between groups	*p* = 0.089	d = 0.27	
						Week 12 between groups	*p* = 0.412	d = 0.14	
						Week 24 between groups	*p* = 0.192	d = 0.23	
						Stress (CPSS)			
						Week 1 between groups	NA	NA	
						Week 12 between groups	*p* = 0.070	d = 0.33	
						Week 24 between groups	*p* = 0.064	d = 0.37	
Kabiri et al. (67)	RCT	Mediterranean-DASH Intervention for Neurodegenerative Delay (MIND) diet	8 weeks (Assessments at baseline, week 4, week 6, and week 8)	Control Group	Fifty-two overweight or obese (BMI ≥ 25 kg/m2) women ages 18–45 in reproductive age diagnosed with PCOS based on diagnosed with PCOS based on Rotterdam criteria	Depression	*p* = 0.011	NR	Our results support our hypothesis stating that the MIND diet intervention can lead to significant improvements in mental health parameters and certain domains of the PCOSQ in individuals with PCOS. However, no significant changes in hirsutism or metabolic and hormonal parameters were observed. These results support the potential role of dietary interventions in managing psychological well-being and improving quality of life in individuals with PCOS. Further research is warranted to explore the long-term improving quality of life in individuals with PCOS. Further research is warranted to explore the long term effects of the MIND diet and to elucidate the underlying mechanisms of its impact on mental health in this population.
						Anxiety	*p* = 0.024		
						QoL - Emotion	*p* = 0.007		
						Qol - Body Hair	*p* = 0.040		
Lee et al. (68)	Randomized Crossover Design	MedDiet	21 days (Assessments at baseline, day 11, and day 21)	Normal Diet/No Change	Healthy adult females (*n* = 24)	Mood (POMS)	NS	NR	This study indicated that in short-term, switching to MedDiet can improve aspects of mood and cardiovascular health in young, healthy women.
						Anger (POMS Subscale)	NS	NR	
						Tension (POMS Subscale)	NS	NR	
						Confusion (POMS Subscale)	*p* = 0.02	NR	
						Vigor (POMS Subscale)	NS	NR	
						Depression (POMS Subscale)	NS	NR	
						Fatigue (POMS Subscale)	NS	NR	
						Alertness (VAS)	*p* < 0.01	NR	
						Calmness (VAS)	NS	NR	
						Contentment (VAS)	*p* = 0.02	NR	
Long Parma et al. (69)	RCT	Anti-inflammatory diet	12 months (Assessments at baseline, 6 mos, and 12 mos)	Control group	Adult women (BMI ≥ 25 kg/m2), early-stage (0-III) breast cancer survivors who had completed their treatment 2 or more months prior to enrollment (*n* = 153)	Stress (PSS)	*p* = 0.01	NR	It is not possible to determine the exact nature of the reduction in stress occurring in the intervention. It could be that it was related to the participants’ feeling of empowerment from the intervention in that it provided guidance and hands on workshops to increase their intake of anti-inflammatory foods; this could have lowered feelings of stress related either to their previous diagnosis of breast cancer, stress related to body weight (as participants met criteria for overweight/obesity), some combination of both, or some other source of stress
						Emotional Well-Being (FACT-G (EWS))	*p* = 0.76	NR	
						Depression (CES-D)	*p* = 0.51	NR	
McMillan et al. (70)	RCT	MediDiet (nutrient-dense)	10 days (Assessments at baseline and day 10)	No Change Group	Healthy adult females (*n* = 27)	Depression (POMS Subscale)	*p* = 0.58	NR	Compared to the no change group, the Diet Change group showed significant improvements in self-rated vigor, alertness, and contentment. These preliminary findings require verification in larger trials but suggest that appropriate dietary change may benefit mood and some aspects of cognitive performance in healthy adults.
						Anxiety (POMS Subscale)	*p* = 0.51	NR	
						Anger (POMS Subscale)	*p* = 0.32	NR	
						Vigor (POMS Subscale)	*p* = 0.00	NR	
						Fatigue (POMS Subscale)	*p* = 0.16	NR	
						Confusion (POMS Subscale)	*p* = 0.67	NR	
						Mood (POMS)	*p* = 0.06	NR	
						Bond-Lader VAS	*p* = 0.00	NR	
						Alert (Subscale)	*p* = 0.00	NR	
						Content	*p* = 0.00	NR	
						Calm	*p* = 0.38	NR	
Miralles-Amorós et al. (71)	RCT	ediDiet	12 weeks (Assessments at baseline, mid-intervention, and post-intervention)	Free Diet, High Antioxidant Diet	21 young adult female professional handball players (*n* = 21).	Depression (POMS Subscale)			In conclusion, following different personalized dietary-nutritional plans based on current recommendations for athletes leads to improvements in mood and body image in young female professional handball players with low energy availability
						effect time	*p* = 0.805	NR	
						effect time x group	*p* = 0.303	NR	
O’Connor et al. (72)	RCT	Medi-pattern diet	16 weeks total (Two 5-wk Mediterranean diet interventions separated by a ≥ 4-wk washout; assessments at baseline and end of each 5-wk intervention)	No Change in Diet	Adults w/a BMI between 25 and 37	Tension (POMS Subscale)	*p* = 0.11	NR	Following a Med-Pattern, independent of lean, unprocessed red meat intake, may not be an effective short-term strategy to meaningfully improve indexes of personal well-being in adults who are overweight or obese.
						Depression (POMS Subscale)	*p* = 0.30	NR	
						Anger (POMS Subscale)	*p* = 0.08	NR	
						Vigor (POMS Subscale)	*p* = 0.38	NR	
						Fatigue (POMS Subscale)	*p* = 0.039	NR	
						Confusion (POMS Subscale)	*p* = 0.14	NR	
						Mood (POMS)	*p* = 0.07	NR	
						Vitality (QoL)	*p* = 0.02	NR	
Papandreou et al. (73)	RCT	MediDiet (Clinical Decision Support System (CDSS))	3 mos (Assessments at baseline and at 3 mos)	Control group (general lifestyle guidelines)	Healthy pregnant women (*n* = 40)	Anxiety (HADS)	*p* = 0.048		In conclusion, our results support the use of CDSS during pregnancy for improving MD adherence and overall psychological distress, as evidenced by better anxiety and depression scores
						Depression (HADS)	*p* = 0.006		
Parletta et al. (74)	RCT	Mediterranean-style diet	6 months (Assessments at baseline, 3 mos, and 6 mos)	Social Comparison Group	Adults (age 18 and 65 years) and diagnosed or self-reported depressive symptoms over the previous 2 months or longer (*n* = 85)	Depression (DASS)	*p* = 0.027	NR	This study supports preliminary findings from other dietary interventions that showed evidence of reduced depression in people with high CVD risk,17 metabolic syndrome,57 and unexpectedly in a trial of problem solving in elderly adults at risk for depression in the dietary arm that was used as a control.58 Our study is one of the first RCTs to show benefits of healthier diet for mental health in a cohort of people with depression. Another Australian study was recently published, reporting improvements in depressive symptoms of people suffering major depressive disorder, and 32.3% achieving remission over 12 weeks.59 In our study, the average depression score of the MedDiet group fell below the extremely severe depression cut-off, whereas in the social group it remained in the extremely severe range. Interestingly, while our study design was group based, the latter intervention involved one-on-one dietetic counseling in the dietary arm and ‘buddying’ as a control arm, and had similar findings. More studies with larger sample sizes and objective markers are required.
						Anxiety (DASS)	NS	NR	
						Stress (DASS)	NS	NR	
						QoL	*p* = 0.037	NR	
Radkhah et al. (75)	RCT	Mediterranean diet	12 weeks (Assessments at baseline and post-intervention at 12 wks)	Diet (Healthy Eating Pattern)	Individuals referred to a psychiatric clinic w/ depression, stress, or anxiety diagnosed for over a month (*n* = 60)	Depression (DASS)			Our initial findings showed that following the MedDietdid not have an impact on mental disorders and body measurements. This could be attributed to the participants’ low adherence to the instructions, which was mainly due to their financial situation. However, our results changed after adjusting for other factors that could have influenced the outcome. We observed a decrease in scores related to mental disorders and body measurements. Despite some significant findings, the results were not noticeable from a clinical perspective. Based on our findings and the study’s limitations, there are indications that individuals who follow the MedDietmay benefit from the advice to improve their weight and mental well-being.
						Independent samples t-test	*p* = 0.714	NR	
						Paired samples t-test	*p* = 0.701	NR	
						Model 1	*p* = 0.429	NR	
						Model 2	*p* = <0.001	NR	
						Model 3	*p* = 0.302	NR	
						Anxiety (DASS)			
						Independent samples t-test	*p* = 0.142	NR	
						Paired samples t-test	*p* = 0.946	NR	
						Model 1	*p* = 0.142	NR	
						Model 2	*p* = <0.001	NR	
						Model 3	*p* = 0.054	NR	
						Stress (DASS)		NR	
						Independent samples t-test	*p* = 0.648	NR	
						Paired samples t-test	*p* = 0.394		
						Model 1	*p* = 0.670	NR	
						Model 2	*p* = <0.001	NR	
						Model 3	*p* = 0.054	NR	
						Negative emotion (DASS)			
						Independent samples t-test	*p* = 0.611	NR	
						Paired samples t-test	*p* = 0.591	NR	
						Model 1	*p* = 0.694	NR	
						Model 2	*p* = <0.001	NR	
						Model 3	*p* = 0.915	NR	
Rumbo-Rodríguez et al. (76)	RCT	MedDiet based nutritional treatment (plus physical activity program, Nutritional education sessions + individualized dietary)	2 yrs. (Assessments at baseline, 6 mos, 12 mos, and 24 mos)	Mediterranean nutritional education	60 years w/a body mass index (BMI) greater than 25 kg/m2 (*n* = 45)	Depression (PHQ-9)	*p* = 0.557	NR	In the present study, it was observed that throughout the two-year intervention, both the experimental and control groups improved their scores on the PHQ-9 questionnaire; however, no improvement in the scores for the GAD-7 questionnaire was observed.
						Anxiety (GAD-7)	*p* = 0.236	NR	
Sabet et al. (26)	RCT (pilot)	Nordic diet	8 days (Assessments at baseline and post-intervention on day 8)	Control diet	Adults mild or moderate depression (w/a score between 13 and 34 on the MADRS-S) (*n* = 16)	Depression (MADRS-S subscale)	*p* = 0.845	NR	Furthermore, the majority of health-related self-reported outcomes improved in both groups, with a greater improvement in depression in the ND group compared to the CD group. This study lays the foundation for a future 8-week RCT in which the aim will be to investigate whether a healthy Nordic diet improves depressive symptoms, as well as the biological mechanisms underlying this effect and potential factors that may predict treatment response
						Total Score (MADRS-S)	*p* = 0.792	NR	
Sánchez-Villegas et al. (77)	RCT	MediDiet+EVOO or MediDiet+nuts	3–6 yrs. (Assessments at baseline, every 3 mos for dietary counseling, and annually for follow-up measures)	Low-Fat Diet	Adults (55–80 yrs) at high cardiovascular disease risk (no previously documented CVD) (*n* = 7,447)	Depression	NS	NR	Results from this analysis are suggestive of a beneficial effect of a long-term intervention with a MedDieton depression for patients with DM2. To definitely assess the role of MedDiet in the prevention of depression, longer follow-up of this trial and further experimental investigations are needed.
Staudacher et al. (31)	RCT	MediDiet counseling	6 weeks (Assessments at baseline and 6 wks)	Habitual diet	Adults w/ IBS & mild or moderate anxiety and/or depressive symptoms (*n* = 59)	Anxiety (HADS)	*p* < 0.001	NR	A MedDiet is feasible in IBS and leads to improvement in gastrointestinal and psychological symptoms
						Depression (HADS)	*p* = 0.011	NR	
						Stress (PSS)	*p* < 0.001	NR	
Toobert et al. (78)	RCT	MeditDiet Lifestyle Program	24 months (Assessments at baseline, 6 mos, 12 mos, and 24 mos)	Usual Care	Postmenopausal women w/ type 2 diabetes (*n* = 279)	Depression (CES-D)	NS	NR	Long-term maintenance of changes made in lifestyle interventions, especially multiple-risk- factor interventions, are largely absent from study results [18], and maintenance is a major challenge [19,14]. In this study, participants randomized to the MLP, compared to a usual care (UC) control condition, showed greater long-term (12- and 24-month) improvements in multiple lifestyle behaviors, including eating patterns, physical activity, and stress management as well as psychosocial outcomes. Additional research is needed to determine whether these improvements could be translated into further reductions of health-related costs and with diverse populations.
						Stress (PSS)	NS	NR	
Torres et al. (79)	Randomized Crossover Design	LNAHK Diet (modified DASH diet, low in sodium and calcium)	13 weeks (Assessments weekly; saliva samples at end of each 2-week control and 4-week test diet phase)	DASH Like Diet	Adults (>25 years old) and had a BP in the high/normal range (≥ 120 mmHg systolic BP or ≥ 80 mmHg diastolic BP) (*n* = 97)	POMS Global Score	*p* < 0.05	NR	The LNAHK intervention diet seems to overall improve mood compared to a high-calcium (HC) diet with a moderate-sodium, high-potassium DASH-type diet.
						Anger (POMS Subscale)	NS	NR	
						Confusion (POMS Subscale)	NS	NR	
						Depression (POMS Subscale)	*p* < 0.05	NR	
						Fatigue (POMS Subscale)	NS	NR	
						Tension (POMS Subscale)	*p* < 0.05	NR	
						Vigor (POMS Subscale)	*p* < 0.05	NR	
Torres and Nowson (12),	Randomized Crossover Design	Moderate sodium DASH-type diet	14 wks (Assessments at baseline ×2; mood every 2 wks at weeks 2, 4, 6, 8, 10, 12, 14; 24-h urine at baseline ×2 and weeks 4, 8, 12, 14)	DASH Diet	Adults (>25 years old) and had a BP in the high/normal range (≥ 120 mmHg systolic BP or ≥ 80 mmHg diastolic BP) (*n* = 97)	Mood (POMS)	*p* < 0.01	NR	Moderate sodium DASH-type diets, rich in fruits and vegetables and included red meat on most days, appeared to improve mood to a greater extent than the “HD” (Healthy Diet), particularly in relation to anger. Both diets showed an overall improvement in mood over the length of the intervention. An increase in lean red meat and fruit was related to an improvement in depression and confusion, respectively.
						Anger (POMS Subscale)	*p* < 0.02	NR	
						Tension (POMS Subscale)	*p* < 0.03	NR	
						Confusion (POMS Subscale)	*p* < 0.04	NR	
						Vigor (POMS Subscale)	*p* < 0.05	NR	
						Depression (POMS Subscale)	*p* < 0.06	NR	
						Fatigue (POMS Subscale)	*p* < 0.07	NR	
						Mood Global Score (POMS)	*p* < 0.08	NR	
Wade et al. (80)	RCT	MedDiet w/ lean pork	24 weeks total (8 wks on MedPork diet + 8 wks washout + 8 wks on low-fat control; assessments at baseline, 8 wks, 16 wks, and 24 wks)	Low Fat Diet	Adults (45–80 years old) w/an increased risk of developing cardiovascular disease (CVD)	Mood (POMS)	*p* = 0.12	NR	Our findings indicate that a MedDietinclusive of fresh, lean pork can be adhered to by an older non-Mediterranean population while leading to positive cognitive outcomes. The current study aimed to examine the cognitive effects of a MedDietwith 2–3 weekly servings of fresh lean pork. Our findings show that the MedPork intervention was capable of improving processing speed and mood in a population at risk of CVD and dementia. Our findings provided further support for the cognitive effects of the Mediterranean diet, and the pathway through which the diet may influence cognitive function.
						Tension (POMS subscale)	*p* = 0.20	NR	
						Depression (POMS subscale)	*p* = 0.08	NR	
						Anger (POMS subscale)	*p* = 0.20	NR	
						Confusion (POMS subscale)	*p* = 0.36	NR	
						Fatigue (POMS subscale)	*p* = 0.31	NR	
						Vigor (POMS subscale)	*p* = 0.11	NR	
						Mental Wellbeing (SF-36)	*p* = 0.19	NR	
						Role emotional (SF-36)	*p* = 0.03	NR	
						Mental health (SF-36)	*p* = 0.10	NR	
						Well-being (SF-36)	*p* = 0.03	NR	
Wade et al. (81)	Randomized Control Parallel Crossover	MedDiet supplemented with diary foods	24 weeks (total; 8 wks MedDairy + 8 wks Low-Fat with 8-wk washout) (Assessments at baseline, 8 wks, 16 wks, and 24 wks)”	Low Fat Diet	Adults (45–80 years old) w/an increased risk of developing cardiovascular disease (CVD)	Mood (POMS)	*p* = 0.01	d = 1.8	A MedDiet supplemented with dairy foods (to meet Australian calcium recommendations) improved mood and processing speed in a sample of older adults at risk for dementia. Adding dairy to a MedDiet may be an effective intervention for the improvement of psychological well-being and cognitive function, which could have longer-term cognitive effects in an at-risk population.
						Tension (POMS Subscale)	*p* = 0.03	d = 1.8	
						Depression (POMS Subscale)	*p* = 0.03	d = 1.8	
						Anger (POMS Subscale)	*p* = 0.02	d = 1.8	
						Fatigue (POMS Subscale)	*p* = 0.070	NR	
						Vigor (POMS Subscale)	*p* = 0.818	NR	
						Confusion (POMS Subscale)	*p* = 0.004	d = 2.3	
						Role emotional (SF-36)	*p* = 0.15	NR	
						Mental Health (SF-36)	*p* = 0.43	NR	
Wardle et al. (82)	RCT	MedDiet	12 weeks (Assessments at baseline, 6 wks, and 12 wks)	Waiting List Control Group or Low Fat Diet	Adults w/ mildly or moderately raised serum cholesterol levels (*n* = 155)	Depression (BDI)	NS	NR	Two dietary interventions successfully lowered serum cholesterol levels and had no adverse effect on mood. Some evidence for relative impairment in cognitive function in treated groups for one cognitive test, but additional studies needed to determine relevance of this finding. No significant between-group differences for measures of cognitive functioning.
						Depression (POMS Subscale)	NS	NR	
						Anger (STAXI)	NS	NR	
						Anger (POMS Subscale)	NS	NR	
						Psychological Well-Being (GHQ-28)	NS	NR	
						Stress (PSS)	NS	NR	
Wright et al. (83)	RCT	MedDiet and Mindfulness	3 mos (Assessments at baseline and 3 mos; intervention delivered over 8 weekly sessions)	Attention Control Group and True Control Group	African Americans w/ mild cognitive impairment & hypertension (*n* = 12)	Stress (PSS)	NS	NR	Results show that the MIM-DASH diet intervention was feasible and culturally acceptable in African Americans with hypertension and MCI. The pilot study contributes important knowledge because few studies have focused on mindfulness plus diet education in older African American adults.
Yau et al. (84)	RCT	Mind Diet w/nutritional counseling + Forest Bathing	4 weeks (Assessments at baseline and post-intervention (week 4))	Control group (no intervention)	Older adults w/ hypertension (*n* = 72)	Mood Total (POMS)	*p* = 0.164	d = 0.09	Adopting a four-week MIND diet with four nutrition sessions on four consecutive weekdays or in addition to the MIND diet, practicing 2-h FB sessions on four consecutive weekends has been observed to have beneficial effects on cardiac and mental health in hypertensive Chinese older adults.
						Tension (POMS Subscale)	*p* = 0.265	d = 0.03	
						Depression (POMS Subscale)	*p* = 0.194	d = 0.06	
						Anger (POMS Subscale)	*p* = 0.499	d = 0.07	
						Fatigue (POMS Subscale)	*p* = 0.561	d = 0.04	
						Vigor (POMS Subscale)	*p* = 0.665	d = 0.01	
						Confusion (POMS Subscale)	*p* = 0.150	d = 0.08	
						State Anxiety (STAI-S)	*p* = 0.299	d = 0.08	
						Trait Anxiety (STAI-T)	*p* = 0.605	d = 0.02	

**Table 4:** Summary of Mental Health Outcomes and Conclusions Across Included Evidence Syntheses.

Citation	Evidence synthesis type	Included study designs*	Total # of studies	Intervention/ Dietary index & # of studies**	Inclusion criteria	Mental health outcome(s)	Effect size***	Population group(s)	General conclusion	Recommendations for future research
Altun et al. (104)	SR	RCT Longitudinal Cross-Sectional	26	MedDiet (no animal protein or byproduct alterations) (26)	- Human studies with participants aged over 18 years. - Observational or interventional designs with a control group. - Focus on the association between MedDiet and depression. - Measurement of dietary components using 24-h dietary recall, food record, food frequency questionnaire (FFQ), or similar instruments. - Assessment of depression through validated screening tools, clinical diagnosis by trained interviewers, or antidepressant prescription as a proxy for clinical diagnosis. - MedDiet intervention must include: High consumption of plant-based foods (e.g., vegetables, fruits) or inclusion of at least one of nuts, legumes, cereals, fish, or olive oil.	Depression	N/A	Adults (≥18 y)	The MedDiet provides a promising adjunct to existing depression treatments, with minimal side effects, reduced disease severity, and cost-effectiveness for global implementation.	- Conduct head-to-head clinical trials comparing mainstream treatments with the Mediterranean Diet (MedDiet) for depression therapy. - Address limitations by implementing high-quality observational studies and randomized controlled trials to establish a causal link between the MedDiet and depression. - Precisely define the MedDiet using objective measures, and include longitudinal studies and clinical trials in future research.
Bizzozero-Peroni et al. (91)	SR/MA	RCT	5	MedDiet (5)	- RCTs - Population of participants with depressive disorders/depressive symptoms - Focus on the association between MedDiet and depression.	Depression	N/A	Adults aged 22.0–53.3 years of age with depression	MD interventions appear to have substantial potential for alleviating depressive symptoms in people experiencing major or mild depression.	- More experimental studies with larger sample sizes are needed to allow moderator analyses to better understand differences in effects between types of dietary interventions and types of wellbeing outcomes.
DeSmet et al. (92)	SR/MA	RCT	19	Dietary interventions were included if they directly targeted daily dietary choices, meaning that the intervention should be available as a practice individuals could integrate in their daily life by directly changing consumption. The consumption behavior may include a certain food (e.g., apple), food groups (e. g., FV consumption) or a combination of food groups in a diet (e.g., Mediterranean diet, Paleo, Keto diet) (19 studies included in the systematic review, of which 13 in the meta-analysis)	- Any within-subject or between-subject experimental study with a control condition to which individuals were randomly assigned. - Studies were included if they targeted a healthy population, aged between 18 and 29 years or with an average sample age in this range. - Included studies needed to focus on mental well-being as an outcome.	Mental Well-being: This includes at least one dimension of psychological well-being (autonomy, environmental mastery, personal growth, positive relations with others, purpose in life, and/or self-acceptance), subjective well-being (positive affect, low negative affect, satisfaction with life), personal well-being (vitality, resilience, meaning or purpose, autonomy or control, engagement and competence), or social well-being (sense of belonging, feeling appreciated, perception of social support, social contact, feeling respected).	95% confidence interval CI 0.098 to 0.442; Z = 3.083, *p* = 0.002 size n = 682	General population of emerging adults (18–29 years)	Dietary interventions (g = 0.270, k = 14 studies) and a healthy diet (g = 0.312, k = 10 studies, excluding chocolate intake) were effective in improving mental well-being in emerging adults, showing a significant small positive effect on mental well-being.	- Explore possible moderators including intervention dose, frequency, and duration to assess a dose-response relation, as well as characteristics of intervention delivery (e.g., lab or home setting, individual or group) and acceptability and liking of the dietary change.- Include a wider variety of subdomains of mental well-being, as effects appear to differ between indicators. Social well-being in particular appears to be understudied.- Scales such as the Mental Health Continuum Short-Form, which measures emotional, psychological, and social well-being, should be used to better understand which dietary intervention impacts which aspect of mental well-being.- RE-AIM (Reach, Efficacy, Adoption, Implementation and Maintenance) studies and long-term cost-effectiveness studies should be conducted to contribute evidence on the relevance of promoting a healthy diet for mental well-being and mental illness prevention.- Healthy dietary advice, particularly promotion of a Mediterranean diet, cereal and protein breakfast, and fruit and vegetable consumption, should be included as a standard component in mental health promotion programs for young adults.
Dinu et al. (101)	UMR	RCT Observational (cohort prospective/ case–control/ cross-sectional)	25	MedDiet (25)	- Study design: systematic reviews including meta-analyses (quantitative analysis) of observational studies (prospective cohort studies, cross-sectional studies and case–control studies) or meta-analyses of randomized clinical trials (RCTs). - Study population: 418 years. - Outcomes: health outcomes (for example, overall mortality, cardiovascular diseases, cancer, cognitive disorders, metabolic disorders), modifications of metabolic risk parameters (for example, anthropometric measurements, blood pressure, flow mediated dilation, lipid profile and glycaemic profile) or modifications of inflammatory parameters (for example, proinflammatory cytokines). - The decision to include studies was made on the basis of the study title, study abstract and full-text screening.	Depression (3 articles)	N/A	Adults (≥18 y)	Three studies reviewed demonstrated a positive relationship of protection against depression with a greater adherence to the MedDiet.	- Ensure future interventions have sufficient power to detect clinical outcomes, considering the heterogeneity of dietary assessment methods and study design inadequacies. - Adopt uniform methodologies, analyze data rigorously, and provide detailed reporting of population, intervention, comparison, and outcome data to accurately quantify the association of MedDiet adherence with health outcomes and quality of life.
Eliby et al. (105)	SR	RCT (3) Cross-sectional (22) Cross-sectional/ Longitudinal data (7) Prospective cohort (12)	44	MedDiet score (14); Dietary Inflammatory Index (3) MedDiet Score (MDS) or its variants (14); Dietary Inflammatory Index (DII) (3); Dietary Approaches to Stop Hypertension (DASH) index (2); Mediterranean-DASH diet Intervention for Neurodegenerative Delay (1); Inflammatory Dietary Pattern (IDP) (1)	- Investigated the whole diet according to predefined diet quality indices/scores or empirically-derived dietary patterns from measurements of complete dietary intake (unless an RCT). - Included individuals with clinically diagnosed depression and/or anxiety as determined directly by diagnostic interview, physician-reported diagnosis or referral for treatment, diagnosis through a national database, or indirectly via self-reported psychotropic medication use. - Assessed community-dwelling adults or in−/outpatients 18 years or older.	Depression; Anxiety	N/A	Adults (≥18 y) clinically diagnosed with depression and/or anxiety.	The strong association between MedDiet indices and reduced depression suggests the MedDiet could serve as a cost-effective, safe, and easily modifiable adjunct alongside existing therapies for depression, although findings for anxiety and other diet quality measures varied or lacked consistency.	- Include clinical diagnoses for anxiety and depression in future research to enhance assessment validity. - Investigate dietary interventions alone and in combination with existing treatments to understand diet’s role in mental health disorders. - Explore the biological mechanisms underlying these relationships to design effective interventions.
Gianfredi et al. (102)	UMR	Meta-analyses (19)	19	MedDiet (6); Dietary Approaches to Stop Hypertension (2); Dietary Inflammatory Index (5)	- Population: Adults (>18 y) without comorbidities - Intervention: All diets or dietary patterns/dietary interventions - Comparison: No intervention or any diet or dietary patterns/dietary interventions - Outcome: Depressive symptoms, depression, depressed mood, consumption of anti-depressant medications, depressive feeling - Study design: Meta-analyses of original studies (both randomized controlled trials and observational studies)	Depression	N/A	Adults (>18 y) without comorbidities	The MedDiet and anti-inflammatory diets may be protective against the development of depression.	- Substantiate findings with high-quality randomized controlled trials (RCTs) - Adopt coherent and uniform methodology and analyses in further studies. - Ensure detailed reporting of participant characteristics, sample sizes, and data collection methods. - Enhance quantification of the association between different dietary patterns and depression.
Jafari and Behrouz (106)	SR	Observational (cohort, cross-sectional), Intervention	8	Nordic Diet (8)	- Assessment of the ND as the main exposure. - Studies focusing on neurological function and disorders, cognitive spectrum (including cognition, memory, attention span, learning), and psychosocial and emotional aspects (including depression and anxiety) were included. - Enrolled humans at all ages. - Analytical epidemiological studies, i.e., observational studies (prospective cohort studies, case–control, or cross-sectional) and intervention studies (randomized control trials, and non-randomized trials, i.e., pre-post studies). - Studies written in English and published in reputable journals.	Depression, Anxiety, Stress	N/A	Humans of all ages	One RCT, conducted over 8 days in Sweden, involved 16 women and men aged between 18 and 65 years with major depressive disorder in a controlled setting with provided foods. The trial demonstrated that adopting a healthy Nordic diet (ND) led to a greater reduction in depressive symptoms compared to a control diet.	- Employ stronger methodologies in future studies. - Conduct studies with longer durations. - Include larger sample sizes in research.
Lassale et al. (93)	SR/MA	Longitudinal (20), Cross-Sectional (21)	41	MedDiet (10); Dietary Inflammatory Index (DII) (9); DASH Diet (4)	- Exposure: Comprehensive dietary assessment methods (e.g., food frequency questionnaire, 24-h diet recall, food record, diet history) and utilization of an *a priori* dietary score or index. - Outcome: Clinical depression assessed through study staff evaluation, medical records, or self-reported clinician-diagnosed cases. Depressive symptoms measured via validated scales/questionnaires (e.g., CES-D) and defined using validated cutoffs (e.g., CES-D cutoff of 16 for 20-item scale). Use of antidepressant medication considered only in conjunction with clinical depression or depressive symptom assessment. - Design: Observational study (cross-sectional, cohort, case–control). - Population: General free-living populations, including outpatients and non-institutionalized individuals, without age restrictions.	Depression	MedDiet- 0.69 (0.59, 0.82), 95% CI; Anti-Inflammatory Diet- 0.71 (0.60, 0.84), 95% CI; DASH Diet- 0.90 (0.73, 1.12), 95% CI	Generally healthy Adults (≥18 y); Exclusions: Clinical depression diagnosis, adolescents, low-middle income countries	A strong association exists between high adherence to a MedDiet, reduced adherence to a pro-inflammatory diet, and decreased risk of depression and depressive symptoms. Research on the relationship between adherence to a DASH diet and depression, especially in adults, is limited and inconclusive.	- Conduct more prospective studies. - Design well-powered clinical trials. - Assess the role of dietary patterns in preventing the onset, severity, and recurrence of depressive episodes.
Madani et al. (107)	SR	Cohort, Cross-Sectional, Case–Control	36	MedDiet (36)	- Participants: Adults (excluding children and adolescents) - Exposure: MedDiet - Control Group: Any control group - Outcomes: Axis I disorders (depression, bipolar disorder, anxiety, schizophrenia, psychosis, obsessive-compulsive symptom, attention-deficit/hyperactivity disorder, post-traumatic stress disorder, and eating disorders including anorexia nervosa, bulimia nervosa, and binge eating disorder) - Study Design: Cohort, cross-sectional, and case–control studies - Language: English - Availability: Full-text publications	Depression, Anxiety	N/A	- 29 studies (80.56%) conducted in Europe. - The majority of studies (80.56%) included both sexes. - Mean participant age ranged from 21.42 to 92.7 years. - Cohort studies: Sample size range 273–167,698. - Cross-sectional studies: Sample size range 79–4,470. - Case–control studies: Sample size range 141–273.	MedDiet shows promise in managing Axis I disorders, particularly depression and anxiety. Patients with Axis I disorders, even those unsuitable for pharmacotherapy or psychotherapy, can consider this diet as an alternative or complementary treatment.	- Conduct future review studies with a broader literature search spanning beyond 5 years, covering various diets and age groups, including children, to explore associations with Axis I disorders. - Consider systematic reviews or meta-analyses of interventional studies to enhance control and prevention of Axis I disorders. - Address limitations by including substance use as an Axis I disorder in future systematic reviews for comprehensive coverage. - Expand databases included in literature searches beyond two for more comprehensive data retrieval.
Matison et al. (94)	SR/MA	Prospective observational	21	MedDiet (3) Dietary Inflammatory Index DII (2) Alternate Dietary Inflammatory Index ADII (1)	- Original studies investigating longitudinal association between diet and depression incidence at follow-up. - Population-based, community-dwelling adults ≥ 45 years at baseline (or study provided sub analysis of participants ≥ 45 years). - Any form of nutrition intake (e.g., food patterns, food groups, foods, micronutrients, or dietary supplements). - Prospective observational study.	Depression	MedDiet (3 articles), OR = 0.93 [0.84, 1.04]. DII/ADII, OR = 1.33 [1.04, 1.70]	Population-based, community-dwelling adults ≥ 45-years at baseline (or study provided sub analysis of participants ≥ 45-years)	- Higher inflammatory diets and Western dietary patterns are linked to increased risk of depression. - Mediterranean, “healthy” dietary patterns, or fish intake not associated with depression onset. - Strongest association found between Dietary Inflammatory Index (DII) and depression onset.	- Investigate more high-quality interventions and cohort studies with diverse adult populations. - Use clinical diagnosis of depression to enhance the robustness of study findings. - Enable better analysis by age group through clinical diagnosis of depression.
Molendijk et al. (95)	SR/MA	Prospective Cohort Studies	29	MedDiet (n = 5)	- Included studies examined the link between dietary patterns/food group consumption and depression incidence or symptom changes. - High-quality dietary patterns/groups aligned with expert-defined healthy criteria or authors’ definitions. - Unhealthy dietary patterns/groups were similarly defined.	Depression	Association between Adherence to High-Quality Diets and Depression Incidence: MedDiet: OR = 0.75 Tuscan Diet: OR = 0.64Association between Dietary Inflammatory Index and Depression Incidence:	N/A (not specified)	High-quality, healthy diets like the MedDiet are linked to lower incidence of depressive symptoms in a dose–response manner. Abundant data supports diet’s crucial role in onset of depressive illness.	- Mechanistic understanding of dietary exposure’s connection to depression. - Prospective cohort studies controlling for relevant confounders like metabolic illness/obesity incidence or baseline stress exposure. - Randomized controlled prevention trials to investigate dietary interventions’ efficacy.
O’Neill et al. (86)	SR	RCT	7	MedDiet (2)	- Participants: Adults (18 years or older) with medically diagnosed major depressive disorder, self-diagnosed depression, or measured depressive symptoms. - Intervention: Whole food/diet interventions included; manipulation of unfortified food items in Western food chain. - Study Design: Only randomized controlled trials considered - Outcomes: Depression levels measured using validated tools like Beck Depression Inventory.	Depression	N/A	- Most studies focused on adult populations (≥18 years), except one involving participants aged 17 to 35 years. - Two studies targeted adults across the lifespan, two focused on young adults (18–30 years), and one examined older participants (50–79 years)	The inconsistent nature of the studies limited the synthesis of the data; however, the studies collectively showed a reduction in scores assessing depression.	- Consider participants’ current dietary intake as a baseline or implement a ‘washout’ period before intervention. - Deliver interventions by qualified professionals to maximize outcomes. - Ensure intervention duration of at least three months to align with recommendations for other depression treatments. - Include clear measures of dietary compliance in study protocols. - Utilize biochemical markers to confirm dietary compliance where possible. - Account for bidirectional influences between nutrition and mental health. - Carefully select a relevant depression scale, such as HRSD, BDI-II, or CES-D, for future research to enable comparability across studies and ensure research quality.
Psaltopoulou et al. (96)	SR/MA	Case–control, longitudinal cohort, cross-sectional	22	MedDiet (22)	N/A (supplementary figure not available)	Depression (9 articles)	Depression Risk- 0.68 (0.54, 0.86), 95% CI 3	N/A (supplementary figure not available)	Moderate to high adherence to a MedDiet appears to lower the risk of depression and offers a protective effect against it.	- Encompass a longitudinal cohort design with sufficient (> 5 yr) follow-up periods. - Aim to understand underlying mechanisms of action linking the interaction of the MedDiet and biomarkers.
Rudzińska et al. (87)	SR	Cross-Sectional	9	MedDiet (9)	- Included studies focused on compliance with a MedDiet among respondents over 65 years old. - Severity of depressive symptoms assessed using screening questionnaires for mood in the elderly. - Compliance with the MedDiet assessed using the Food Frequency Questionnaire (FFQ) and the MedDiet Index (MDI).	Depression	N/A	- Eight studies focused on patients aged 65 and over, with one study focusing on patients aged 90 to 99 years. - Six studies included patients living in their own households, while two studies involved patients from hospital/clinic settings. - One study included patients from both households and nursing homes.	The findings suggest that adhering to the MedDiet principles among elderly individuals may have positive effects in preventing depressive symptoms. Further exploration is warranted to understand the relationship between this diet and its specific components with the mental well-being of elderly patients.	- Consider cognitive decline and memory issues when collecting nutritional history in elderly populations. - Be aware of memory disorders often coexisting with depression in older patients. - Plan nutritional interventions with consideration for age-related decline in adaptability, such as reduced acceptance of new tastes or products. - When introducing the MedDiet, incorporate familiar products while gradually adjusting to new ones based on patient needs. - Emphasize familiar MedDiet components commonly used in local cuisine, such as whole grain cereals, olive oil, legumes, tomatoes, garlic, and onions. - Aim for maximum compliance with the MedDiet while accommodating individual preferences to enhance adherence. - Given the anti-inflammatory, antioxidant, and neuroprotective properties, further research is warranted to explore the potential antidepressant effects of the MedDiet in older populations.
Selvaraj et al. (85)	SR	Systematic reviews, Cross-sectional studies, Cohort studies, Meta-analysis	9	MedDiet (3); DASH Diet (1)	- Only full-text peer-reviewed articles available for free. - Considered research publications from 2010 to 2021. - English language articles with human research involving healthy individuals or those with chronic/acute illnesses, including teenagers, adults, and the elderly. - Articles evaluating mental health and those focusing on depression or indications of depression.	Depression, anxiety	N/A	Not Available	Key ingredients of the MedDiet are crucial, with poorer diets associated with depression/anxiety diagnosis and more severe symptoms. While total calorie consumption linked to more severe anxiety and depression symptoms, individual food groups had modest impact on depression for patients but could be clinically significant for populations. Diverse food groups, particularly evident in the MedDiet score, appear linked to mental health.	- Conduct additional research to identify specific nutritional factors linked to depression prevention. - Execute prospective cohort studies and randomized controlled trials to thoroughly investigate the diet-depression connection. - Anticipate outcomes such as evidence-based policymaking and recognition of positive mental health benefits as reasons to prioritize a healthy diet.
Shafiei et al. (97)	SR/MA	Systematic Review (19) Meta-Analysis (14)	35	MedDiet (19)	- Population: Adults (18 years and older) - Intervention: MedDiet - Comparator: Highest vs. lowest adherence to MedDiet - Outcome: Depression - Setting: Cohort and cross-sectional studies Eligible studies met the following criteria: (1) Observational studies examining MedDiet as exposure and depression as outcome. Participants aged 18 years and older. (2) Reported effect sizes such as odds ratios, hazard ratios, or β estimates.	Depression	Hazard ratio for combined effect sizes from 4 studies, measuring the association between adherence to the MedDiet and risk of depression: 0.95 (95% CI: 0.79–1.16) No significant between-study heterogeneity (I2 = 45.2%, heterogeneity = 0.12) No evidence of publication bias (Egger test = 0.81) B coefficient for combined effect sizes from 3 studies, measuring the association between the MedDiet score and the depression score: 0.02 (95% CI: 0.118–0.113) No significant between-study heterogeneity (I2 = 0.0%, *p* = 0.99) Odds ratio for combined effect sizes from 9 studies, examining the association between adherence to the MedDiet and odds of depression: 0.72 (95% CI: 0.60–0.87) No significant between-study heterogeneity (I2 = 45.1%, Pheterogeneity = 0.068) No evidence of publication bias (Egger test = 0.39)	Adults (≥18 y)	The systematic review and updated meta-analysis found no significant link between adherence to the MedDiet and depression risk in cohort studies. However, cross-sectional studies demonstrated a significant inverse association between MedDiet adherence and depression odds.	- Clarify cutoff points for the MedDiet score to improve consistency. - Utilize longitudinal studies to establish causal relationships between diet and outcomes. - Address confounding factors to minimize bias in future research.
Solomou et al. (88)	SR	Cross-sectional, longitudinal	44	Diet inflammatory score (1), DASH (2), Energy-adjusted Dietary Inflammatory Index (2), MedDiet (5)	- Review included studies from peer-reviewed journals and gray literature, encompassing cross-sectional, longitudinal, and review studies of observational evidence. - Focused on exploring diet quality associations with mental health in university students through observational studies in natural settings. - Studies in English, Greek, and Spanish, with no publication period restrictions.	Depression, anxiety, stress	N/A	University students of any ethnic origin, gender and age, studying in any country, with or without a mental health diagnosis (such as depression and/or anxiety).	Studies found that test anxiety and academic stress negatively affected adherence to the MedDiet. Observational evidence from all studies suggests that a healthy diet among university students is associated with better	- Conduct additional observational studies and randomized control trials to assess interventions for promoting healthy diets and mental health among students. - Determine if interventions targeting diet quality in university settings can mitigate mental health issues.
Suschana et al. (89)	SR	Randomized Controlled Trials (RCTs), Controlled Clinical Trials (CCTs)	22	DASH diet or structured anti inflammatory dietary advice (1); Fatty acid and omega 3 based supplementation (6); Vitamin D supplementation alone or in combination (4); B vitamin supplementation including folate and vitamin B6 (3); Mineral supplementation such as magnesium (1); Probiotic or gut focused nutritional intervention (1); Other nutrition related adjunct interventions (1).	Adults diagnosed with schizophrenia and/or schizoaffective disorder; studies assessing dietary interventions targeting inflammatory or metabolic outcomes	Metabolic markers (CRP, IL-6, TNF-α, lipid profile, fasting glucose); psychiatric symptom remission (PANSS scores, cognitive performance)	Moderate pooled reduction in CRP (−0.26 mg/L; 95% CI: −0.45 to −0.07) and improvement in PANSS total score (SMD − 0.34; 95% CI: −0.52 to −0.16)	Adults (≥18 y) with schizophrenia and comorbid metabolic syndrome	Anti-inflammatory diets and supplementation demonstrate significant improvements in systemic inflammation and modest symptom remission in schizophrenia, highlighting the potential for integrative nutritional psychiatry	Future studies should employ larger, longitudinal RCTs, stratify participants by metabolic risk, and evaluate synergistic effects of diet and pharmacotherapy for sustained remission outcomes.
Tan et al. (90)	SR	Observational (cross-sectional, longitudinal), RCTs	16	DASH Diet (16)	- Intervention groups focused on the DASH diet or adherence score. - Measurement of at least 1 mental well-being outcome. - Observational or interventional trials - Conducted in humans.	Depression, emotion and mood	N/A	- Study sample sizes varied widely, ranging from 36 to 19,270 participants. - The majority of studies were from European countries (37.5%), followed by Iran (31.25%), United States (18.74%), Israel (6.25%), and Australia (6.25%).	The DASH diet appears to have potential benefits for mental well-being, although findings vary among studies, possibly due to variations in assessment methods for both the DASH diet and mental health outcomes.	- More research is needed on the DASH diet and psychological well-being due to limited literature. - The relationship between the DASH diet and depression is inconsistent; more studies are required to examine different levels of DASH diet adherence and depressive symptoms. - Quasi-experimental designs may be necessary for assigning adherence as a variable in future studies. - Consistent measures of depressive symptoms and DASH diet adherence are recommended. - More RCTs are needed to investigate these outcomes among individuals with mental health concerns, addressing a notable gap in the literature. - Longer follow-up durations in RCTs can provide insights into the long-term effects of the DASH diet on mental health outcomes. - Future studies should use rigorous active control groups to facilitate blinding and minimize bias. - Sophisticated causal inference methods can help address confounding variables in observational studies. - A priori sample size analyses should predict high dropout rates to ensure statistical power. - Strategies to maintain participant engagement throughout the study period should be incorporated. - Recruitment of representative study populations is essential for greater generalizability of findings. - Careful selection and interpretation of DASH diet indices are needed to ensure consistency and standardization across studies.
Tavakoly et al. (98)	SR/MA	RCTs: parallel (4), factorial (1), crossover, cluster	5	MedDiet (5)	- Population: Patients with a clinical diagnosis of any type of depressive disorders according to the Diagnostic and Statistical Manual of Mental Disorders (DSM)-V and the International Classification of Diseases (ICD)-11 - Intervention: Dietary patterns were defined based on the Dietary Guidelines Advisory Committee 2020, and the names of various dietary patterns were sourced from this reference as well as through additional literature searches to complete the list. - Comparison: Any type of control group, including both active and passive conditions, was considered - Outcome: Primary outcome was change in depressive symptoms severity, and secondary outcomes were changes in remission rate, health-related quality of life, and safety. - RCTs (parallel, crossover, cluster)	Depression	N/A	Adults (≥18 y); all genders and ethnicities; clinical diagnosis of depressive disorder via physician/trained professional, validated questionnaire, or prescribed antidepressant; any depressive disorder type (excluding criteria below); no restrictions on sociodemographic characteristics or ongoing pharmacologic/psychologic treatments	MD interventions appear to have no potential influence on major depressive disorder; however, this finding should be interpreted with caution due to the small number of RCTs available.	To draw more definitive conclusions, additional RCTs exploring various dietary patterns and their effects on depressive disorders are needed.
Tolkien et al. (99)	SR/MA	Longitudinal (7); Cross-Sectional (4)	11	Anti-Inflammatory Diet (11)	- Measured the inflammatory potential of the diet. - Measured the incidence of depression or depressive symptoms. - Reported effect size and confidence intervals (CI) for the association between an inflammatory diet and depression.	Depression	A significant association between a pro-inflammatory diet and increased risk of depression diagnosis or symptoms was evident, relative to those on an anti-inflammatory diet (OR: 1.40, 95% confidence intervals: 1.21–1.62, P < 0.001).	Participants aged 16 -72 years at baseline	Adopting an anti-inflammatory diet may effectively reduce the risk and symptoms of depression, as pro-inflammatory diets are linked to a higher likelihood of depression diagnosis or depressive symptoms.	- Investigate the link between diet quality and mental health, focusing on anti-inflammatory diets like the Mediterranean Diet (MedDiet). - Explore how pro-inflammatory diets contribute to depressive symptoms and their role in chronic diseases. - Use the Dietary Inflammatory Index (DII) to evaluate the inflammatory potential of diets and its clinical relevance. - Conduct randomized controlled trials (RCTs) to examine the effects of dietary interventions on mental health. - Include measures of dietary inflammatory potential and diverse dietary groups (pro-inflammatory, anti-inflammatory, and moderate) in future RCTs. - Address reverse causation to understand the bidirectional relationship between diet and depression.
Wang et al. (100)	SR/MA	Cohort (4) Cross-Sectional (2)	6	Anti-Inflammatory diet (6)	- Observational study design (cohort, case–control, or cross-sectional). - Inflammatory potential assessed using DII score. - Outcome measure: incident depression or depressive symptoms. - Reported multivariable-adjusted hazard ratio or odds ratio with confidence intervals for highest DII score vs. lowest DII score.	Depression	RR was 1·23 (95% CI 1·12, 1·35) for the highest v. the lowest DII score in a fixed-effects model, with no evidence of heterogeneity (I2 = 0·0%, P = 0·853). Sensitivity analysis indicated that no single study affected the pooled risk estimate significantly. When stratified by study design, the pooled RR was 1·25 (95% CI 1·12, 1·40) for the prospective cohort studies(,16,17,19,26) and 1·16 (95% CI 0·96, 1·41) for the cross-sectional studies	- Studies conducted in the USA, Spain, France, Ireland, and Australia. - Sample sizes ranged from 2,047 to 18,875, totaling 49,584 individuals.	The meta-analysis revealed that individuals with the highest pro-inflammatory diet scores had a 23% increased likelihood of experiencing depression compared to those with the lowest scores. This association held true across various study designs, with no significant variation between them. These findings emphasize the importance of reducing pro-inflammatory diets and advocating for anti-inflammatory diets to help lower the risk of depression, particularly among women.	- Further longitudinal studies are needed to clarify the gender-specific inflammatory potential of the diet-depression relationship. - Well-designed interventional trials are necessary to investigate the efficacy of anti-inflammatory diets for managing depression.
Zupo et al. (103)	UMR	Systematic review articles (with or without meta-analysis)	84	MedDiet (84)	- Systematic review articles with or without meta-analysis. - Clinical trials and observational studies included. - Publication time interval: 1990–2023. - Language: English.	Depression	N/A	N/A (not specified)	MedDiet consistently shows a reduced risk of depression. Lassale et al. and other studies support this, finding a relative risk estimate of 0.67 for high versus low adherence to the MedDiet in longitudinal studies. Lower dietary inflammatory index is also linked to reduced depression risk, with a relative risk of 0.76 (95% CI 0.63–0.92) in four longitudinal studies.	- Recommend adopting a Front-of-Package (FOP) label, such as Med-Index, to promote healthier consumer behavior. - Advocate for the use of FOP labels to encourage mindful food choices based on nutritional information. - Emphasize the potential of Med-Index to guide consumers toward healthier options.

*Study design counts are reported as described in the original reviews. Categories may overlap (e.g., cohort studies are typically longitudinal), and totals should not be interpreted as counts of unique primary studies. **Only relevant interventions listed. ***Only reported if the study performed its own metanalysis.

## 3 Results

**Figure 1:**
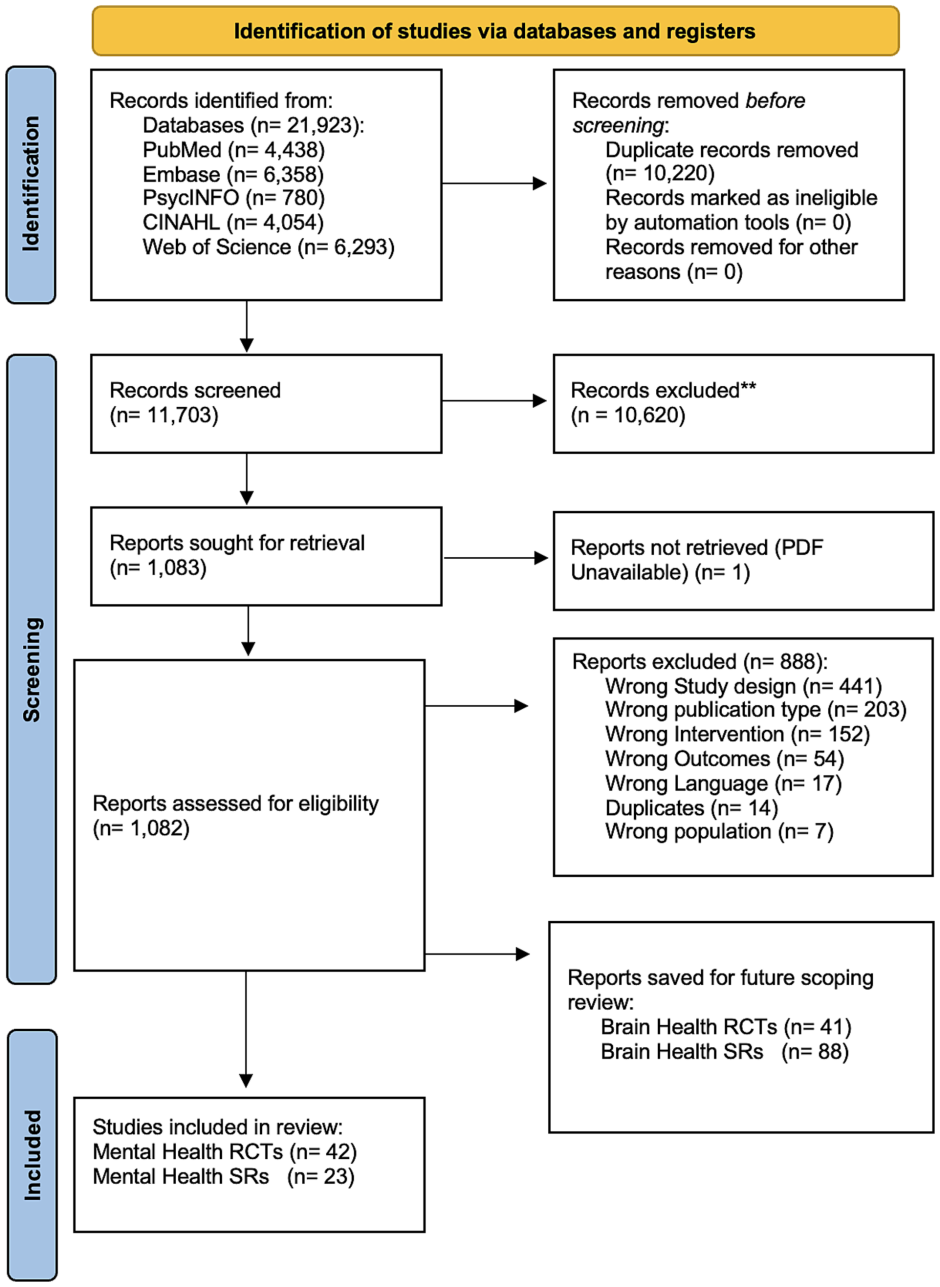
PRISMA diagram.

The search retrieved 11,703 citations after duplicates were removed. A total of 1,082 citations passed the initial screening, and the full text of these articles was reviewed during the secondary screening. Following both rounds of screening a total of 65 articles met the inclusion criteria: 42 RCTs and 23 systematic evidence syntheses (SRs/SRs + MAs/UMRs) (see Figure 1).

### 3.1 Clinical trial characteristics

Forty-two RCTs assessing the impact of anti-inflammatory diets on mental health outcomes were identified for inclusion (18, 20, 26, 29, 31, 33, 49–84). Studies were published between 2020 and 2025 and examined the effects of introducing anti-inflammatory dietary patterns that differed from participants’ typical diets, such as increasing intake of antioxidant-rich foods and reducing consumption of foods high in saturated fats.

**Figure 2:**
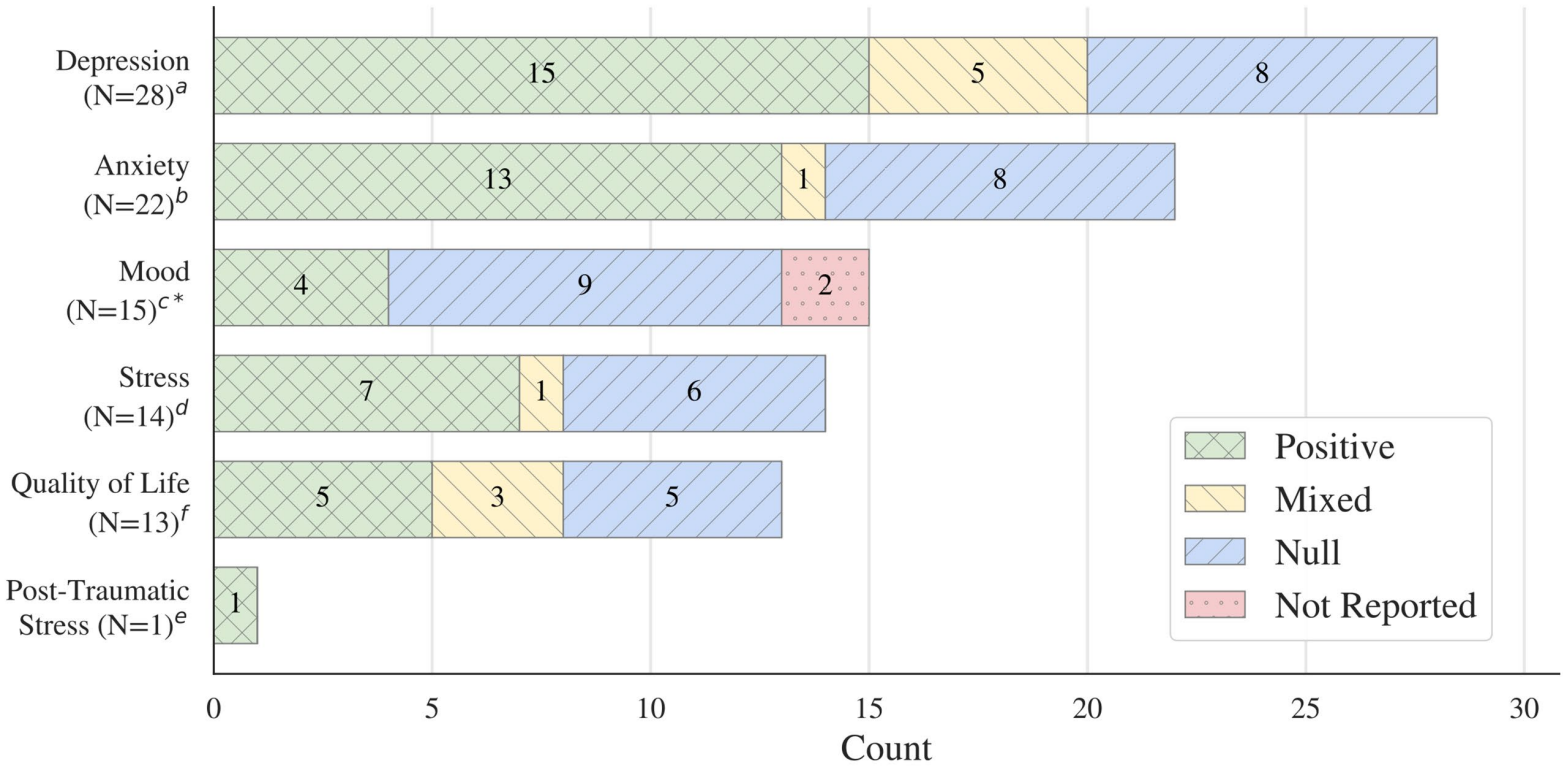
Distribution of studies across mental health outcomes and reported results. (a) Depression: positive (31, 33, 49, 52–54, 56–59, 61, 63, 65, 67, 73, 74), mixed (20, 55, 62, 64, 75), null (26, 60, 66, 69, 76–78, 82); (b) Anxiety: positive (31, 33, 51, 54, 56–61, 65, 67, 73), mixed (75), null (60, 63, 64, 66, 76, 77, 82, 84); (c) Mood: positive (18, 29, 79), null (61, 63–65, 70, 72, 80, 82, 84), not reported (66, 71); (d) Stress: positive (31, 33, 56, 58, 61, 64, 69), mixed (75), null (63, 66, 74, 78, 82, 83); (e) Quality of Life: positive (52, 64, 67, 72, 74), mixed (53, 80, 81), null (51, 59, 60, 66, 78); (f) Post-Traumatic Stress: positive (50). *These numbers reflect the total mood score form the POMS; specific dimensions of the POMS are not.

Mental health outcomes assessed included depression (*n* = 28), anxiety (*n* = 22), mood (*n* = 15), stress (*n* = 14), and post-traumatic stress (*n* = 1). Fourteen of the 42 studies also assessed quality of life. These results are summarized in Figure 2.

### 3.2 Dietary intervention characteristics and outcomes

There was variability in the types and characteristics of the anti-inflammatory dietary interventions employed to address mental health outcomes. Out of the 42 studies, 28 examined the effects of MedDiet (including variations) on mental health outcomes (17, 18, 31, 50–53, 56, 59–62, 64, 65, 68, 70–72, 74–77, 79–84). Seven studies focused on MedDiet interventions that incorporated specific components, such as nuts, extra-virgin olive oil, pork, or dairy (17, 33, 72, 77, 79–81), while others (*n* = 3) explored the effects of addition of supplements or substitutions of fats (53, 54, 60). Of the studies examining the MedDiet, eight of the 28 studies were multimodal, with studies incorporating nutritional education or counseling, social support, cooking classes, exercise or movement, stress management, smoking cessation support, mindfulness, forest bathing, and therapy (29, 31, 50, 51, 64, 65, 76, 78).

Moreover, five studies assessed the impact of DASH diet interventions (18, 58, 66, 79, 83), three studies explored the effects of the MIND Diet (a combination of the MedDiet and the DASH diet) (26, 29, 55), and one study examined the effects of the Nordic Diet (26). Four studies investigated unspecified bespoke anti-inflammatory diets (49, 53, 59, 69).

### 3.3 Population

The included studies investigated a variety of populations and patient groups. Ten studies specifically examined the effects of anti-inflammatory diets on individuals diagnosed with depressive disorders (*n* = 6) (26, 52, 55, 62, 65, 74), anxiety disorders (*n* = 1) (51), those reporting symptoms of depression or anxiety through self-report measures (*n* = 2) (52, 71), or individuals referred to a psychiatric clinic (*n* = 1) (75).

The remaining 32 studies focused on diverse populations, including healthy and older adults (*n* = 6) (53, 56, 64, 68, 70, 71), overweight or obese adults (*n* = 6) (50, 59, 67, 69, 72, 76), adults with metabolic disorders (n = 5) (29, 58, 63, 66, 78), individuals at risk for or diagnosed with cardiovascular disease (*n* = 4) (77, 80–82), those with elevated blood pressure or hypertension (*n* = 4) (18, 79, 83, 84), pregnant women (*n* = 3) (33, 54, 73), subjects with spinal cord injury (*n* = 2) (20, 49), people with neurodegenerative disease (*n* = 1) (60), and individuals with chronic pain, specifically fibromyalgia (*n* = 1).

### 3.4 Clinical trial results

Of the 42 studies included (Table 3), 28 reported significantly greater improvements in at least one mental health outcome compared to the control group following adherence to either a MedDiet style or another anti-inflammatory dietary intervention (Table 1) (17, 18, 29, 31, 33, 49–54, 56–61, 63–65, 67–70, 73–75, 79, 81, 85).

#### 3.4.1 Depression outcomes

Anti-inflammatory diets demonstrated positive or mixed effects on depression in 20 of 28 studies, with eight studies reporting null findings (Figure 2).

Effect sizes for depression outcomes were reported in 6 studies, ranging from small effects in two studies (58, 64), medium effects in two studies (56, 61), and large effects in two studies (49, 65).

#### 3.4.2 Anxiety outcomes

Anti-inflammatory diets demonstrated positive or mixed effects on anxiety in 14 of 22 studies assessing anxiety, with eight studies reporting null findings (Figure 2). Effect sizes for anxiety outcomes were reported in six studies, ranging from three studies with small effects (57, 58, 74), two studies with medium effects (61, 65) and one study reporting large effects (65).

#### 3.4.3 Mood outcomes

Mood and related features were assessed in 15 studies; however, two of these studies did not report total mood outcomes (Figure 2). Of these, four studies reported positive or mixed effects and nine reported null findings (Figure 2). Statistically significant changes in mood subscales were infrequent and largely confined to isolated studies, including findings for depression (*n* = 3) (66, 79, 81), tension (*n* = 2) (79, 81), vigor (*n* = 2) (79, 81), fatigue (*n* = 1) (66), and anger (*n* = 1) (81), while the majority of studies assessing these domains reported null results. When reported, effect sizes ranged from very small to large, with two reporting small effect sizes (66, 84), and one reporting single large effect sizes (81).

#### 3.4.4 Stress outcomes

Stress was evaluated in 14 studies, with eight reporting positive or mixed effects and six reporting null fundings (Figure 2). One study reported a large effect size (61).

#### 3.4.5 Post-traumatic stress outcomes

One study reported positive effects of an anti-inflammatory diet on PTSD outcomes, though effect size data were not provided (Figure 2).

#### 3.4.6 Quality of life outcomes

Among 14 studies assessing quality of life, eight reported positive or mixed effects, with eight reporting null findings (Figure 2). Effect sizes were not reported for quality-of-life outcomes.

#### 3.4.7 Populations with diagnosed or indicated for mental health conditions

Ten articles in total evaluated anti-inflammatory diets specifically for populations either diagnosed with or indicated for mental health conditions (17, 26, 31, 51, 52, 61, 62, 65, 74, 75). Of the nine articles which evaluated anti-inflammatory diets specifically for populations diagnosed with depression and related disorders, six of the nine studies demonstrated positive results (17, 31, 52, 61, 65, 74), and two demonstrated mixed results (62, 75), and one reported null results (26). For the three studies that confirmed participants depressive symptom levels at the start of the study, two reported positive results (61, 65), and one reported null findings (26). For the three studies where effect size was calculated, two reported medium effect sizes (≥ 0.5) (61, 75) and one reported a large effect size (65). In the five studies examining anxiety related outcomes in participants either diagnosed with or reporting depression symptoms, three were positive (31, 61, 65), one was mixed (75), and one was null (74).

Of the three studies in which participants were either diagnosed with an anxiety disorder or referred to a psychiatric clinic for anxiety-related concerns, two demonstrated positive results (31, 51), and one demonstrated mixed results (75).

### 3.5 Systematic reviews, meta-analyses, and scoping/umbrella review results

To contextualize the results for anti-inflammatory dietary patterns and mental health outcomes, pooled evidence from reviews including a range of study designs, beyond RCTs, was examined. In total, 23 systematic evidence syntheses were identified, comprising of SRs (*N* = 10) (85–94), SRs + MAs (*N* = 10) (91–100), and UMRs (*N* = 3) (101–103). These reviews synthesized findings from observational studies (*N* = 16) (85, 87, 88, 90, 93–95, 97, 99–102, 104–107), intervention studies (*N* = 5) (90, 92, 102, 104, 106), RCTs (*N* = 5) (86, 89, 91, 98, 101), and prior systematic reviews (*N* = 2) (85, 103).

Across these reviews, anti-inflammatory dietary patterns were most commonly operationalized through measures of adherence to the MedDiet (*N* = 18) (85–88, 91–98, 101–105, 107), with fewer reviews examining other dietary patterns such as the Nordic (*N* = 2) (85, 106), or DASH diets (*N* = 7) (85, 88–90, 93, 102, 105). Dietary exposure was typically assessed using self-reported instruments reflecting habitual intake rather than prescribed dietary interventions. Depression (*N* = 21) (85–88, 90, 91, 93–107), was the most frequently assessed mental health outcome, followed by anxiety (*N* = 5) (85, 88, 105–107).

#### 3.5.1 Depression

##### 3.5.1.1 MedDiet

Across the 23 SRs, SRs + with MAs, and UMR evaluating the MedDiet, findings related to depression outcomes were heterogeneous (85, 87–107). Several reviews synthesizing primarily observational evidence reported inverse associations between MedDiet adherence and depression outcomes (*N* = 11) (85, 87, 88, 93, 95, 97, 101, 102, 104, 105, 107). In the majority of these reviews, dietary exposure was assessed using self-reported adherence indices that captured habitual dietary patterns rather than controlled or prescribed dietary interventions (*N* = 18) (85, 87, 88, 90, 93–97, 99–107). In a smaller subset of reviews, dietary exposure was evaluated through RCTs or other intervention designs in which the Mediterranean Diet was actively prescribed, supported, or monitored (*N* = 5) (86, 89, 91, 92, 98).

Eleven reviews reported lower depressive symptom scores or reduced depression prevalence among individuals with higher adherence to the MedDiet (86, 93, 95–97, 101–105, 107). These associations were observed primarily in reviews synthesizing observational studies. Findings from reviews of MedDiet RCTs were also mostly positive, with four reviews (86, 91, 92, 104) reporting improvements in depressive symptoms following dietary interventions and one review reporting no statistically significant effects (98).

Only one review pooled data exclusively from prospective cohort studies and reported no statistically significant association between higher versus lower Mediterranean Diet adherence and incident depression (94). Similarly, mixed findings were observed in a single review, with inverse associations identified in cross sectional analyses but not in longitudinal or cohort-based analyses (97).

Multiple reviews (*N* = 6) reported associations between pro-inflammatory dietary patterns and higher odds of depression or greater depressive symptom severity (93, 94, 99, 100, 102, 103). These dietary patterns included higher intakes of ultra-processed foods, refined carbohydrates, red and processed meats, and sodium. In contrast, pooled cross-sectional analyses within some reviews reported lower odds of depression associated with higher MedDiet adherence (*N* = 4) (93, 96, 97, 103).

##### 3.5.1.2 Nordic diet

One SR assessed the Nordic Diet in relation to depression outcomes. This review identified one small randomized controlled trial conducted in adults diagnosed with major depressive disorder (106). Participants assigned to a Nordic-style dietary pattern exhibited greater reductions in depressive symptom scores compared with participants in a control group over a short intervention period. No additional randomized controlled trials were identified.

##### 3.5.1.3 DASH diet

Two SRs (90, 105), one SR + MA (93), and one UMR (102), evaluated adherence to the DASH diet in relation to depression outcomes. Findings across these reviews were inconsistent. Some analyses reported associations between higher DASH adherence and measures of mental well-being, while others reported no statistically significant associations with depression risk or depressive symptom severity. Overall, pooled estimates did not demonstrate a consistent association between DASH diet adherence and depression outcomes across study designs.

#### 3.5.2 Anxiety

Five SRs (85, 88, 105–107) examined associations between anti-inflammatory dietary patterns and anxiety-related outcomes. Reported findings were heterogeneous. Evidence from cross-sectional studies (*n* = 4) described inverse associations between adherence to health-promoting dietary patterns, including Mediterranean and Nordic diets, and anxiety or stress scores (88, 105–107). Other reviews (*n* = 2) reported associations between poorer overall diet quality or higher intake of pro-inflammatory foods and greater anxiety symptom severity (85, 88).

All five reviews that examined anxiety outcomes reported inverse associations between adherence to anti-inflammatory dietary patterns and anxiety symptoms in some analyses, while also reporting null or inconclusive findings in others. Across these reviews, variability was observed in populations studied, dietary assessment methods, anxiety measures, and study designs.

#### 3.5.3 Other axis I disorders

One review (107) also examined the relationship between adherence to the MedDiet and the prevalence of other Axis I disorders beyond anxiety and depression, including bipolar disorder, schizophrenia and psychosis, PTSD, and eating disorders. Overall, the review reported inverse associations, with the most consistent findings observed for depression and anxiety, while evidence for other Axis I disorders was limited and heterogeneous.

## 4 Discussion

This scoping review synthesized evidence from 42 RCTs and 23 systematic evidence syntheses to examine the relationship between anti-inflammatory dietary patterns and mental health outcomes. Across both bodies of evidence, anti-inflammatory diets, particularly MedDiet-style patterns, were associated with beneficial or mixed effects on depression, anxiety, mood, stress, and quality of life. Findings from experimental trials and review-level analyses were largely convergent, suggesting that dietary patterns emphasizing whole, minimally processed foods may support improvements in mental health symptoms and psychological well-being, particularly among individuals with existing symptoms.

### 4.1 Integration of RCT and systematic evidence syntheses

Across the included literature, results from RCTs and systematic evidence syntheses were broadly aligned. Review-level evidence most consistently supported inverse associations between adherence to anti-inflammatory dietary patterns and depression risk or symptom severity, particularly for the MedDiet. The RCT evidence extended these findings by demonstrating that dietary interventions can produce measurable improvements in mental health outcomes, especially depressive symptoms. Improvements were most frequently observed in populations with clinically diagnosed depression or elevated depressive symptoms at baseline, suggesting that anti-inflammatory diets may have greater therapeutic relevance for individuals experiencing psychological distress rather than for asymptomatic populations (17, 31, 52, 61, 62, 65, 74).

Evidence for anxiety outcomes was more limited and heterogeneous. While several systematic evidence syntheses reported inverse associations between diet quality or adherence to anti-inflammatory dietary patterns and anxiety symptoms, findings were not uniform across study designs. Similarly, RCTs assessing anxiety outcomes reported a mix of positive, mixed, and null results. Mood, stress, and quality-of-life outcomes showed generally favorable trends across both evidence streams, though effects were less consistent than for depression and varied across populations, intervention designs, and outcome measures. Despite this variability, the overall alignment between trial-level and review-level findings strengthens confidence in the potential mental health benefits of anti-inflammatory dietary patterns.

### 4.2 Consistency and variability across diet types

The MedDiet was the most frequently studied dietary pattern and demonstrated the most consistent evidence for mental health benefits across both RCTs and systematic evidence syntheses, including SRs, SR + MAs, and UMRs (29, 31, 50–52, 54–57, 60–62, 64, 65, 68, 70–78, 80–82, 85–88, 91–98, 101–105, 107). However, positive or mixed findings were also observed for other anti-inflammatory dietary approaches, including the DASH diet, MIND diet, Nordic diet, and several unclassified whole-food or anti-inflammatory interventions (18, 49, 53, 58, 59, 63, 66, 67, 69, 79, 83, 84, 90, 99, 100, 106). Although these diets differ in specific components, they share common features such as high intake of fruits, vegetables, whole grains, and unsaturated fats, along with reduced consumption of ultra-processed foods. This overlap suggests that broader dietary principles related to inflammation reduction may be more important than adherence to a single prescribed dietary pattern.

### 4.3 Mechanisms underlying dietary effects on mental health

The combined evidence supports several biological pathways through which anti-inflammatory diets may influence mental health outcomes. Foods emphasized in anti-inflammatory dietary patterns provide nutrients associated with reduced systemic inflammation, modulation of inflammatory cytokines, and support for neurotransmitter synthesis and function. Omega-3 fatty acids, particularly eicosapentaenoic acid (EPA) and docosahexaenoic acid (DHA), reduce inflammatory activity by modulating prostaglandins and inflammatory cytokines and have been linked to increased serotonin availability, a neurotransmitter central to mood regulation, cognition, and emotional functioning (17, 26, 29, 36, 40, 46, 50, 55, 58, 60, 63, 72, 75, 76, 79, 85, 87, 113, 114). Vitamins and minerals commonly found in anti-inflammatory diets also serve as essential biochemical cofactors in serotonin synthesis and metabolism (108, 109). Supporting this pathway, prior work has shown that individuals with depressive disorders who adopt a Mediterranean-style diet exhibit increased concentrations of micronutrients involved in neurotransmitter synthesis and mood regulation (62).

Inflammation and chronic stress can disrupt these neurobiological pathways by impairing serotonin synthesis and increasing neuroinflammatory processes that affect emotional and cognitive functioning. Pro-inflammatory cytokines and enzymes have been shown to reduce serotonin availability, alter neurotransmitter metabolism, and promote neuroinflammation associated with depressive and anxiety-related symptoms (110–112). Cytokines may also cross the blood–brain barrier and activate immune signaling within the central nervous system, further contributing to dysregulated mood and behavior (9, 28, 30). Several RCTs included in this review reported associations between dietary intervention, reductions in inflammatory markers, and corresponding improvements in mood or depressive symptoms (49, 76, 81), lending support to these proposed mechanisms. However, not all studies measured inflammatory biomarkers, and improvements in mental health outcomes were sometimes observed in the absence of detectable biomarker changes, suggesting that additional psychological, behavioral, or metabolic mechanisms may also contribute.

### 4.4 Broader psychological and behavioral pathways

Some interventions produced improvements in mental health outcomes without clear changes in inflammatory markers (53, 61, 66, 68, 80, 81). These effects may reflect broader psychological or behavioral pathways, including improvements in overall nutrient quality, reductions in ultra-processed food intake, increased engagement in health-promoting behaviors, or enhanced self-efficacy related to dietary change. Many interventions included in this review were multimodal and incorporated components such as nutritional education, social support, cooking classes, physical activity, or stress management strategies (52, 69, 74, 76, 83, 84). While these intervention designs may enhance real-world applicability and adherence, they limit the ability to isolate the independent effects of dietary change on mental health outcomes.

### 4.5 Limitations

Several limitations of this review prevent stronger conclusions. It is not possible to assign directional causality in cross-sectional or longitudinal studies linking anti-inflammatory diet adherence to mental health outcomes, as depression and anxiety may influence eating patterns rather than dietary patterns influencing mental health. The dietary interventions included in the randomized controlled trials differed substantially in their composition and delivery, and many were multimodal, incorporating elements such as nutritional counseling, physical activity, or psychosocial support. This complexity makes it difficult to determine whether observed effects are attributable to dietary components themselves or to the combined influence of multiple intervention elements. Variation in intervention duration, participant characteristics, follow-up timing, and the specific foods emphasized further limited the comparability of outcomes across studies. Depression was the most frequently assessed outcome, with anxiety, mood, stress, and quality of life also examined; however, few trials evaluated other mental health domains such as PTS, and other Axis 1 disorders, which constrains conclusions in these areas. In addition, the review relied on previously synthesized evidence for the systematic review component, limiting the ability to draw direct inferences about the effects of anti-inflammatory diets on less-studied mental health outcomes. Finally, as this was a scoping review, no formal assessment of study quality was conducted, restricting the extent to which the strength and reliability of individual findings can be evaluated.

## 5 Conclusion

The findings of this review indicate that anti-inflammatory dietary patterns are associated with improvements in mental health outcomes. Across RCTs, SRs, SRs + MAs, and UMRs, anti-inflammatory dietary interventions were consistently associated with reduced depressive symptoms and lower depression risk, while several SRs + MAs also identified positive associations between pro-inflammatory dietary patterns and increased depression risk. Collectively, these findings support inflammation as a biological pathway linking diet and mental health and suggest that anti-inflammatory diets, particularly the MedDiet, may be associated with beneficial effects on mood and emotional regulation through reductions in inflammatory processes.

The strongest evidence emerged from RCTs conducted in populations with clinically diagnosed or elevated symptoms of depression or anxiety, where dietary interventions frequently demonstrated meaningful improvements in depression-related outcomes. Although these findings support an association between anti-inflammatory diets and reduced depressive symptoms and risk, additional well-designed RCTs are needed to strengthen causal inference and establish the efficacy of dietary interventions for mental health treatment.

Future research would benefit from a more coordinated agenda aimed at addressing current gaps in the literature. Standardized dietary interventions, longer intervention durations with extended follow-up periods, and greater consistency in outcome measurement will be essential for improving comparability across studies. Replication of promising findings and studies designed to identify the specific dietary components responsible for observed effects will help clarify which anti-inflammatory dietary approaches are most effective for improving mental health outcomes. Rigorous and replicated evidence demonstrating the causal effectiveness of anti-inflammatory diets for depression and other mental health conditions will be critical for supporting their broader integration into mental health care.

## Footnotes

1 Quality of life outcomes were not classified as mental health outcomes and were excluded when reported as the sole outcome. When studies reported both quality of life and eligible outcomes, quality of life data were extracted.

## References

[1] Medzhitov R. Origin and physiological roles of inflammation. Nature. (2008) 454:428–35. doi: 10.1038/nature07201, 18650913

[2] Pahwa R Goyal A Jialal I. Chronic Inflammation. Treasure Island, FL: StatPearls Publishing (2025). 29630225

[3] Furman D Campisi J Verdin E Carrera-Bastos P Targ S Franceschi C . Chronic inflammation in the etiology of disease across the life span. Nat Med. (2019) 25:1822–32. doi: 10.1038/s41591-019-0675-0, 31806905 PMC7147972

[4] Makkar H Reynolds MA Wadhawan A Dagdag A Merchant AT Postolache TT. Periodontal, metabolic, and cardiovascular disease: exploring the role of inflammation and mental health. Pteridines. (2018) 29:124–63. doi: 10.1515/pteridines-2018-0013, 30705520 PMC6350811

[5] Minihane AM Vinoy S Russell WR Baka A Roche HM Tuohy KM . Low-grade inflammation, diet composition and health: current research evidence and its translation. Br J Nutr. (2015) 114:999–1012. doi: 10.1017/S0007114515002093, 26228057 PMC4579563

[6] Gialluisi A Bonaccio M Di Castelnuovo A Costanzo S De Curtis A Sarchiapone M . Lifestyle and biological factors influence the relationship between mental health and low-grade inflammation. Brain Behav Immun. (2020) 85:31055172:4–13. doi: 10.1016/j.bbi.2019.04.041 31055172

[7] HepsomaliP CoxonC. Inflammation and diet: focus on mental and cognitive health. Adv Clin Exp Med. (2022) 31:821–5. doi: 10.17219/acem/152350, 35951624

[8] Kim JR Kim HN Song SW. Associations among inflammation, mental health, and quality of life in adults with metabolic syndrome. Diabetol Metab Syndr. (2018) 10:66. doi: 10.1186/s13098-018-0367-9, 30186371 PMC6119265

[9] Lee CH Giuliani F. The role of inflammation in depression and fatigue. Front Immunol. (2019) 10:1696. doi: 10.3389/fimmu.2019.01696, 31379879 PMC6658985

[10] Upthegrove R Khandaker GM. "Cytokines, Oxidative Stress and Cellular Markers of Inflammation in Schizophrenia". In: Khandaker GM Meyer U Jones PB, editors. Neuroinflammation and Schizophrenia. Cham: Springer International Publishing (2019)

[11] Khandaker GM Dantzer R Jones PB. Immunopsychiatry: important facts. Psychol Med. (2017) 47:2229–37. doi: 10.1017/S0033291717000745, 28418288 PMC5817424

[12] Naudé PJW Roest AM Stein DJ De Jonge P Doornbos B. Anxiety disorders and CRP in a population cohort study with 54,326 participants: the LifeLines study. World J Biol Psychiatry. (2018) 19:461–70. doi: 10.1080/15622975.2018.1433325, 29376460

[13] Osimo EF Baxter LJ Lewis G Jones PB Khandaker GM. Prevalence of low-grade inflammation in depression: a systematic review and meta-analysis of CRP levels. Psychol Med. (2019) 49:1958–70. doi: 10.1017/S0033291719001454, 31258105 PMC6712955

[14] Phillips CM Shivappa N Hébert JR Perry IJ. Dietary inflammatory index and mental health: a cross-sectional analysis of the relationship with depressive symptoms, anxiety and well-being in adults. Clin Nutr. (2018) 37:1485–91. doi: 10.1016/j.clnu.2017.08.029, 28912008

[15] Tayefi M Shafiee M Kazemi-Bajestani SMR Esmaeili H Darroudi S Khakpouri S . Depression and anxiety both associate with serum level of hs-CRP: a gender-stratified analysis in a population-based study. Psychoneuroendocrinology. (2017) 81:63–9. doi: 10.1016/j.psyneuen.2017.02.035, 28419913

[16] Lasselin J Capuron L. Chronic low-grade inflammation in metabolic disorders: relevance for behavioral symptoms. Neuroimmunomodulation. (2014) 21:95–101. doi: 10.1159/000356535, 24557041

[17] Cabrera-Suárez BM Lahortiga-Ramos F Sayon-Orea C Hernández-Fleta JL González-Pinto A Molero P . Effect of a dietary intervention based on the Mediterranean diet on the quality of life of patients recovered from depression: analysis of the PREDIDEP randomized trial. Exp Gerontol. (2023) 175:112149. doi: 10.1016/j.exger.2023.112149, 36933773

[18] Torres SJ Nowson CA. A moderate-sodium DASH-type diet improves mood in postmenopausal women. Nutrition. (2012) 28:896–900. doi: 10.1016/j.nut.2011.11.029, 22480799

[19] Uher R Tansey KE Dew T Maier W Mors O Hauser J . An inflammatory biomarker as a differential predictor of outcome of depression treatment with escitalopram and nortriptyline. Am J Psychiatry. (2014) 171:1278–86. doi: 10.1176/appi.ajp.2014.14010094, 25017001

[20] Gazzellone G Lanteigne S Gammage K Fajardo VA Ditor DS. The effects of an anti-inflammatory dietary consultation on self-efficacy, adherence and selected health outcomes: a randomized control trial. Am J Lifestyle Med. (2023):15598276231215271. doi: 10.1177/15598276231215271 39554960;, 39554960 PMC11562215

[21] Serrats J Grigoleit JS Alvarez-Salas E Sawchenko PE. Pro-inflammatory immune-to-brain signaling is involved in neuroendocrine responses to acute emotional stress. Brain Behav Immun. (2017) 62:53–63. doi: 10.1016/j.bbi.2017.02.003, 28179107

[22] Esch T Stefano GB Fricchione GL Benson H. The role of stress in neurodegenerative diseases and mental disorders. Neuro Endocrinol Lett. (2002) 23:12080279:199–208. 12080279

[23] Liu YZ Wang YX Jiang CL. Inflammation: the common pathway of stress-related diseases. Front Hum Neurosci. (2017) 11:316. doi: 10.3389/fnhum.2017.00316, 28676747 PMC5476783

[24] Maydych V. The interplay between stress, inflammation, and emotional attention: relevance for depression. Front Neurosci. (2019) 13:384. doi: 10.3389/fnins.2019.00384, 31068783 PMC6491771

[25] Rohleder N. Stress and inflammation – the need to address the gap in the transition between acute and chronic stress effects. Psychoneuroendocrinology. (2019) 105:164–71. doi: 10.1016/j.psyneuen.2019.02.021, 30826163

[26] Sabet JA Ekman MS Lundvall AS Risérus U Johansson U Öström Å . Feasibility and acceptability of a healthy Nordic diet intervention for the treatment of depression: a randomized controlled pilot trial. Nutrients. (2021) 13:902. doi: 10.3390/nu13030902 33802181, 33802181 PMC7999972

[27] Berk M Williams LJ Jacka FN O’Neil A Pasco JA Moylan S . So depression is an inflammatory disease, but where does the inflammation come from? BMC Med. (2013) 11:200. doi: 10.1186/1741-7015-11-200, 24228900 PMC3846682

[28] Dantzer R O’Connor JC Freund GG Johnson RW Kelley KW. From inflammation to sickness and depression: when the immune system subjugates the brain. Nat Rev Neurosci. (2008) 9:46–56. doi: 10.1038/nrn2297, 18073775 PMC2919277

[29] Diekmann C Wagner M Huber H Preuß M Preuß P Predel HG . Acute impact of dietary pattern and walking on postprandial attention, mood, and satiety in older adults: a randomized crossover trial. Nutrients. (2019) 11:2294. doi: 10.3390/nu11102294, 31561490 PMC6835356

[30] Raison CL Capuron L Miller AH. Cytokines sing the blues: inflammation and the pathogenesis of depression. Trends Immunol. (2006) 27:24–31. doi: 10.1016/j.it.2005.11.006, 16316783 PMC3392963

[31] Staudacher HM Mahoney S Canale K Opie RS Loughman A So D . Clinical trial: a Mediterranean diet is feasible and improves gastrointestinal and psychological symptoms in irritable bowel syndrome. Aliment Pharmacol Ther. (2024) 59:492–503. doi: 10.1111/apt.17791 PubMed 37969059, 37969059

[32] Beurel E Toups M Nemeroff CB. The bidirectional relationship of depression and inflammation: double trouble. Neuron. (2020) 107:234–56. doi: 10.1016/j.neuron.2020.06.002, 32553197 PMC7381373

[33] Casas I Nakaki A Pascal R Castro-Barquero S Youssef L Genero M . Effects of a mediterranean diet intervention on maternal stress, well-being, and sleep quality throughout gestation—the IMPACT-BCN trial. Nutrients. (2023) 15:2362. doi: 10.3390/nu15102362, 37242244 PMC10223296

[34] Sakhaei R Shahvazi S Mozaffari-Khosravi H Samadi M Khatibi N Nadjarzadeh A . The dietary approaches to stop hypertension (DASH)-style diet and an alternative Mediterranean diet are differently associated with serum inflammatory markers in female adults. Food Nutr Bull. (2018) 39:361–76. doi: 10.1177/0379572118783950, 29969908

[35] Tsigalou C Konstantinidis T Paraschaki A Stavropoulou E Voidarou C Bezirtzoglou E. Mediterranean diet as a tool to combat inflammation and chronic diseases. An overview. Biomedicine. (2020) 8:201. doi: 10.3390/biomedicines8070201, 32650619 PMC7400632

[36] Yu X Pu H Voss M. Overview of anti-inflammatory diets and their promising effects on non-communicable diseases. Br J Nutr. (2024) 132:898–918. doi: 10.1017/S0007114524001405, 39411832 PMC11576095

[37] Bremner J Moazzami K Wittbrodt M Nye J Lima B Gillespie C . Diet, stress and mental health. Nutrients. (2020) 12:2428. doi: 10.3390/nu12082428, 32823562 PMC7468813

[38] Edirappuli SD Venkatesh A Zaman R. The effect of nutrition on mental health: a focus on inflammatory mechanisms. Psychiatr Danub. (2020) 32:114–20. 32890373

[39] Azzam A. Is the world converging to a ‘Western diet’? Public Health Nutr. (2021) 24:309–17. doi: 10.1017/S136898002000350X, 33077024 PMC10195558

[40] Mukherjee MS Han CY Sukumaran S Delaney CL Miller MD. Effect of anti-inflammatory diets on inflammation markers in adult human populations: a systematic review of randomized controlled trials. Nutr Rev. (2022) 81:55–74. doi: 10.1093/nutrit/nuac045, 35831971

[41] Cena H Calder PC. Defining a healthy diet: evidence for the role of contemporary dietary patterns in health and disease. Nutrients. (2020) 12:334. doi: 10.3390/nu12020334, 32012681 PMC7071223

[42] Fatima G Dalmadi I Süllős G Takács K Halmy E. Dietary patterns for health-span and longevity: a comprehensive review of nutritional strategies promoting lifelong wellness. Appl Sci. (2025) 15:12013. doi: 10.3390/app152212013

[43] Pes GM Dore MP Tsofliou F Poulain M. Diet and longevity in the blue zones: a set-and-forget issue? Maturitas. (2022) 164:31–7. doi: 10.1016/j.maturitas.2022.06.004, 35780634

[44] Pes GM Poulain M Errigo A Dore MP. Evolution of the dietary patterns across nutrition transition in the Sardinian longevity blue zone and association with health indicators in the oldest old. Nutrients. (2021) 13:1495. doi: 10.3390/nu13051495, 33925037 PMC8146058

[45] Ruiu M Carta V Deiana C Fastame MC. Is the Sardinian blue zone the new Shangri-La for mental health? Evidence on depressive symptoms and its correlates in late adult life span. Aging Clin Exp Res. (2022) 34:1315–22. doi: 10.1007/s40520-021-02068-7 35084663, 35084663 PMC9151576

[46] Tricco AC Lillie E Zarin W O’Brien KK Colquhoun H Levac D . PRISMA extension for scoping reviews (PRISMA-ScR): checklist and explanation. Ann Intern Med. (2018) 169:467–73. doi: 10.7326/M18-0850 30178033

[47] Grosso G Galvano F Marventano S Malaguarnera M Bucolo C Drago F . Omega-3 fatty acids and depression: scientific evidence and biological mechanisms. Oxidative Med Cell Longev. (2014) 2014:1–16. doi: 10.1155/2014/313570, 24757497 PMC3976923

[48] Sinn N Milte C Howe PRC. Oiling the brain: a review of randomized controlled trials of omega-3 fatty acids in psychopathology across the lifespan. Nutrients. (2010) 2:128–70. doi: 10.3390/nu2020128, 22254013 PMC3257637

[49] Allison DJ Ditor DS. Targeting inflammation to influence mood following spinal cord injury: a randomized clinical trial. J Neuroinflammation. (2015) 12:204. doi: 10.1186/s12974-015-0425-2, 26545369 PMC4636770

[50] Arcan C Hou W Hoffman K Reichardt A Yang X Clouston SAP . Mediterranean diet intervention among world trade center responders with post-traumatic stress disorder: feasibility and outcomes of a pilot randomized controlled trial. Obes Sci Pract. (2024) 10:e725. doi: 10.1002/osp4.725, 38263989 PMC10804354

[51] Aucoin M LaChance L Van Der Wurff I Miller S Naidoo U Jenkins A . Dietary counselling plus omega-3 supplementation in the treatment of generalized anxiety disorder: protocol for a randomized wait-list controlled pilot trial (the “EASe-GAD trial”). Pilot Feasibility Stud. (2023) 9:186. doi: 10.1186/s40814-023-01414-y, 37950301 PMC10636887

[52] Bayes J Schloss J Sibbritt D. The effect of a Mediterranean diet on the symptoms of depression in young males (the “AMMEND: a Mediterranean diet in MEN with depression” study): a randomized controlled trial. Am J Clin Nutr. (2022) 116:572–80. doi: 10.1093/ajcn/nqac106, 35441666

[53] Bourdel-Marchasson I Ostan R Regueme SC Pinto A Pryen F Charrouf Z . Quality of life: psychological symptoms—effects of a 2-month healthy diet and nutraceutical intervention; a randomized, open-label intervention trial (RISTOMED). Nutrients. (2020) 12:800. doi: 10.3390/nu12030800, 32197408 PMC7146172

[54] Bozdoğan FBK Kabaran S Tazeoğlu A. Effect of probiotic supplementation on maternal depression, anxiety and attachment in gestational diabetes by improving mediterranean diet quality: a randomized controlled trial. Clin Exp Obstet Gynecol. (2024) 51:237. doi: 10.31083/j.ceog5111237

[55] Cabrera-Suárez BM Hernández-Fleta JL Molero P González-Pinto A Lahortiga F Cabrera C . Mediterranean diet-based intervention to improve depressive symptoms: analysis of the PREDIDEP randomized trial. Nutr Neurosci. (2024) 27:951–61. doi: 10.1080/1028415X.2023.2283290, 37990906

[56] Carcelén-Fraile MDC Déniz-Ramírez NDP Sabina-Campos J Aibar-Almazán A Rivas-Campo Y González-Martín AM . Exercise and nutrition in the mental health of the older adult population: a randomized controlled clinical trial. Nutrients. (2024) 16:1741. doi: 10.3390/nu16111741, 38892674 PMC11174647

[57] Casini I Ladisa V Clemente L Delussi M Rostanzo E Peparini S . A personalized Mediterranean diet improves pain and quality of life in patients with fibromyalgia. Pain Ther. (2024) 13:609–20. doi: 10.1007/s40122-024-00598-2, 38683449 PMC11111631

[58] Daneshzad E Heshmati J Basirat V Keshavarz SA Qorbani M Larijani B . The effect of the dietary approaches to stop hypertension (DASH) diet on sleep, mental health, and hormonal changes: a randomized clinical trial in women with type 2 diabetes. Front Nutr. (2022) 9:775543. doi: 10.3389/fnut.2022.775543, 35634394 PMC9133887

[59] Dolatkhah N Toopchizadeh V Barmaki S Salekzamani Y Najjari A Farshbaf-Khalili A . The effect of an anti-inflammatory in comparison with a low caloric diet on physical and mental health in overweight and obese women with knee osteoarthritis: a randomized clinical trial. Eur J Nutr. (2022). doi: 10.1007/s00394-022-03017-4 36183308

[60] Felicetti F Ruggieri S Ruotolo I Livi C Sellitto G D’Ambrosi G . Improvement of measured and perceived disability in overweight patients with multiple sclerosis trough different patterns of Mediterranean hypocaloric diet. Mult Scler Relat Disord. (2025) 94:106271. doi: 10.1016/j.msard.2025.106271 39823692

[61] Francis HM Stevenson RJ Chambers JR Gupta D Newey B Lim CK. A brief diet intervention can reduce symptoms of depression in young adults – a randomised controlled trial. PLoS One. (2019) 14:e0222768. doi: 10.1371/journal.pone.0222768, 31596866 PMC6784975

[62] Ibarra O Gili M Roca M Vives M Serrano MJ Pareja A . The Mediterranean diet and micronutrient levels in depressive patients. Nutr Hosp. (2014) 31:1171–5. doi: 10.3305/nh.2015.31.3.8124, 25726209

[63] Golmohammadi M Attari VE Salimi Y Saed L Nachvak SM Samadi M. The effect of MIND diet on sleep status, mental health, and serum level of BDNF in overweight/obese diabetic women with insomnia: a randomized controlled trial. Sci Rep. (2025) 15:8237. doi: 10.1038/s41598-025-91389-y, 40065021 PMC11893750

[64] Hardman RJ Meyer D Kennedy G Macpherson H Scholey AB Pipingas A. Findings of a pilot study investigating the effects of Mediterranean diet and aerobic exercise on cognition in cognitively healthy older people living independently within aged-care facilities: the lifestyle intervention in independent living aged care (LIILAC) study. Curr Dev Nutr. (2020) 4:nzaa077. doi: 10.1093/cdn/nzaa077, 32440639 PMC7228438

[65] Jacka FN O’Neil A Opie R Itsiopoulos C Cotton S Mohebbi M . A randomised controlled trial of dietary improvement for adults with major depression (the ‘SMILES’ trial). BMC Med. (2017) 15:23. doi: 10.1186/s12916-017-0791-y, 28137247 PMC5282719

[66] Jeitler M Lauche R Hohmann C Choi KE Schneider N Steckhan N . A randomized controlled trial of fasting and lifestyle modification in patients with metabolic syndrome: effects on patient-reported outcomes. Nutrients. (2022) 14:3559. doi: 10.3390/nu14173559, 36079816 PMC9460714

[67] Kabiri SS Javanbakht Z Zangeneh M Moludi J Saber A Salimi Y . The effects of MIND diet on depression, anxiety, quality of life and metabolic and hormonal status in obese or overweight women with polycystic ovary syndrome: a randomised clinical trial. Br J Nutr. (2024) 1:1–14. doi: 10.1017/S0007114524001168 39465581

[68] Lee J Pase M Pipingas A Raubenheimer J Thurgood M Villalon L . Switching to a 10-day Mediterranean-style diet improves mood and cardiovascular function in a controlled crossover study. Nutrition. (2015) 31:647–52. doi: 10.1016/j.nut.2014.10.008, 25837207

[69] Long Parma DA Reynolds GL Muñoz E Ramirez AG. Effect of an anti-inflammatory dietary intervention on quality of life among breast cancer survivors. Support Care Cancer. (2022) 30:5903–10. doi: 10.1007/s00520-022-07023-4, 35380268 PMC10156067

[70] McMillan L Owen L Kras M Scholey A. Behavioral effects of a 10-day Mediterranean diet. Results from a pilot study evaluating mood and cognitive performance. Appetite. (2011) 56:143–7. doi: 10.1016/j.appet.2010.11.149, 21115083

[71] Miralles-Amorós L Vicente-Martínez M Martínez-Olcina M Asencio-Mas N Gonzálvez-Alvarado L Peñaranda-Moraga M . Study of different personalised dietary plans on eating behavior, body image and mood in young female professional handball players: a randomised controlled trial. Children. (2023) 10:259. doi: 10.3390/children10020259, 36832388 PMC9955722

[72] O’Connor LE Biberstine SL Paddon-Jones D Schwichtenberg AJ Campbell WW. Adopting a Mediterranean-style eating pattern with different amounts of lean unprocessed red meat does not influence short-term subjective personal well-being in adults with overweight or obesity. J Nutr. (2018) 148:1917–23. doi: 10.1093/jn/nxy235, 30517731 PMC7263834

[73] Papandreou P Amerikanou C Vezou C Gioxari A Kaliora AC Skouroliakou M. Improving adherence to the Mediterranean diet in early pregnancy using a clinical decision support system; a randomised controlled clinical trial. Nutrients. (2023) 15:432. doi: 10.3390/nu15020432, 36678303 PMC9866975

[74] Parletta N Zarnowiecki D Cho J Wilson A Bogomolova S Villani A . A Mediterranean-style dietary intervention supplemented with fish oil improves diet quality and mental health in people with depression: a randomized controlled trial (HELFIMED). Nutr Neurosci. (2019) 22:474–87. doi: 10.1080/1028415X.2017.1411320, 29215971

[75] Radkhah N Rasouli A Majnouni A Eskandari E Parastouei K. The effect of Mediterranean diet instructions on depression, anxiety, stress, and anthropometric indices: a randomized, double-blind, controlled clinical trial. Prev Med Rep. (2023) 36:102469. doi: 10.1016/j.pmedr.2023.102469, 37869542 PMC10587518

[76] Rumbo-Rodríguez L Zaragoza-Martí A Sánchez-SanSegundo M Ferrer-Cascales R Laguna-Pérez A Hurtado-Sánchez JA. Effectiveness of a two-year multicomponent intervention for the treatment of overweight and obesity in older people. Nutrients. (2022) 14:4762. doi: 10.3390/nu14224762, 36432449 PMC9696506

[77] Sánchez-Villegas A Martínez-González MA Estruch R Salas-Salvadó J Corella D Covas MI . Mediterranean dietary pattern and depression: the PREDIMED randomized trial. BMC Med. (2013) 11:208. doi: 10.1186/1741-7015-11-208, 24229349 PMC3848350

[78] Toobert DJ Glasgow RE Strycker LA Barrera M Ritzwoller DP Weidner G. Long-term effects of the Mediterranean lifestyle program: a randomized clinical trial for postmenopausal women with type 2 diabetes. Int J Behav Nutr Phys Act. (2007) 4:1. doi: 10.1186/1479-5868-4-1, 17229325 PMC1783667

[79] Torres SJ Nowson CA Worsley A. Dietary electrolytes are related to mood. Br J Nutr. (2008) 100:1038–45. doi: 10.1017/S0007114508959201, 18466657

[80] Wade AT Davis CR Dyer KA Hodgson JM Woodman RJ Keage HAD . A Mediterranean diet with fresh, lean pork improves processing speed and mood: cognitive findings from the MedPork randomised controlled trial. Nutrients. (2019) 11:1521. doi: 10.3390/nu11071521, 31277446 PMC6683093

[81] Wade AT Davis CR Dyer KA Hodgson JM Woodman RJ Keage HAD . A Mediterranean diet supplemented with dairy foods improves mood and processing speed in an Australian sample: results from the MedDairy randomized controlled trial. Nutr Neurosci. (2020) 23:646–58. doi: 10.1080/1028415X.2018.1543148, 30409085

[82] Wardle J Rogers P Judd P Taylor MA Rapoport L Green M . Randomized trial of the effects of cholesterol-lowering dietary treatment on psychological function. Am J Med. (2000) 108:547–53. doi: 10.1016/S0002-9343(00)00330-2, 10806283

[83] Wright KD Klatt MD Adams IR Nguyen CM Mion LC Tan A . Mindfulness in motion and dietary approaches to stop hypertension (DASH) in hypertensive African Americans. J Am Geriatr Soc. (2021) 69:773–8. doi: 10.1111/jgs.16947, 33227157 PMC8329944

[84] Yau KY Law PS Wong CN. Cardiac and mental benefits of Mediterranean-DASH intervention for neurodegenerative delay (MIND) diet plus Forest bathing (FB) versus MIND diet among older Chinese adults: a randomized controlled pilot study. Int J Environ Res Public Health. (2022) 19:14665. doi: 10.3390/ijerph192214665, 36429384 PMC9690744

[85] Selvaraj R Selvamani TY Zahra A Malla J Dhanoa RK Venugopal S . Association between dietary habits and depression: a systematic review. Cureus. (2022) 14:e32359. doi: 10.7759/cureus.32359, 36632273 PMC9828042

[86] O’Neill S Minehan M Knight-Agarwal CR Turner M. Depression, is it treatable in adults utilising dietary interventions? A systematic review of randomised controlled trials. Nutrients. (2022) 14:1398. doi: 10.3390/nu14071398, 35406011 PMC9003461

[87] Rudzińska A Perera I Gryglewska B Gąsowski J Piotrowicz K. Can the Mediterranean diet decrease the risk of depression in older persons – a systematic review. Psychiatr Pol. (2023) 57:339–54. doi: 10.12740/PP/OnlineFirst/140465, 36371734

[88] Solomou S Logue J Reilly S Perez-Algorta G. A systematic review of the association of diet quality with the mental health of university students: implications in health education practice. Health Educ Res. (2023) 38:28–68. doi: 10.1093/her/cyac035, 36441584 PMC9853940

[89] Suschana E Anderson T Hong C Narikatte A Silverberg J Sharma MS. The role of anti-inflammatory diets and supplementation in metabolic syndrome and symptom remission in adults with schizophrenia: a systematic review. Front Psych. (2025) 15:1506353. doi: 10.3389/fpsyt.2024.1506353, 39839138 PMC11747649

[90] Tan J Wang C Tomiyama AJ. Dietary approaches to stop hypertension (DASH) diet and mental well-being: a systematic review. Nutr Rev. (2023) 82:60–75. doi: 10.1093/nutrit/nuad038, 37085157 PMC10711436

[91] Bizzozero-Peroni B Martínez-Vizcaíno V Fernández-Rodríguez R Jiménez-López E Núñez De Arenas-Arroyo S Saz-Lara A . The impact of the Mediterranean diet on alleviating depressive symptoms in adults: a systematic review and meta-analysis of randomized controlled trials. Nutr Rev. (2025) 83:29–39. doi: 10.1093/nutrit/nuad176, 38219230

[92] DeSmet A Culot C Virani D Pabian S Ariño Mateo E Van Royen K . A systematic review and meta-analysis of the effectiveness of dietary interventions to enhance mental wellbeing in emerging adulthood. J Happiness Stud. (2024) 25:68. doi: 10.1007/s10902-024-00779-y

[93] Lassale C Batty GD Baghdadli A Jacka F Sánchez-Villegas A Kivimäki M . Healthy dietary indices and risk of depressive outcomes: a systematic review and meta-analysis of observational studies. Mol Psychiatry. (2019) 24:965–86. doi: 10.1038/s41380-018-0237-8, 30254236 PMC6755986

[94] Matison AP Mather KA Flood VM Reppermund S. Associations between nutrition and the incidence of depression in middle-aged and older adults: a systematic review and meta-analysis of prospective observational population-based studies. Ageing Res Rev. (2021) 70:101403. doi: 10.1016/j.arr.2021.101403, 34246793

[95] Molendijk M Molero P Ortuño Sánchez-Pedreño F Van Der Does W Angel M-GM. Diet quality and depression risk: a systematic review and dose-response meta-analysis of prospective studies. J Affect Disord. (2018) 226:346–54. doi: 10.1016/j.jad.2017.09.022, 29031185

[96] Psaltopoulou T Sergentanis TN Panagiotakos DB Sergentanis IN Kosti R Scarmeas N. Mediterranean diet, stroke, cognitive impairment, and depression: a meta-analysis. Ann Neurol. (2013) 74:580–91. doi: 10.1002/ana.23944, 23720230

[97] Shafiei F Salari-Moghaddam A Larijani B Esmaillzadeh A. Adherence to the Mediterranean diet and risk of depression: a systematic review and updated meta-analysis of observational studies. Nutr Rev. (2019) 77:230–9. doi: 10.1093/nutrit/nuy070, 30726966

[98] Tavakoly R Moosburner A Anheyer D Cramer H. Effect of different dietary patterns on patients with depressive disorders: a systematic review and Meta-analysis of randomized controlled trials. Nutrients. (2025) 17:563. doi: 10.3390/nu17030563, 39940421 PMC11820241

[99] Tolkien K Bradburn S Murgatroyd C. An anti-inflammatory diet as a potential intervention for depressive disorders: a systematic review and meta-analysis. Clin Nutr. (2019) 38:2045–52. doi: 10.1016/j.clnu.2018.11.007, 30502975

[100] Wang J Zhou Y Chen K Jing Y He J Sun H . Dietary inflammatory index and depression: a meta-analysis. Public Health Nutr. (2019) 22:654–60. doi: 10.1017/S1368980018002628, 30319085 PMC10260661

[101] Dinu M Pagliai G Casini A Sofi F. Mediterranean diet and multiple health outcomes: an umbrella review of meta-analyses of observational studies and randomised trials. Eur J Clin Nutr. (2018) 72:30–43. doi: 10.1038/ejcn.2017.58, 28488692

[102] Gianfredi V Dinu M Nucci D Eussen SJPM Amerio A Schram MT . Association between dietary patterns and depression: an umbrella review of meta-analyses of observational studies and intervention trials. Nutr Rev. (2023) 81:346–59. doi: 10.1093/nutrit/nuac058, 36240456

[103] Zupo R Castellana F Piscitelli P Crupi P Desantis A Greco E . Scientific evidence supporting the newly developed one-health labeling tool “med-index”: an umbrella systematic review on health benefits of mediterranean diet principles and adherence in a planeterranean perspective. J Transl Med. (2023) 21:755. doi: 10.1186/s12967-023-04618-1, 37885010 PMC10601192

[104] Altun A Brown H Szoeke C Goodwill AM. The Mediterranean dietary pattern and depression risk: a systematic review. Neurol Psychiatry Brain Res. (2019) 33:1–10. doi: 10.1016/j.npbr.2019.05.007

[105] Eliby D Simpson CA Lawrence AS Schwartz OS Haslam N Simmons JG. Associations between diet quality and anxiety and depressive disorders: a systematic review. J Affect Disord Rep. (2023) 14:100629. doi: 10.1016/j.jadr.2023.100629

[106] Jafari RS Behrouz V. Nordic diet and its benefits in neurological function: a systematic review of observational and intervention studies. Front Nutr. (2023) 10:1215358. doi: 10.3389/fnut.2023.1215358, 37645628 PMC10461010

[107] Madani S Ahmadi A Shoaei-Jouneghani F Moazen M Sasani N. The relationship between the Mediterranean diet and Axis I disorders: a systematic review of observational studies. Food Sci Nutr. (2022) 10:3241–58. doi: 10.1002/fsn3.2950, 36249971 PMC9548357

[108] Patrick RP Ames BN. Vitamin D and the omega-3 fatty acids control serotonin synthesis and action, part 2: relevance for ADHD, bipolar disorder, schizophrenia, and impulsive behavior. FASEB J. (2015) 29:2207–22. doi: 10.1096/fj.14-268342, 25713056

[109] Majewski M Kozlowska A Thoene M Lepiarczyk E Grzegorzewski WJ. Overview of the role of vitamins and minerals on the kynurenine pathway in health and disease. J Physiol Pharmacol Off J Pol Physiol Soc. (2016) 67:3–19. 27010891

[110] Zhu CB Blakely RD Hewlett WA. The Proinflammatory cytokines interleukin-1beta and Tumor necrosis factor-alpha activate serotonin transporters. Neuropsychopharmacology. (2006) 31:2121–31. doi: 10.1038/sj.npp.1301029, 16452991

[111] Lotrich FE El-Gabalawy H Guenther LC Ware CF. The role of inflammation in the pathophysiology of depression: different treatments and their effects. J Rheumatol Suppl. (2011) 88:48–54. doi: 10.3899/jrheum.110903, 22045979

[112] Najjar S Pearlman DM Alper K Najjar A Devinsky O. Neuroinflammation and psychiatric illness. J Neuroinflammation. (2013) 10:816. doi: 10.1186/1742-2094-10-43, 23547920 PMC3626880

[113] Calder PC. Omega-3 polyunsaturated fatty acids and inflammatory processes: nutrition or pharmacology? Br J Clin Pharmacol. (2013) 75:645–62. doi: 10.1111/j.1365-2125.2012.04374.x, 22765297 PMC3575932

[114] Simopoulos AP. Omega-3 fatty acids in inflammation and autoimmune diseases. J Am Coll Nutr. (2002) 21:495–505. doi: 10.1080/07315724.2002.10719248, 12480795

[115] Covidence. *Covidence Systematic Review Software*. Available online at: https://www.covidence.org (Accessed March 15, 2025).

[116] Thompson AJ Banwell BL Barkhof F Carroll WM Coetzee T Comi G . Diagnosis of multiple sclerosis: 2017 revisions of the McDonald criteria. Lancet Neurol. (2018) 17:162–173., 29275977 10.1016/S1474-4422(17)30470-2

