# Supplementary material for: Anti-inflammatory diets and mental health: a scoping review of randomized controlled trials and systematic evidence syntheses
Source: Front Nutr. 2026 Apr 13;13:1795350. doi: 10.3389/fnut.2026.1795350 (PMC13112677; doi:10.3389/fnut.2026.1795350)
Supplement: Supplementary file 1 [file Table_1.pdf]

Supplementary Table 1. Randomized Control Trial Outcomes and Results Breakdown by Conditions

Citation	Population Description	Diet	Measures	Multi-Modal	Depres-sion	Anxiety	Stress	PTS	Mood (total)	Mood Subscales									QOL	Severity of Mental Illness
										Anger	Tension	Confu-sion	Vigor	Depres-sion	Fatigue	Alert-ness	Calmness	Content-ment		
**Mental Health Conditions (n=10)**																				
Aucoin et al., 2023	Adult women with generalized anxiety disorder.	MedDiet (plus nutrition education, text messages, and group experiential cooking)	Anxiety (BAI), Quality of Life (PROMIS-29 v2.1)	Yes	N/A	Positive	N/A	N/A	N/A	N/A	N/A	N/A	N/A	N/A	N/A	N/A	N/A	N/A	Null	N/A
Bayes et al., 2022	Males (aged 18–25) diagnosed w/ major depressive disorder	MedDiet	Depression. (BDI), Quality of Life (WHOQOL-BREF)	No	Positive	N/A		N/A	N/A	N/A	N/A	N/A	N/A	N/A	N/A	N/A	N/A	N/A	Positive	N/A
Cabrera-Suárez et al., 2024	Adults w/1 depression episode w/in the last 5 years in total or partial clinical remission in the last 6 months	MedDiet enriched w/ extra virgin olive oil	Depression (BDI), Quality of Life (SF-36)	No	Mixed	N/A	N/A	N/A	N/A	N/A	N/A	N/A	N/A	N/A	N/A	N/A	N/A	N/A	N/A	N/A
Francis et al., 2019	Young adults w/ elevated levels of depression symptoms & a poor diet	Australian Guide to Healthy Eating + MedDiet recommendations	Depression (CES-D), Depression & Anxiety (DASS), Mood (POMS)	No	Positive^**	Positive^**	Positive^**	N/A	Null	Null (trending)	Null	Null	Null	Null	Null	N/A	N/A	N/A	N/A	N/A
Ibarra et al., 2014	Adults meeting DSM-IV criteria for MDD, dysthymic disorder, or bipolar disorder, currently on antidepressant treatment, & recent plasma micronutrient level assessment	MedDiet	Depression (BDI), Depression (HAM-D), Severity of the patient's illness (CGI)	No	Mixed	N/A	N/A	N/A	N/A	N/A	N/A	N/A	N/A	N/A	N/A	N/A	N/A	N/A	N/A	Null
Jacka et al., 2017	Adults meeting DSM-IV criteria for major depressive episode and scored ≥ 18 on the MADRS; ≤ 75d on a Dietary Screening Tool (DST)	ModiMedDiet (plus nutritional consulting)	Depression (MADRS-S), Anxiety & Depression (HADS), Severity of the patient's illness (CGI)	Yes	Postive*** & Positive^^**	Positive^^**	N/A	N/A	Null	NR	NR	NR	NR	NR	NR	N/A	N/A	N/A	N/A	Null
Radkhah et al., 2023	Individuals referred to a psychiatric clinic w/ depression, stress, or anxiety	MedDiet	Depression & Anxiety (DASS)	No	Mixed^*	Mixed^	Mixed^**	N/A	N/A	N/A	N/A	N/A	N/A	N/A	N/A	N/A	N/A	N/A	N/A	N/A

Parletta et al., 2019	Adults (age 18 and 65years) and diagnosed or self-reported depressive symptoms	MedDiet-style diet	(DASS) Depression & Anxiety, Quality of Life (AQo	No	Positive^	Null^	Null^	N/A	N/A	N/A	N/A	N/A	N/A	N/A	N/A	N/A	N/A	N/A	Positive	N/A
Sabet et al., 2021	Adults w/ mild to moderate depression	Nordic Diet	Depression (MADRS-S)	No	Null#	N/A	N/A	N/A	N/A	N/A	N/A	N/A	N/A	N/A	N/A	N/A	N/A	N/A	N/A	N/A
Staudacher et al., 2024	Adults w/ IBS & mild or moderate anxiety and/or depressive symptoms (n = 59)	MedDiet counselling	Depression & Anxiety, (HADS), Stress (PSS)	No	Positive^^	Positive^^	Positive	N/A	N/A	N/A	N/A	N/A	N/A	N/A	N/A	N/A	N/A	N/A	N/A	N/A
**Healthy Adults (≥ 18) (n= 6)**																				
Bourdel-Marchasson et al., 2020	Healthy older adults (65–80 year)	Anti-Inflamatory Diet	Depression (CES-D), Anxiety (STAI), Quality of Life (SF-36)	No	Positive	Null	N/A	N/A	N/A	N/A	N/A	N/A	N/A	N/A	N/A	N/A	N/A	N/A	Mixed	N/A
Carcelén-Fraile et al., 2024	Older adults aged 65 or older	Resistance Program + MedDiet	Anxiety & Depression (HADS), Stress(PSS)	No	Positive^^	Positive^^*	Positive*	N/A	N/A	N/A	N/A	N/A	N/A	N/A	N/A	N/A	N/A	N/A	N/A	N/A
Hardman et al., 2020	Older adults (age between 60–90) living independently	MedDiet (plus exercise)	Depression & Anxiety (DASS), Mood (POMS), Quality of Life (PWS)	Yes	Mixed (total score) ^	Null^	Null^	N/A	Null	N/A	N/A	N/A	N/A	N/A	N/A	N/A	N/A	N/A	Positive	N/A
Lee et al., 2015	Healthy adult females	MedDiet	Mood (VAS), Mood (POMS)	No	N/A	N/A	N/A	N/A	Null	Null	Null	Positive	Null	Null	Null	Positive	Null	Positive	N/A	N/A
McMillan et al., 2011	Healthy adult females	MedDiet (nutrient-dense)	Mood (VAS), Mood (POMS)	No	N/A	N/A	N/A	N/A	Null	Null	Null	Null	Positive	Null	Null	Positive	Null	Positive	N/A	N/A
Miralles-Amorós et al., 2023	Healthy Young adult female professional handball players	MedDiet	Mood (POMS)	No	N/A	N/A	N/A	N/A	NR	NR	NR	NR	NR	Null	NR	N/A	N/A	N/A	N/A	N/A
**Overweight or Obese Adults (n= 6)**																				
Arcan et al., 2024	WTC Health Program members (N = 62), 45–65 years, males 87%, body mass index (BMI) 27–45 kg/m2	MedDiet (plus nutrition education, text messages, and group experiential cooking)	PTSD (PCL-5)	Yes	N/A	N/A	N/A	Positive	N/A	N/A	N/A	N/A	N/A	N/A	N/A	N/A	N/A	N/A	N/A	N/A
Dolatkhah et al., 2022	Overweight or obese women (≥ 40 years) with mild to moderate knee osteoarthritis	Anti-inflammatory diet	Depressio n (BDI), Anxiety (BAI-I), Quality of Life (SF-36)	No	Positive	Positive	N/A	N/A	N/A	N/A	N/A	N/A	N/A	N/A	N/A	N/A	N/A	N/A	Null	N/A
	Fifty-two overweight or obese (BMI ≥ 25 kg/m2) women ages 18-45 in	Med-DASH	Depression & Anxiety (DASS), Quality of Life (PCOSQ)	No	Positive	Positive	N/A	N/A	N/A	N/A	N/A	N/A	N/A	N/A	N/A	N/A	N/A	N/A	Positive	N/A

Kabiri et al., 2024	reproductive age diagnosed with PCOS based on Rotterdam criteria	Intervention for Neurodegenerative Delay (MIND) diet																		
Long Parma et al., 2022	Adult women (BMI ≥ 25 kg/m2), early-stage (0-III) breast cancer survivors	Anti-inflammatory diet	Depression (CES-D), (PSS) Stress (PSS), Quality of Life (EWS)	No	Null	N/A	Positive	N/A	N/A	N/A	N/A	N/A	N/A	N/A	N/A	N/A	N/A	N/A	N/A	N/A
O'Connor et al., 2018	Adults w/ a BMI between 25 and 37	Medi-pattern	Mood (POMS), Quality of Life (SF-36)	No	N/A	N/A	N/A	N/A	Null	Null	Null	Null	Null	Null	Positive	N/A	N/A	N/A	Positive	N/A
Rumbo-Rodríguez et al., 2022	60 years w/ a body mass index (BMI) greater than 25 kg/m2	MedDiet based nutritional treatment (plus physical activity program, Nutritional education sessions + individualized dietary)	Depression (PHQ-9), Anxiety (GAD-7)	Yes	Null	Null	N/A	N/A	N/A	N/A	N/A	N/A	N/A	N/A	N/A	N/A	N/A	N/A	N/A	N/A
**Metabolic Disorders (n= 5)**																				
Daneshzad et al., 2022	Postmenopausal females w/ insulin-resistance condition	DASH Diet	Depression & Anxiety (DASS)	No	Positive*	Positive	Positive	N/A	N/A	N/A	N/A	N/A	N/A	N/A	N/A	N/A	N/A	N/A	N/A	N/A
Diekmann et al., 2019	Adults w/ Metabolic syndrome traits	Med-type diet (plus 30- min rest or walking)	Mood (MDBF)	Yes	N/A	N/A	N/A	N/A	Positive	NR	NR	NR	NR	NR	Positive	N/A	N/A	N/A	N/A	N/A
Golmohammadi et al., 2025	44 patients with type 2 diabetes and insomnia in Sanandaj, Iran.	MIND low-calorie diet (MLCD)	Depression, Stress, & Anxiety (DASS)	No	Positive	Null	Null	N/A	N/A	N/A	N/A	N/A	N/A	N/A	N/A	N/A	N/A	N/A	N/A	N/A
Jeitler et al., 2022	Patients w/ a metabolic syndrome, systolic hypertension and/or additional subclinical atherosclerosis	Plant-based MedDiet and a modified DASH diet	Depression, Anxiety, & Stress (CPSS), Anxiety & Depression (HADS), Mood (POMS), Quality of Life (SF-36)	No	Null^^	Null^^	Null*	N/A	NR	NR	Null	NR	Mixed*	Null	Mixed*	N/A	N/A	N/A	Null	N/A
Toobert et al., 2007	Postmenopausal women w/ type 2 diabetes	MedDiet (plus lifestyle program- physical activity, stress management, smoking cessation, and social support)	Depression (CES-D), Stress (PSS)	Yes	Null	Null	N/A	N/A	N/A	N/A	N/A	N/A	N/A	N/A	N/A	N/A	N/A	N/A	N/A	N/A
**Cardiovascular Disease or Risk (n= 4)**																				
Sanches-Villegas, 2013	Adults (55-80 yrs) at high cardiovascular disease risk	MedDiet+EVOO or MediDiet+nuts	Physician Diagnosis & reported by participants in follow-up interviews (Depression)	No	Null	N/A	N/A	N/A	N/A	N/A	N/A	N/A	N/A	N/A	N/A	N/A	N/A	N/A	N/A	N/A
	Adults (45–80 years old) w/ an increased risk of	MedDiet w/ lean pork		No	N/A	N/A	N/A	N/A	Null	NR	NR	NR	NR	NR	NR	N/A	N/A	N/A	Mixed	N/A

Wade, et al., 2019	developing cardiovascular disease (CVD)		Mood (POMS), Quality of Life (SF-36)																	
Wade, et al., 2020	Adults (45–80 years old) w/ an increased risk of developing cardiovascular disease (CVD)	MedDiet supplemented with diary foods	Mood (POMS), Quality of Life (SF-36)	No	N/A	N/A	N/A	N/A	Positive***	Positive***	Positive***	Positive***	NR	Positive***	NR	N/A	N/A	N/A	Mixed	N/A
Wardle, et al., 2000	Adults w/ mildly or moderately raised serum cholesterol levels	MedDiet	Depression (BDI), Anxiety (STAI) Stress (PSS), Mood (POMS) Mood,	No	Null	Null	Null	N/A	Null	N/A	N/A	N/A	N/A	N/A	N/A	N/A	N/A	N/A	N/A	N/A
**Elevated Blood Pressure or Hypertension (n=4)**																				
Torres, et al., 2008	Adults (>25 years old) and had a BP in the high/normal range	LNAHK Diet (modified DASH diet)	Mood (POMS)	No	N/A	N/A	N/A	N/A	Positive	Null	Positive	Null	Positive	Positive	Null	N/A	N/A	N/A	N/A	N/A
Torres & Nowson, 2012	Adults (>25 years old) and had a BP in the high/normal range	Moderate sodium DASH-type diet	Mood (POMS)	No	N/A	N/A	N/A	N/A	Positive	Positive	Positive	Positive	Positive	Positive	Positive	N/A	N/A	N/A	N/A	N/A
Wright, et al., 2021	African Americans w/ mild cognitive imparement & hypertension	MedDiet (plus Mindfulness)	Stress (PSS)	Yes	N/A	N/A	Null	N/A	N/A	N/A	N/A	N/A	N/A	N/A	N/A	N/A	N/A	N/A	N/A	N/A
Yau, et al., 2022	Older adults w/ hypertension	Mind Diet (plus nutritional counseling and forest bathing)	Anxiety (STAI), Mood (POMS)	Yes	N/A	Null	N/A	N/A	Null	Null	Null	Null	Null	Null	Null	N/A	N/A	N/A	N/A	N/A
**Pregnant Women (n= 3)**																				
Casas, et al., 2023	Pregnant women w/ a high risk of developing a small for gestational age newborn	MedDiet Diet w/ extra-virgin olive oil and walnuts	Anxiety (STAI), Stress (PSS)	No	N/A	Positive	Positive	N/A	N/A	N/A	N/A	N/A	N/A	N/A	N/A	N/A	N/A	N/A	N/A	N/A
Bozdoğan, et al., 2024	Pregnant women aged 20–40 years diagnosed with gestational diabetes mellitus (GDM)	MedDiet w/ Probiotic supplimentation	Depression (EPDS), Anxiety (PrAS)	No	Positive	Positive	N/A	N/A	N/A	N/A	N/A	N/A	N/A	N/A	N/A	N/A	N/A	N/A	N/A	N/A
Papandreou, et al., 2023	Healthy pregnant women	MediDiet	Depression & Anxiety (HADS)	No	Positive^^	Positive^^	N/A	N/A	N/A	N/A	N/A	N/A	N/A	N/A	N/A	N/A	N/A	N/A	N/A	N/A
**Spinal Cord Injury (n= 2)**																				
Allison & Ditor, 2015	Recovering from spinal cord injury	Anti-Inflamatory Diet	Depression (CES-D)	No	Positive***	N/A	N/A	N/A	N/A	N/A	N/A	N/A	N/A	N/A	N/A	N/A	N/A	N/A	N/A	N/A

Gazzellone, et al., 2023	≥ 1 year from either spinal cord injury (SCI) or neurological diagnosis (n=11)	Mad dog diet (anti-inflammatory)	Depression (CES-D)	No	Mixed	N/A	N/A	N/A	N/A	N/A	N/A	N/A	N/A	N/A	N/A	N/A	N/A	N/A	N/A	N/A
**Chronic Pain (n= 1)**																				
Casini, et al., 2024	100 Outpatients aged 18-65 years old with fibromyalgia; 84 completed the study	MedDiet (personalized)	Anxiety (SAS), Depression (SDS)	No	Positive*	Positive*	N/A	N/A	N/A	N/A	N/A	N/A	N/A	N/A	N/A	N/A	N/A	N/A	N/A	N/A
**Neurodegenerative Disease (n= 1)**																				
Felicetti, et al., 2025	53 adults older than 18 with a diagnosis of MS in accordance with the 2017 McDonald's criteria (Thompson et al., 2018) and body mass index (BMI) ≥25 kg/m2	MedDiet	Depression & Anxiety (Multiple Sclerosis Performance Test), Quality of Life (Neuro-QoL)	No	Null	Positive	N/A	N/A	N/A	N/A	N/A	N/A	N/A	N/A	N/A	Positive	N/A	N/A	N/A	Null	N/A

Positive- statistically Significant
Mixed- a mix of statistically significant and not statistically significant results
Null- not statistically significant
NR- Not Reported
This was a pilot feasibility study, t-tests were not performed by mean scores were assessed and participants reported decreased depression symptoms.
^ DSS
^^ HADS
* Small effect Size ≥ .2
** Medium Effect Size ≥ .5
*** Large Effect Size ≥ .8
