# Supplementary material for: Anti-inflammatory diets and mental health: a scoping review of randomized controlled trials and systematic evidence syntheses
Source: Front Nutr. 2026 Apr 13;13:1795350. doi: 10.3389/fnut.2026.1795350 (PMC13112677; doi:10.3389/fnut.2026.1795350)
Supplement: Supplementary file 2 [file Data_Sheet_1.pdf]

**SUPPLEMENTARY FILE S1: SEARCH STRATEGY BY DATABASE**

1. **PubMed**

("Diet, Mediterranean"[Mesh] OR Dietary Approaches To Stop Hypertension[Mesh] OR anti-inflammatory diet[tiab] OR Mediterranean diet[tiab] OR DASH[tiab] OR "Mediterranean-DASH Intervention for Neurodegenerative Delay"[tiab] OR "MIND diet"[tiab] OR nutritional intervention[tiab] OR healthy diet[tiab] OR Nordic diet[tiab]) AND (Cognition Disorders[Mesh] OR cogniti*[tiab] OR Depression[Mesh] OR Depressive Disorder[Mesh] OR Anxiety[Mesh] OR Anxiety Disorders[Mesh] OR depress*[tiab] OR Schizophrenia Spectrum and Other Psychotic Disorders[Mesh] OR Mood Disorders[Mesh] OR mood[tiab] OR psycho*[tiab] OR affective disorders[tiab] OR schizophrenia[tiab] OR neurodegenerat*[tiab] OR neuroprotect*[tiab] OR mental health[tiab] OR psychological health[tiab] OR memory[tiab] OR learn*[tiab])

2. **Embase**

('mediterranean diet'/exp OR 'dash diet'/exp OR 'anti-inflammatory diet':ti,ab,kw OR 'mediterranean diet':ti,ab,kw OR dash:ti,ab,kw OR 'mediterranean-dash intervention for neurodegenerative delay':ti,ab,kw OR 'mind diet':ti,ab,kw OR 'nordic diet'/exp OR 'nordic diet':ti,ab,kw OR 'nutritional intervention':ti,ab,kw OR 'healthy diet':ti,ab,kw) AND ('cognitive defect'/syn OR cogniti*:ti,ab,kw OR 'depression'/exp OR depress*:ti,ab,kw OR 'anxiety disorder'/exp OR 'anxiety'/exp OR 'schizophrenia'/exp OR schizophrenia:ti,ab,kw OR psycho*:ti,ab,kw OR 'mood disorder'/exp OR mood:ti,ab,kw OR 'affective disorders':ti,ab,kw OR neurodegenerat*:ti,ab,kw OR neuroprotect*:ti,ab,kw OR 'mental health':ti,ab,kw OR 'psychological health':ti,ab,kw OR memory:ti,ab,kw OR learn*:ti,ab,kw) NOT 'conference abstract'/it

3. **PsycINFO**

(ab("anti-inflammatory diet") OR ti("anti-inflammatory diet") OR ab("Mediterranean diet") OR ti("Mediterranean diet") OR ab(DASH) OR ti(DASH) OR ab("Mediterranean-DASH Intervention for Neurodegenerative Delay") OR ti("Mediterranean-DASH Intervention for Neurodegenerative Delay") OR ab("MIND diet") OR ti("MIND diet") OR ab("Nordic diet") OR ti("Nordic diet") OR ab("nutritional intervention") OR ti("nutritional intervention") OR ab("healthy diet" ) OR ti("healthy diet" )) AND (MAINSUBJECT.EXACT.EXPLODE("Cognitive Impairment") OR ab(cogniti*) OR ti(cogniti*) OR MAINSUBJECT.EXACT.EXPLODE("Major Depression") OR MAINSUBJECT.EXACT.EXPLODE("Depression (Emotion)") OR ab(depress*) OR ti(depress*) OR MAINSUBJECT.EXACT.EXPLODE("Anxiety") OR MAINSUBJECT.EXACT.EXPLODE("Anxiety Disorders") OR MAINSUBJECT.EXACT.EXPLODE("Affective Disorders") OR ab(mood) OR ti(mood) OR ab("affective disorders") OR ti("affective disorders") OR MAINSUBJECT.EXACT.EXPLODE("Psychosis") OR ab(schizophrenia) OR ti(schizophrenia) OR ab(psycho*) OR ti(psycho*) OR ab(neurodegenerat*) OR ti(neurodegenerat*) OR ab(neuroprotect*) OR ti(neuroprotect*) OR ab("mental health") OR ti("mental health") OR ab("psychological health") OR ti("psychological health") OR ab(memory) OR ti(memory) OR ab(learn*) OR ti(learn*))

4. **CINAHL**

( (MH "Mediterranean Diet") OR (MH "DASH Diet") OR (MH "Diet, Nordic") OR (AB mediterranean diet OR TI mediterranean diet ) OR (AB DASH diet OR TI DASH diet ) OR (AB Nordic diet OR TI Nordic diet) OR (AB anti-inflammatory diet OR TI anti-inflammatory diet) OR (AB nutritional intervention OR TI nutritional intervention) OR (AB "Healthy diet" OR TI "healthy diet") OR (AB "MIND diet" OR TI "MIND diet") OR (AB mediterranean-dash intervention for neurodegenerative delay

OR TI mediterranean-dash intervention for neurodegenerative delay) ) AND ( (MH "Neurodegenerative Diseases+") OR (MH "Anxiety Disorders+") OR (MH "Anxiety+") OR (MH "Depression+") OR (MH "Schizophrenia+") OR (MH "Affective Disorders") OR (MH "Delirium, Dementia, Amnestic, Cognitive Disorders+") OR (MH "Learning+") OR (AB cogniti* OR TI cogniti*) OR (AB depress* OR TI depress*) OR (AB schizophrenia OR TI schizophrenia) OR (AB psycho* OR TI psycho*) OR (AB mood OR TI mood) OR (AB affective disorders OR TI affective disorders) OR (AB neurodegenerat* OR TI neurodegenerat*) OR (AB neuroprotect* OR TI neuroprotect*) OR (AB mental health OR TI mental health) OR (AB "psychological health" OR TI "psychological health") OR (AB memory OR TI memory) OR (AB learn* OR TI learn*) )

5. **Web of Science**

("Mediterranean diet" OR "DASH diet" OR "Nordic diet" OR TOPIC:(anti-inflammatory NEAR/4 diet) OR TOPIC:(nutritional NEAR/2 intervention*) OR "healthy diet" OR "mind diet" OR mediterranean-dash intervention for neurodegenerative delay) AND (neurodegenerat* OR anxiety OR depress* OR schizophrenia OR affective disorder* OR dementia OR bipolar OR learn* OR cogniti* OR psycho* OR mood OR neuroprotect* OR mental health OR psychological health OR memory)
